# Supplementary material for: Photocatalytic radical defluoroalkylation of unactivated alkenes via distal heteroaryl ipso-migration
Source: Commun Chem. 2020 Aug 4;3:98. doi: 10.1038/s42004-020-00354-5 (PMC9814454; doi:10.1038/s42004-020-00354-5)
Supplement: Supplementary file 1 — Supplementary Information [file 42004_2020_354_MOESM1_ESM.pdf]

Supporting Information

Photocatalytic Radical Defluoroalkylation of Unactivated Alkenes *via* Distal Heteroaryl *ipso*-Migration

Xin Yuan[1,2], Kai-Qiang Zhuang[1,2], Yu-Sheng Cui[1,2], Long-Zhou Qin[1,2], Qi Sun[1,2], Xiu Duan[1,2], Lin Chen[1,2], Ning Zhu[1,2], Guigen Li[3, 4], Jiang-Kai Qiu[1,2*] & Kai Guo[1,2*]

[1] Biotechnology and Pharmaceutical Engineering, Nanjing Tech University, Nanjing 211816, P. R. China, E-mail: guok@njtech.edu.cn

[2] State Key Laboratory of Materials-Oriented Chemical Engineering, Nanjing Tech University, Nanjing 211800, P. R. China.

[3] Institute of Chemistry & Biomedical Science, Nanjing University, Nanjing 210093, P.R. China, Email: guigen.li@ttu.edu.cn

[4] Department of Chemistry and Biochemistry, Texas Tech University, Lubbock, TX 79409-1061, USA.
[*] Co-corresponding authors. These authors jointly supervised this work.

Table of contents

Table of contents 2
1. General Information 3
2. General procedure 3
3. X-ray Crystallography Structure of Compounds **3a** 4
4. Optimization of reaction conditions 6
5. Mechanistic investigation 9
6. Fluorescence Quenching Experiment 13
7 Product derivation 18
8. NMR Spectra 18
9. The Sprctra of products 30
10. Supplementary References 75

Supplementary Methods

1. General information

All commercially available reagents and solvents used in this study were purchased from Alfa Aesar, Sigma Aldrich or Energy Chemical and used without further purification. All reactions were conducted under Ar atmosphere while subject to irradiation from Blue LEDs (455 nm, 50 W). Thin layer chromatography (TLC) was measured on EMD preloaded plates (silica gel 60 F254) and was visualized under ultraviolet light (254 nm and 365 nm). Column chromatography was performed with silica gel (200-300 mesh).

All new compounds were characterized by ^{1}H, ^{13}C and ^{19}F NMR spectroscopy and mass spectrometry. NMR spectra were recorded on a Bruke Avance operating for ^{1}H NMR at 400 MHz, ^{13}C NMR at 100 MHz, and ^{19}F NMR at 376 MHz. Chemical shifts (δ) were reported in ppm referenced to Tetramethylsilane (TMS) as internal standard. NMR spectra uses the following abbreviations to describe the multiplicity: s = singlet, d = doublet, t = triplet, q = quartet, m = multiplet, dd = double doublet, td = triple doublet. Coupling constants (*J*) were reported in hertz (Hz). Known products were characterized by comparing to the corresponding ^{1}H NMR, ^{13}C NMR and ^{19}F NMR from literature. NMR data was processed using the MestReNova 9.0.1 software package. High resolution mass spectra were obtained on Agligent Technologies 6520 Accurate Series Q-TOF equipped with ESI.

2. General procedure

General procedure for visible light induced single C(sp^{3})-F bond cleavage of trifluoromethylarenes

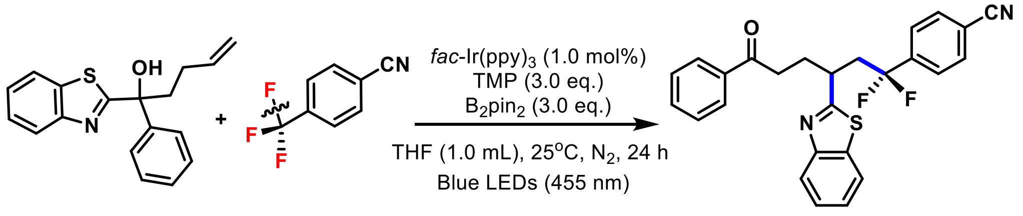

1-(benzo[*d*]thiazol-2-yl)-1-phenylpent-4-en-1-ol **1a**[1] (0.2 mmol, 59.0 mg, 2.0 eq.), 4-(Trifluoromethyl)benzonitrile **2a** (0.1 mmol, 17.1 mg, 1.0 eq.) and *fac*-Ir(ppy)$_3$ (0.001 mmol, 0.7 mg, 1.0 mol%) were added into a 25 mL snap vial equipped with a stirring bar. The vial was purged with N_2 for three times via syringe needle. Then TMP (0.3 mmol, 42.3 mg ,51 μL, 3.0 eq.), dry THF (1.0 mL) and B_2pin_2 (0.3 mmol, 76.2 mg, 3.0 eq.) were added sequentially by syringes. Then the reaction mixture was irradiated through the bottom side of the vial by Blue LEDs at 25°C. All the reaction was stopped, the mixture was transferred into a separating funnel and diluted by DCM (30 mL). The organic layer was washed by H_2O (10 mLx2) and brine (10 mL), dried over anhydrous Na_2SO_4, and then concentrated under

reduced pressure. The resulting residue was purified by flashed column chromatography to obtain the desired product.

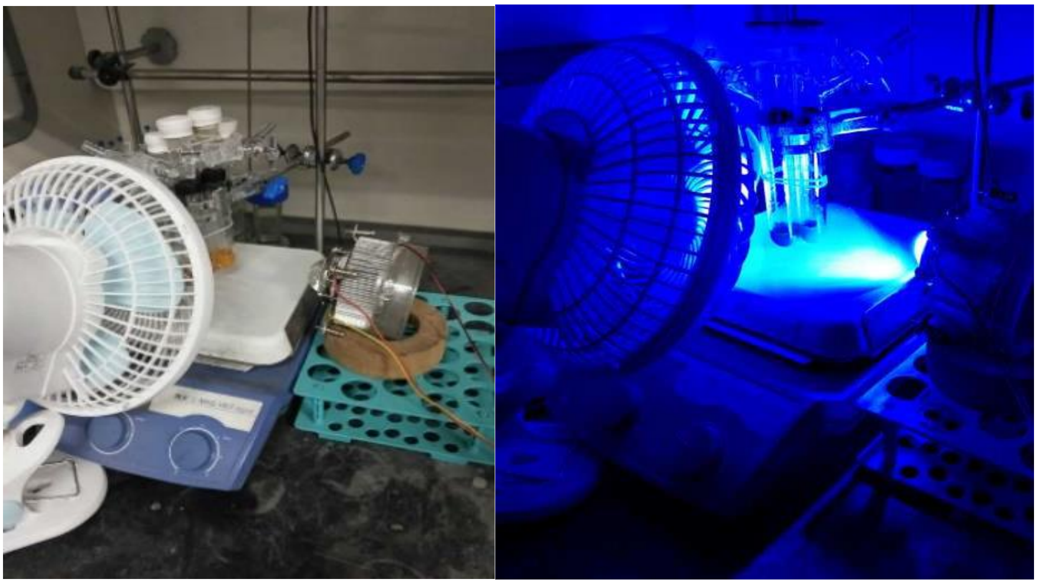

Supplementary Figure 1. Blue LED reactors

3. X-ray Crystallography Structure of Compounds 3a

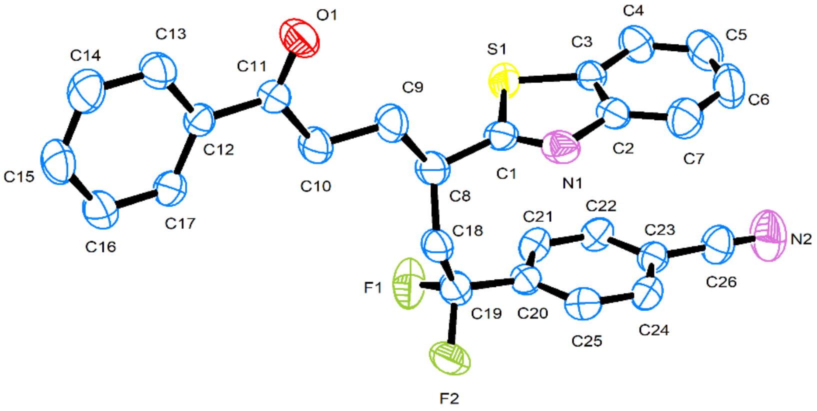

Supplementary Figure 2. The ORTEP Drawing of **3a**

CCDC 1867225 (**3a**) contains the supplementary crystallographic data for this paper. This data can be obtained free of charge from The Cambridge Crystallographic Data Centre.

Supplementary Table 1 Crystal data and structure refinement for **3a**

Identification code	**3a**
Empirical formula	$C_{26}H_{20}F_2N_2OS$
Audit creation method	SHELXL-97
Formula weight	446.50
Length a	8.6140 (7)
Length b	27.034 (2)
Length c	9.3247 (8)
Angle alpha	90.00
Angle beta	97.680 (2)
Angle gamma	90.00
volume	2151.9 (3)
Formula units Z	4
Measurement temperature	298 (2)
Measurement reflns used	2408
Measurement theta min	2.329
Measurement theta max	21.88
Crystal size max	0.45
Crystal size mid	0.37
Crystal size min	0.11
Crystal density diffrn	1.378
F 000	928
Absorpt coefficient mu	0.189
Absorpt correction type	Multi scan
Absorpt correction T min	0.9197
Absorpt correction T max	0.9795
Absorpt process details	sadabs
Radiation wavelength	0.71073
Reflns number	10655
Space group name H-M	P2(1)/n

4. Optimization of reaction conditions

Supplementary Table 2 Screening of Photocatalysts

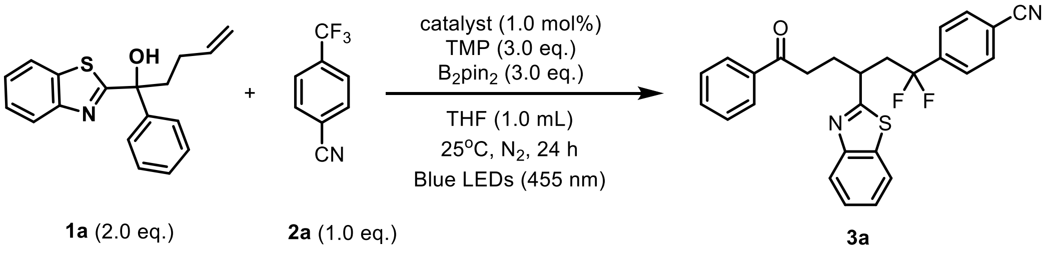

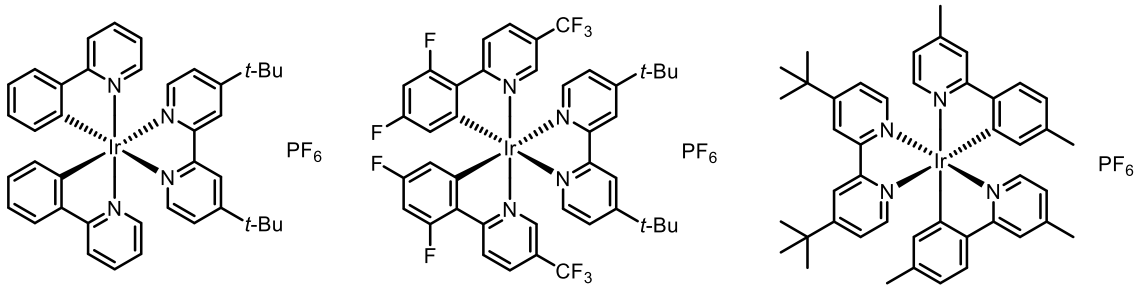

$[Ir(dtbbpy)(ppy)_2]PF_6$ **PC1**

$Ir\{dF(CF_3)ppy\}_2(dtbpy)PF_6$ **PC2**

$Ir(dmppy)_2(dtbbpy)PF_6$ **PC3**

Entry [a]	Catalyst	Yield [b]
1	$[Ir(ppy)_2(dtbbpy)]PF_6$ (**PC1**)	3%
2	$Ir\{dF(CF_3)ppy\}_2(dtbpy)PF_6$ (**PC2**)	N.D.
3	$Ir(dmppy)_2(dtbbpy)PF_6$ (**PC3**)	N.D.
4	$Ru(bpy)Cl_2 \cdot 6H_2O$ (**PC4**)	N.D.
5	$[Ru(bpz)_3][PF_6]_2$ (**PC5**)	N.D.
6	Rhodamine 6G (**PC6**)	N.D.
7	Rhodamine B (**PC7**)	N.D.
8	Mes-Acr^+ (**PC8**)	N.D.
9	-	N.D.

[a] Standard condition: **1a** (2.0 eq., 0.2 mmol), **2a** (1.0 eq., 0.1 mmol), TMP (3.0 eq., 0.3 mmol), B_2Pin_2 (3.0 eq., 0.3 mmol), photocatalysts (1.0 mol%). [b] Isolated yield is based on **2a**. N.D. = not detected.

Supplementary Table 3 Screening of Amines and F^- Scavengers

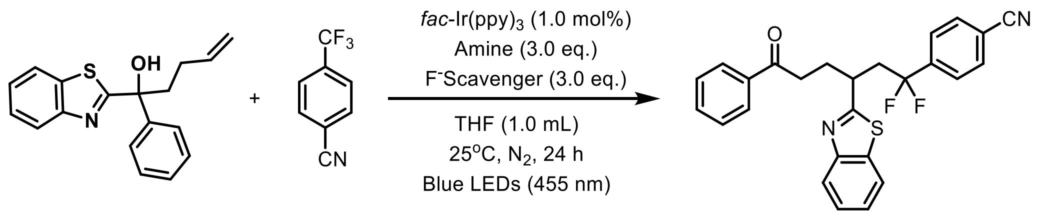

1a (2.0 eq.) **2a** (1.0 eq.) **3a**

Entry [a]	Amine	F^- Scavengers	Yield (%) [b]
1	DIPEA	HBpin	15
2	Et_3N	HBpin	N.R.
3	quinuclidine	HBpin	17
4	piperidine	HBpin	N.R.
5	-	HBpin	N.R.
6	TMP	HBpin	42
7	TMP	-	N.R.
8	TMP	B_2Pin_2	84
9	TMP	$HSiEt_3$	N.R.
10	TMP	$BF_3.Et_2O$	N.R.

[a] Standard condition: **1a** (2.0 eq., 0.2 mmol), **2a** (1.0 eq., 0.1 mmol), TMP (3.0 eq., 0.3 mmol), B_2Pin_2 (3.0 eq., 0.3 mmol), photocatalysts (1.0 mol%). [b] Isolated yield is based on **2a**. N.R. = no reaction.

Supplementary Table 4 Screening of additive

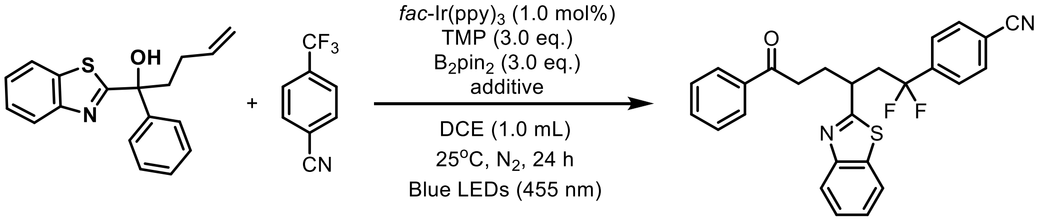

1a (2.0 eq.) **2a** (1.0 eq.) **3a**

Entry [a]	additive	Yield (%) [b]
1 [c]	Cs_2CO_3	14

2 [c]	K_2CO_3	12
3 [c]	K_2HPO_4	13
4 [c]	KH_2PO_4	7
5 [d]	H_2O	30
6 [e]	H_2O	N.D.
7 [d]	CH_3COOH	27
8 [e]	CH_3COOH	N.R.

[a] Standard condition: **1a** (2.0 eq., 0.2 mmol), **2a** (1.0 eq., 0.1 mmol), TMP (3.0 eq., 0.3 mmol), B_2Pin_2 (3.0 eq., 0.3 mmol), photocatalysts (1.0 mol%). [b] Isolated yield is based on **2a**. N.D. = not detected. N.R. = no reaction. [c] 2.0 eq. [d] 0.2 mL. [e] 1.0 mL.

Supplementary Table 5 Screening of solvents

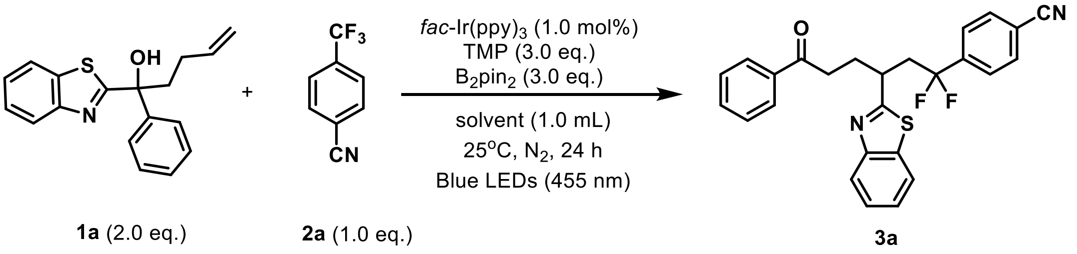

Entry [a]	Solvents	Yield (%) [b]
1	DCM	32
2	MeCN	34
3	THF	66
4 [c]	THF	18
5	DMF	28
6	DMAC	36.
7	1,4-dioxane	N.R.
8	DMPU	50
9	methanol	48
10	acetone	42
11 [d]	DCE	N.R.

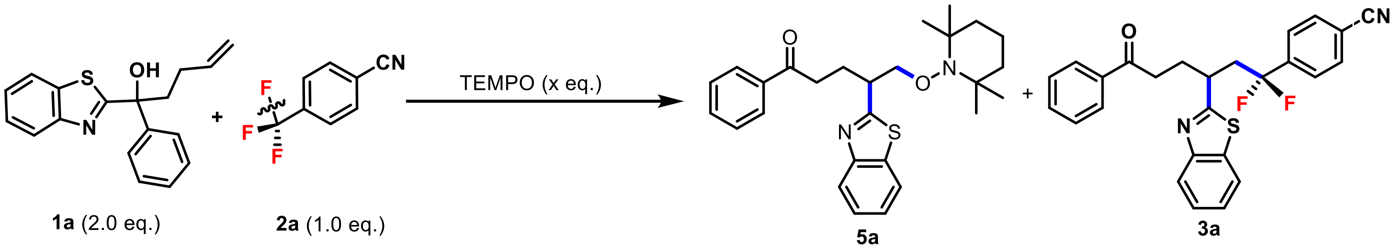

	5a	3a
x=0.5 eq.	detected by HRMS	not detected
x=2.0 eq.	detected by HRMS	not detected

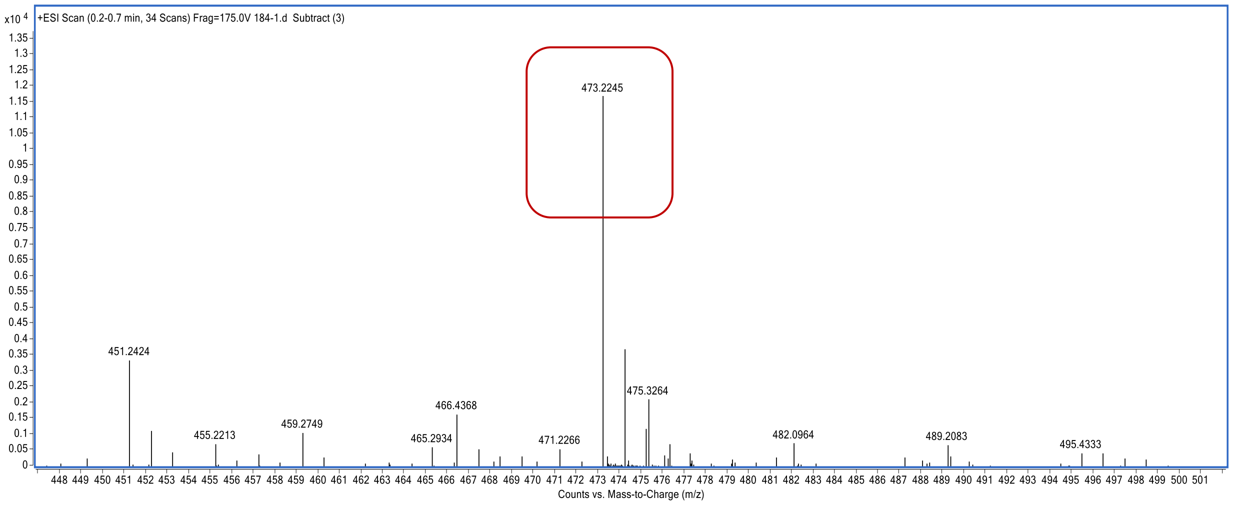

12 [e]	DCE	N.R.

[a] Standard condition: **2a** (1.0 eq., 0.1 mmol), **1a** (2.0 eq., 0.2 mmol), TMP (3.0 eq., 0.3 mmol), B_2Pin_2 (3.0 eq., 0.3 mmol), photocatalysts (1.0 mol%). [b] Isolated yield is based on **2a**. N.R. = no reaction. [c] 45 °C. [d] Air. [e] without irradiation.

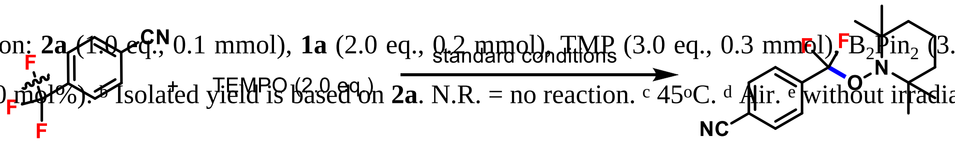

2a (1.0 eq.)

5 (detected by HRMS)

5. Mechanistic investigation

a) Radical Trapping Experiment by Tempo

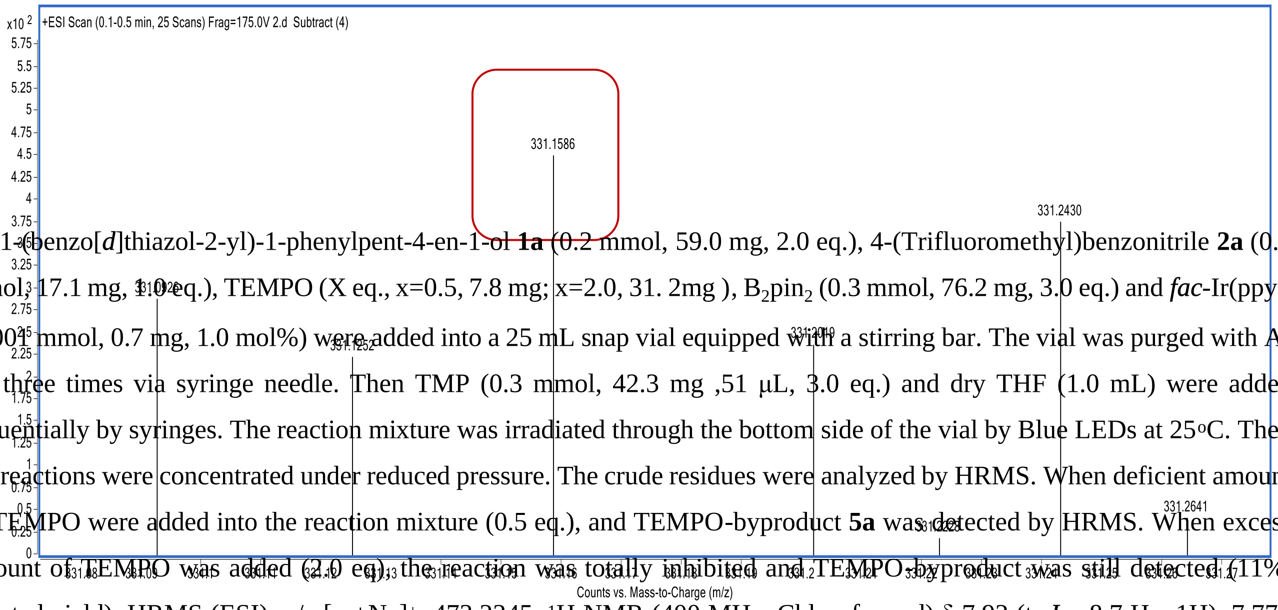

1-(benzo[*d*]thiazol-2-yl)-1-phenylpent-4-en-1-ol **1a** (0.2 mmol, 59.0 mg, 2.0 eq.), 4-(Trifluoromethyl)benzonitrile **2a** (0.1 mmol, 17.1 mg, 1.0 eq.), TEMPO (X eq., x=0.5, 7.8 mg; x=2.0, 31. 2mg), B_2pin_2 (0.3 mmol, 76.2 mg, 3.0 eq.) and *fac*-Ir(ppy)$_3$ (0.001 mmol, 0.7 mg, 1.0 mol%) were added into a 25 mL snap vial equipped with a stirring bar. The vial was purged with Ar for three times via syringe needle. Then TMP (0.3 mmol, 42.3 mg ,51 µL, 3.0 eq.) and dry THF (1.0 mL) were added sequentially by syringes. The reaction mixture was irradiated through the bottom side of the vial by Blue LEDs at 25 °C. Then the reactions were concentrated under reduced pressure. The crude residues were analyzed by HRMS. When deficient amount of TEMPO were added into the reaction mixture (0.5 eq.), and TEMPO-byproduct **5a** was detected by HRMS. When excess amount of TEMPO was added (2.0 eq), the reaction was totally inhibited and TEMPO-byproduct was still detected (11%, isolated yield). HRMS (ESI) m/z [m+Na]$^+$: 473.2245. ^{1}H NMR (400 MHz, Chloroform-d) δ 7.92 (t, *J* = 8.7 Hz, 1H), 7.77 – 7.71 (m, 1H), 7.62 (t, *J* = 7.6 Hz, 2H), 7.35 (q, *J* = 7.2 Hz, 1H), 7.29 – 7.20 (m, 3H), 7.15 (d, *J* = 7.2 Hz, 1H), 4.35 – 4.44 (m, 1H), 4.03 – 3.84 (m, 2H), 3.24 – 3.10 (m, 1H), 2.56 – 2.39 (m, 1H), 2.04 – 1.86 (m, 2H), 1.38 – 0.90 (m, 18H).

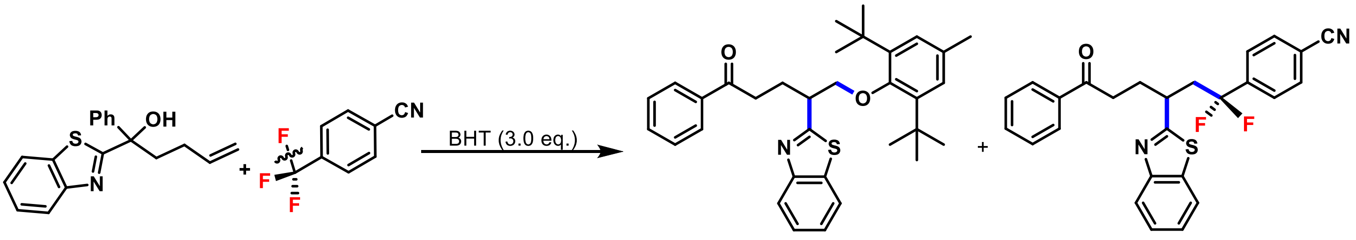

1a (2.0 eq.) **2a** (1.0 eq.) **6** (detected by HRMS) **3a** (N.D.)

Supplementary Figure 3. HRMS (ESI) data of **5a**

b) Radical Trapping Experiment by Tempo without **1a**

4-(Trifluoromethyl)benzonitrile **2a** (0.1 mmol, 17.1 mg, 1.0 eq.), TEMPO (2.0 eq., 31. 2mg), B_2pin_2 (0.3 mmol, 76.2 mg, 3.0 eq.) and *fac*-Ir(ppy)$_3$ (0.001 mmol, 0.7 mg, 1.0 mol%) were added into a 10 mL snap vial equipped with a stirring bar. The vial was purged with N_2 for three times via syringe needle. Then TMP (0.3 mmol, 42.3 mg ,51 µL, 3.0 eq.) and dry THF (1.0 mL) were added sequentially by syringes. The reaction mixture was irradiated through the bottom side of the vial by Blue LEDs at 25°C. Then the reactions were concentrated under reduced pressure. The crude residues were analyzed by HRMS. The TEMPO trapped aryldifluoromethyl radical **5** was detected by HRMS. HRMS (ESI) m/z [M+Na]$^{+}$: 331.1586.

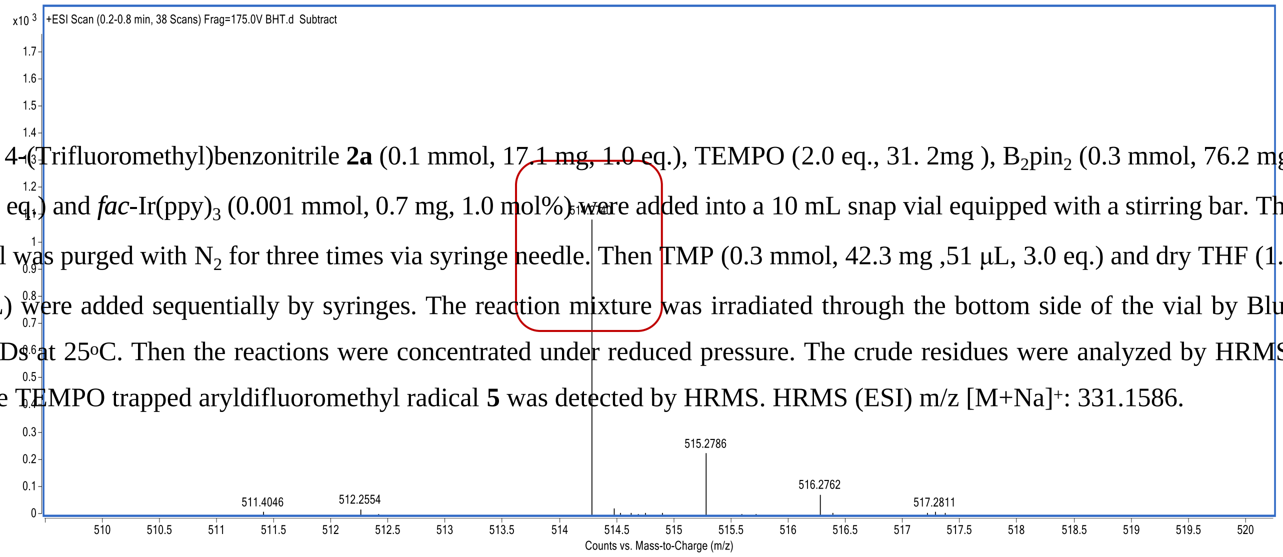

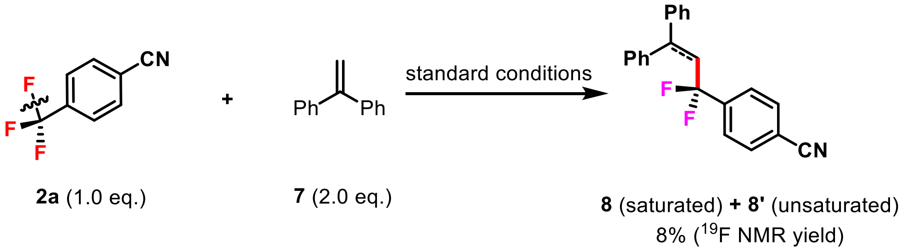

Supplementary Figure 4. HRMS (ESI) data of **5**

c) Trapping Experiment by BHT

1-(benzo[*d*]thiazol-2-yl)-1-phenylpent-4-en-1-ol **1a** (0.2 mmol, 35.0 mg, 2.0 eq.), 4-(Trifluoromethyl)benzonitrile **2a** (0.1 mmol, 17.1 mg, 1.0 eq.), BHT (0.3 mmol, 66.1 mg, 3.0 eq.), B_2pin_2 (0.3 mmol, 76.2 mg, 3.0 eq.) and *fac*-Ir(ppy)$_3$ (0.001 mmol, 0.7 mg, 1.0 mol%) were added into a 25 mL snap vial equipped with a stirring bar. The vial was purged with Ar for three times via syringe needle. Then TMP (0.3 mmol, 42.3 mg ,51 µL, 3.0 eq.) and dry THF (1.0 mL) were added sequentially by syringes.

The reaction mixture was irradiated through the bottom side of the vial by Blue LEDs at 25 ºC. Then the reactions were concentrated under reduced pressure. The crude residues were analyzed by HRMS. BHT trapped aryldifluoromethyl radical **6** was detected by HRMS. HRMS (ESI) m/z $[M+H]^+$: 514.2740.

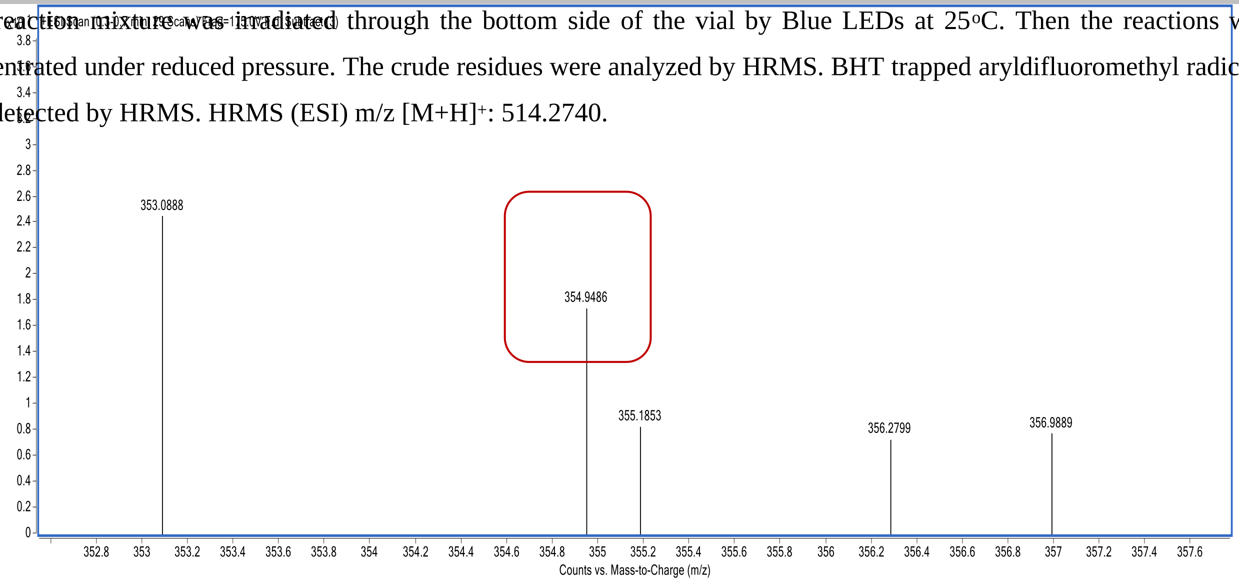

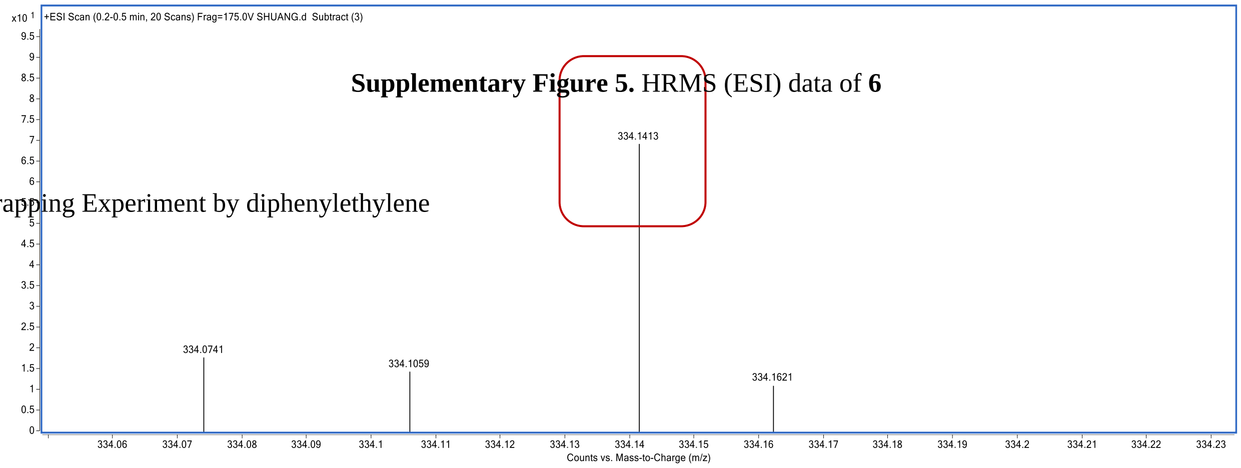

Supplementary Figure 5. HRMS (ESI) data of **6**

d) Trapping Experiment by diphenylethylene

4-(Trifluoromethyl)benzonitrile **2a** (0.1 mmol, 17.1 mg, 1.0 eq.), diphenylethylene **7** (0.2 mmol, 36 mg, 2.0 eq.), B_2pin_2 (0.3 mmol, 76.2 mg, 3.0 eq.) and *fac*-Ir(ppy)$_3$ (0.001 mmol, 0.7 mg, 1.0 mol%) were added into a 10 mL snap vial equipped with a stirring bar. The vial was purged with Ar for three times via syringe needle. Then TMP (0.3 mmol, 42.3 mg , 51.0 μL, 3.0 eq.) and dry THF (1.0 mL) were added sequentially by syringes. The reaction mixture was irradiated through the bottom side of the vial by Blue LEDs at 25 ºC. Then the reactions were concentrated under reduced pressure. the crude residues were analyzed by ^{19}F NMR (42.1 mg of $PhOCF_3$ as internal standard) and HRMS (ESI). The conversion of **2a** was 38%. A mixture of saturated and unsaturated trapping products **8** and **8'** were obtained in 8% yield. ^{19}F NMR (376 MHz, $CDCl_3$) δ 81.6, 87.7 (unsaturated), 94.3 (saturated). HRMS (ESI) m/z $[M+H]^+$: 354.9486 (unsaturated), $[M+H]^+$: 334.1413 (saturated).

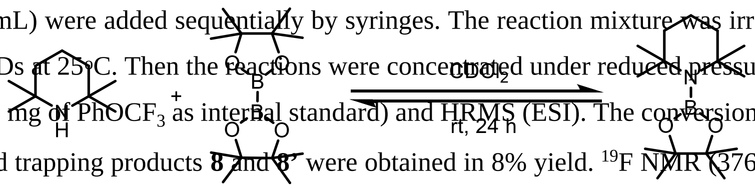

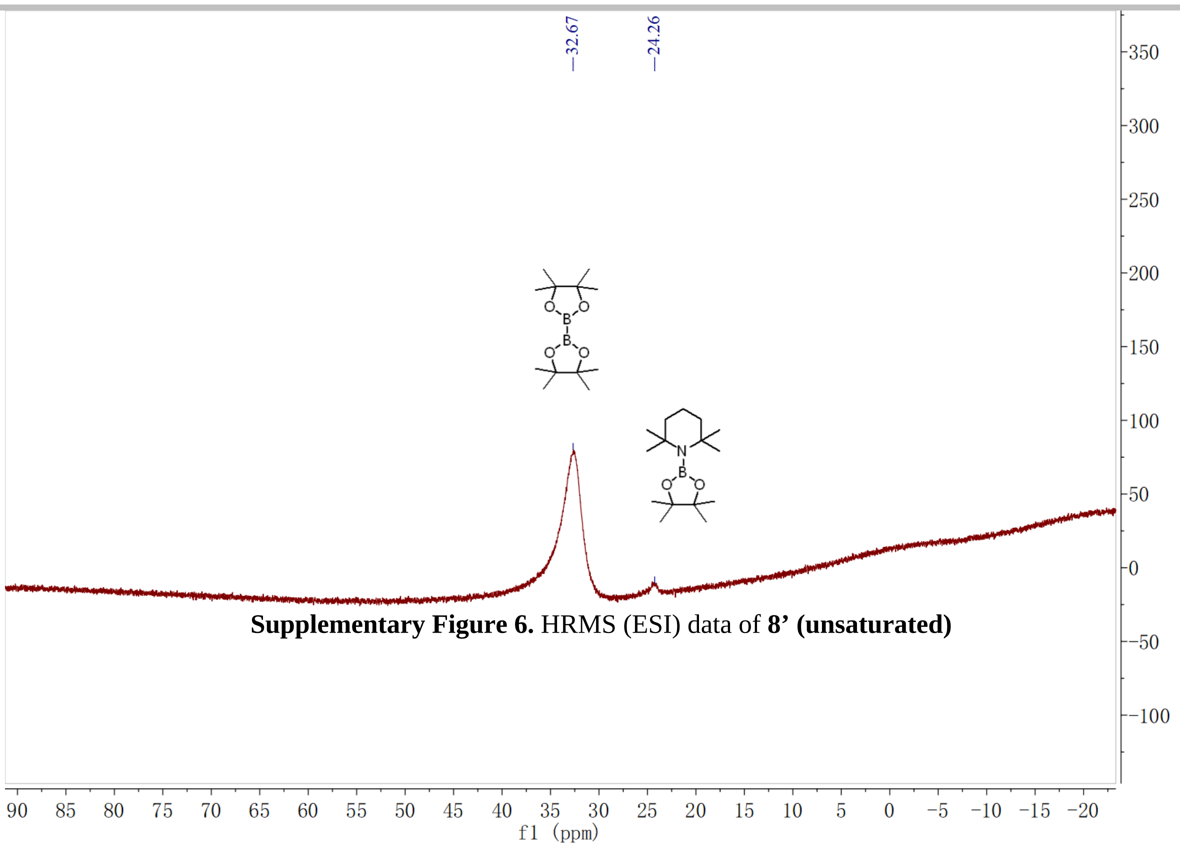

Supplementary Figure 6. HRMS (ESI) data of **8' (unsaturated)**

Supplementary Figure 7. HRMS (ESI) data of **8 (saturated)**

e) Reaction between TMP and B_2Pin_2

Under Ar atmosphere, TMP (42.3 mg, 51 μL, 0.3 mmol) was dissolve into CD_2Cl_2 (1.0 mL) in an oven-dried 5 mL snap vial equipped with a stirring bar. Then B_2Pin_2 (76.2 mg, 0.3 mmol) was added to above solution. The mixture was stirred for 24 h and detected by ^{11}B NMR.

The signal at 24.3 ppm in the ^{11}B NMR spectrum represents the neutral amino-borane species and appears whenever TMP and B_2pin_2 are present. The free TMP and B_2pin_2 reach an equilibrium with the amino-borane adduct. However, most of the free TMP and B_2pin_2 still remain even after a long reaction time (24 h) and only a small amount of amino-borane adduct can be detected.

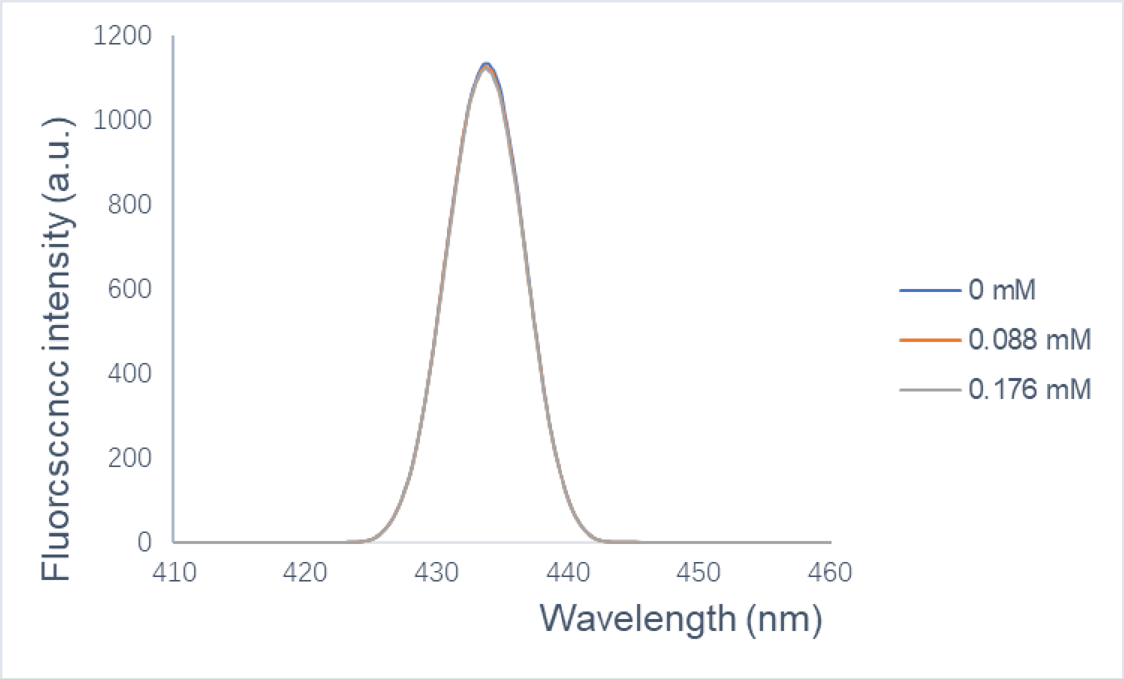

Supplementary Figure 8. ^{11}B NMR of the reaction between TMP (0.3 mmol) and B_2Pin_2 (0.3 mmol) in CD_2Cl_2 (1.0 mL) for 24 h under N_2 atmosphere.

6. Fluorescence Quenching Experiment

Emission intensities were recorded using a F-7000 FL Spectrophotometer (serial number: 2574-001). All quenching data was recorded using a 1.00 cm slit cuvette, PMT voltage 500 v, scan speed 1200 nm/min. In a typical experiment, the CH_3CN solution of *fac*-Ir(ppy)$_3$ (0.5 mM) was added the appropriate amount of quencher. After degassing with nitrogen for 10 min, the emission spectrum of the sample was collected.

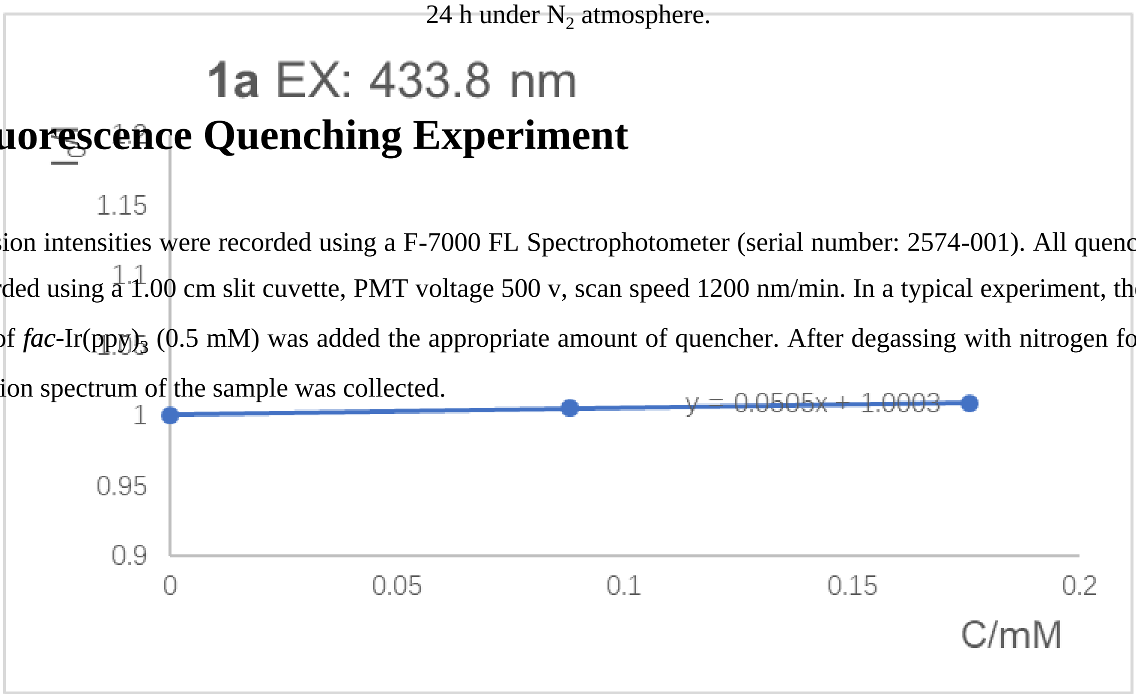

6.1 Emission quenching by **1a**

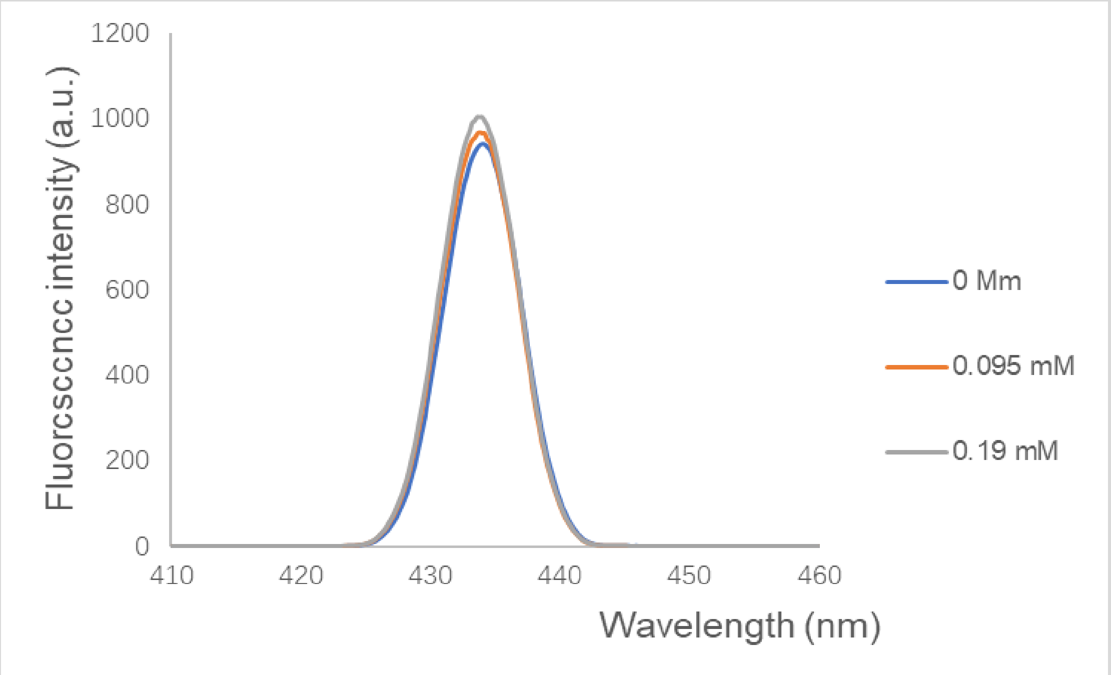

Supplementary Figure 9. Fluorescence titration of 0.5 mM *fac*-Ir(ppy)$_3$ (CH_3CN) with increasing concentration of **1a** (0 – 0.176 mM) excited at 450 nm.

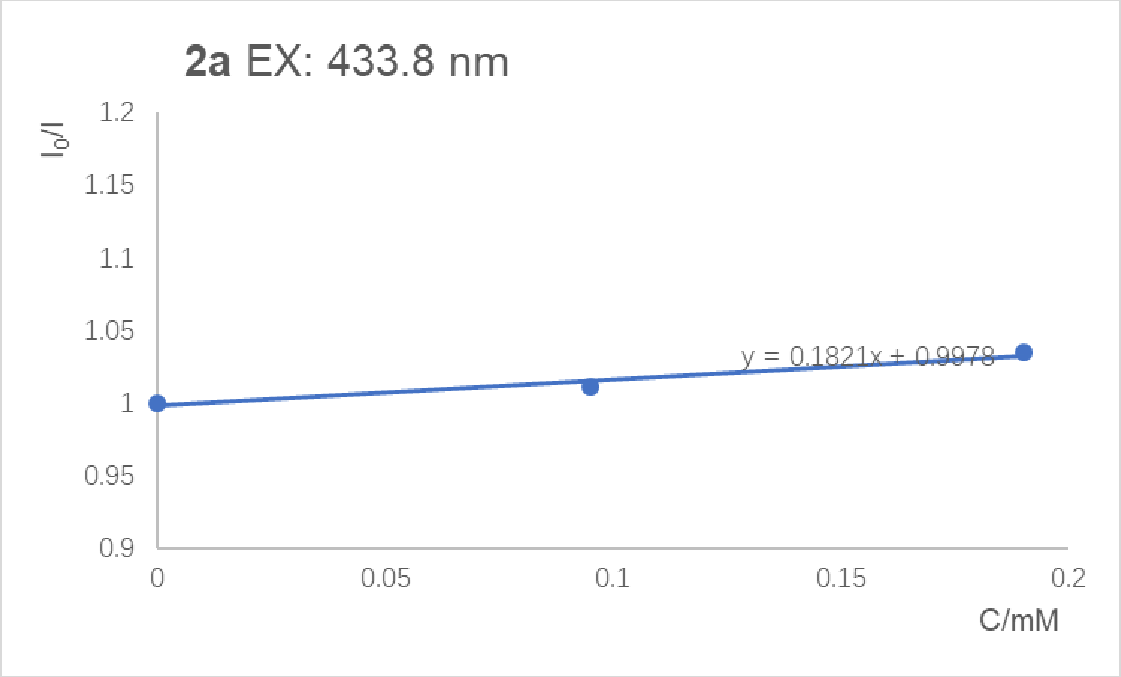

Supplementary Figure 10. Fluorescence titration of 0.5 mM *fac*-Ir(ppy)$_3$ (CH_3CN) with increasing concentration of **1a** (0 – 0.176 mM) excited at 450 nm.

6.2 Emission quenching by **2a**

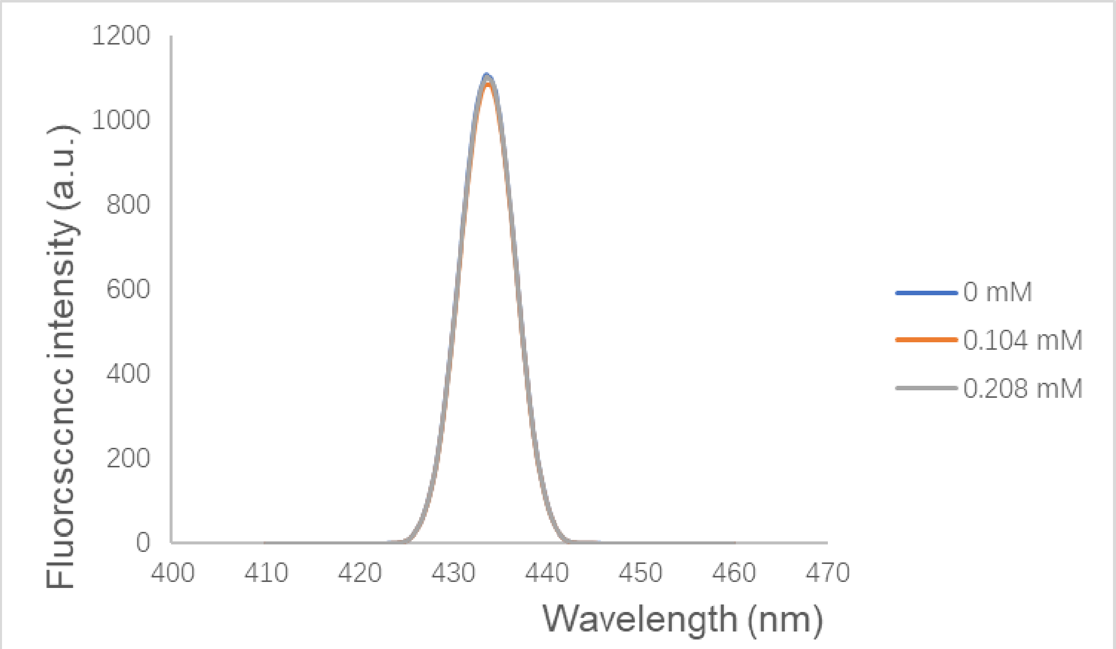

Supplementary Figure 11. Fluorescence titration of 0.5 mM *fac*-Ir(ppy)$_3$ (CH_3CN) with increasing concentration of **2a** (0 – 0.19 mM) excited at 450 nm.

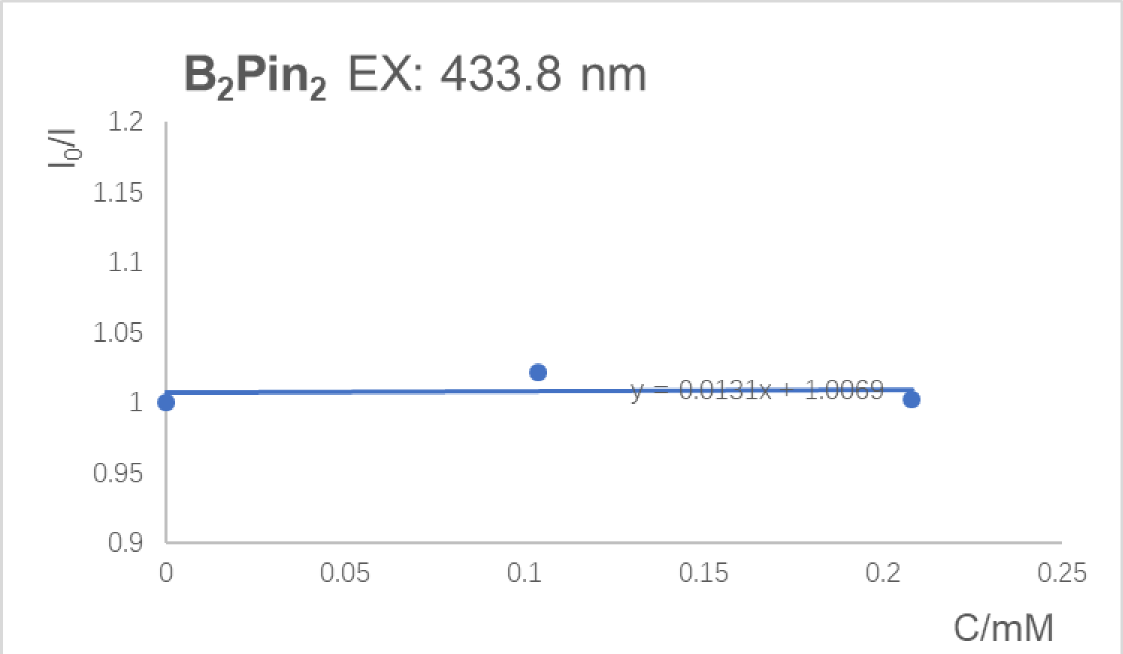

Supplementary Figure 12. Fluorescence titration of 0.5 mM *fac*-Ir(ppy)$_3$ (CH_3CN) with increasing concentration of **2a** (0 – 0.19 mM) excited at 450 nm.

6.3 Emission quenching by **B_2Pin_2**

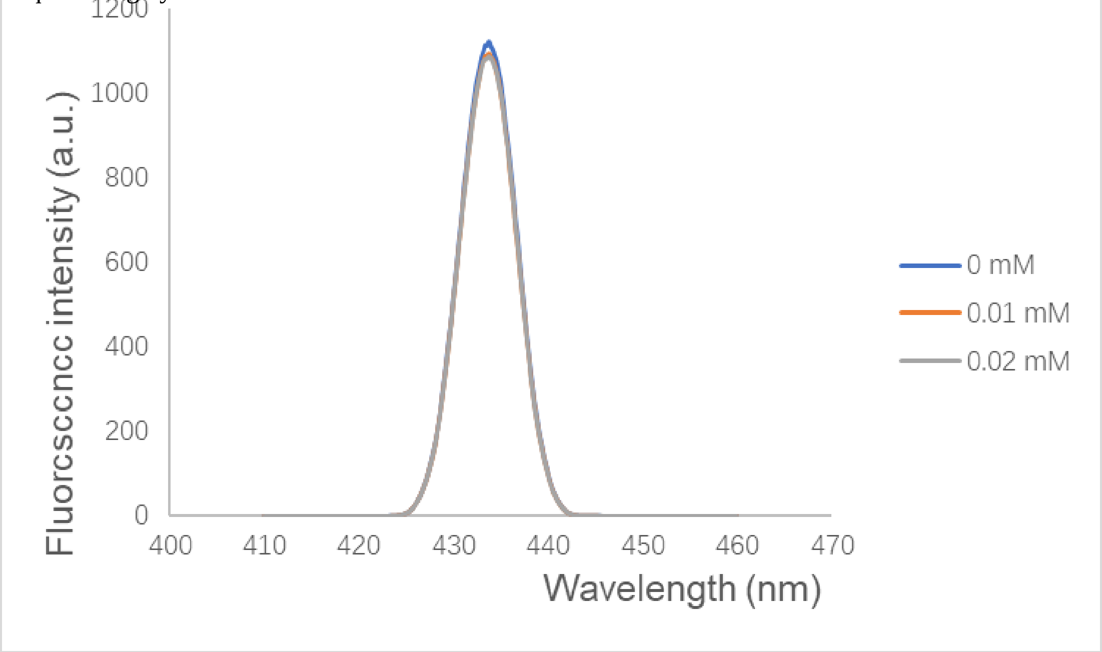

Supplementary Figure 13. Fluorescence titration of 0.5 mM *fac*-Ir(ppy)$_3$ (CH_3CN) with increasing concentration of B_2Pin_2 (0 – 0.208 mM) excited at 450 nm.

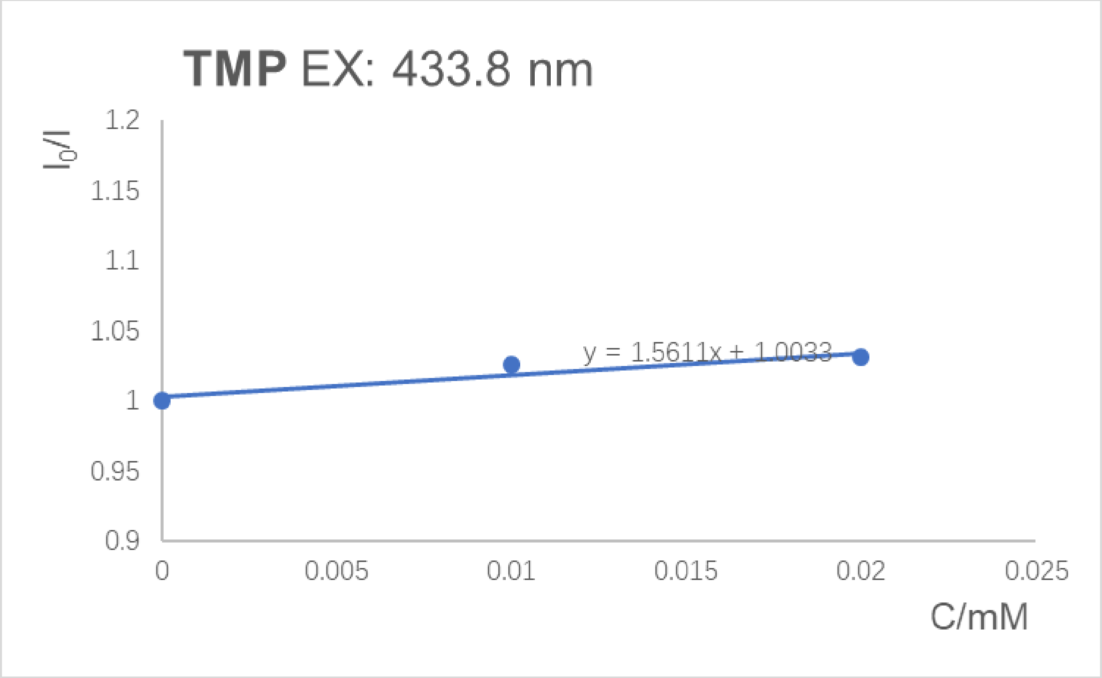

Supplementary Figure 14. Fluorescence titration of 0.5 mM *fac*-Ir(ppy)$_3$ (CH_3CN) with increasing concentration of B_2Pin_2 (0 – 0.208 mM) excited at 450 nm.

6.4 Emission quenching by **TMP**

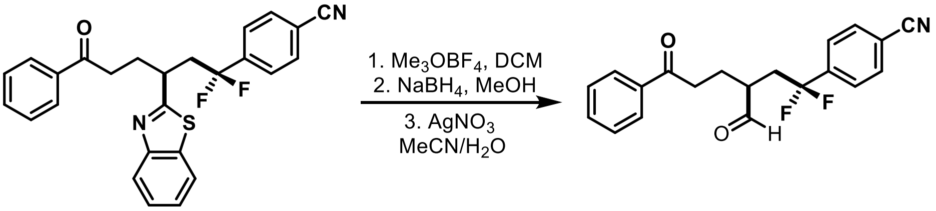

3a **9**

Supplementary Figure 15. Fluorescence titration of 0.5 mM *fac*-Ir(ppy)$_3$ (CH_3CN) with increasing concentration of TMP (0 – 0.02 mM) excited at 450 nm.

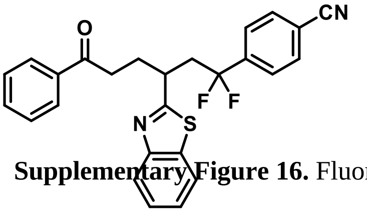

Supplementary Figure 16. Fluorescence titration of 0.5 mM *fac*-Ir(ppy)$_3$ (CH_3CN) with increasing concentration of TMP (0 – 0.02 mM) excited at 450 nm.

7 Product derivation

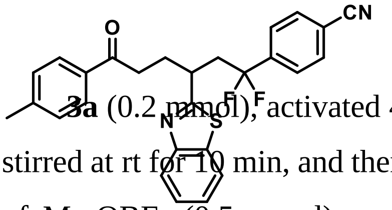

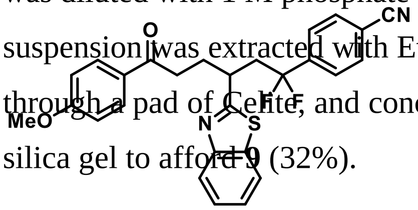

3a (0.2 mmol), activated 4 Å molecular sieves powders (300 mg), and anhydrous DCM (2 mL) was stirred at rt for 10 min, and then Me_3OBF_4 (0.5 mmol) was added. After stirred at rt for 2 h, another batch of Me_3OBF_4 (0.5 mmol) was added to the suspension, which was continued to react until **3a** was consumed as determined by TLC. The reaction was concentrated without filtering off the molecular sieves to give the crude N-methylbenzothiazolium salt. The residue was redissolve in MeOH (2 mL), which was then cooled to 0°C and added $NaBH_4$ (0.3 mmol). Another batch of $NaBH_4$ (0.2 mmol) was added to the reaction until the starting material had been consumed as determined by TLC. The mixture was diluted with acetone, filtered through a pad of Celite, and concentrated to give the crude benzothiazolines. To a vigorously stirred solution of the crude benzothiazolines in CH_2Cl_2 (0.6 mL) and CH_3CN (3 mL) were added H_2O (0.36 mL) followed by $AgNO_3$ (0.6 mmol). The mixture was stirred at rt (monitored by TLC), and then diluted with 1 M phosphate buffer at pH 7 (0.2 mL). After stirred for 15 min, the reaction mixture was diluted with 1 M phosphate buffer at pH 7 (5 mL) and partially concentrated to remove CH_3CN. The suspension was extracted with EtOAc, and the combined organic layers were dried over Na_2SO_4, filtered through a pad of Celite, and concentrated. The residue was purified by flash column chromatography on silica gel to afford 9 (32%).

8. NMR Spectra

4-(3-(benzo[*d*]thiazol-2-yl)-1,1-difluoro-6-oxo-6-phenylhexyl)benzonitrile (**3a**)

66%, white solid. ^{1}H NMR (400 MHz, Chloroform-*d*) δ 8.00 – 7.76 (m, 4H), 7.64 – 7.36 (m, 9H), 3.76-3.65 m, 1H), 3.12 – 2.88 (m, 3H), 2.78 – 2.61 (m, 1H), 2.45 – 2.28 (m, 2H). ^{13}C NMR (100 MHz, Chloroform-*d*) δ 198.8, 173.2, 152.6, 141.1 (t, *J* = 26.4 Hz), 136.6, 134.5, 133.2, 132.1, 128.6, 128.0, 126.3, 126.1, 126.0, 125.3, 122.7, 121.7, 113.8, 44.5 (t, *J* = 27.1 Hz), 38.4 (t, *J* = 3.0 Hz), 30.8. ^{19}F NMR

(376 MHz, Chloroform-*d*) 91.70, 92.36, 97.51, 98.71. FT-IR: *v* (cm^{-1}) 3747, 3660, 3465, 3079, 2934, 2196, 2127, 1758, 1684, 1377, 1244, 1158, 751. HRMS [ESI] calcd for $C_{26}H_{20}F_2N_2OSNa$ $[M+Na]^+$ 469.1157, found 469.1159

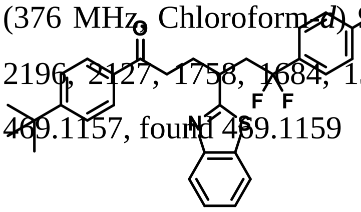

4-(3-(benzo[*d*]thiazol-2-yl)-1,1-difluoro-6-oxo-6-(p-tolyl)hexyl)benzonitrile (**3b**)

42%, colorless oil. ^{1}H NMR (400 MHz, Chloroform-*d*) δ 7.91 (d, *J* = 8.1 Hz, 1H), 7.81 (d, *J* = 7.9 Hz, 1H), 7.74 (d, *J* = 8.2 Hz, 2H), 7.58 – 7.37 (m, 6H), 7.19 (d, *J* = 8.0 Hz, 2H), 3.66 – 3.57 (m, 1H), 3.11 – 2.82 (m, 3H), 2.73 – 2.59 (m, 1H), 2.39 – 2.27 (m, 5H). ^{13}C NMR (100 MHz, Chloroform-*d*) δ 198.4, 173.2, 152.8, 144.1, 141.1, 134.5, 132.1, 129.3, 128.1, 126.2, 125.3, 122.8, 121.7, 44.5, 38.5, 35.3, 30.9, 21.7. ^{19}F NMR (376 MHz, Chloroform-*d*) 91.78, 92.44, 97.48, 98.14. FT-IR: *v* (cm^{-1}) 3330, 2916, 2201, 2129, 2014, 1681, 1598, 1450, 1303, 1140, 759. HRMS [ESI] calcd for $C_{27}H_{22}F_2N_2OSNa$ $[M+Na]^+$ 483.1313, found 483.1287.

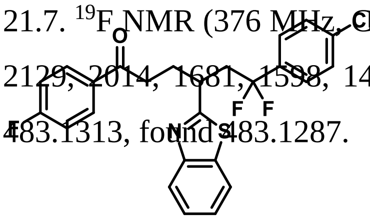

4-(3-(benzo[*d*]thiazol-2-yl)-1,1-difluoro-6-(4-methoxyphenyl)-6-oxohexyl)benzonitrile (**3c**)

59%, colorless oil. ^{1}H NMR (400 MHz, Chloroform-*d*) δ 7.83 (d, *J* = 8.0 Hz, 1H), 7.74 (d, *J* = 7.5 Hz, 1H), 7.66 (d, *J* = 8.2 Hz, 2H), 7.47 (q, *J* = 8.5 Hz, 4H), 7.42 – 7.38 (m, 1H), 7.32 – 7.28 (m, 1H), 7.12 (d, *J* = 8.0 Hz, 2H), 3.60 – 3.49(m, 1H), 3.01 – 2.81 (m, 3H), 2.66 – 2.52 (m, 1H), 2.32 – 2.16 (m, 5H). ^{13}C NMR (100 MHz, Chloroform-*d*) δ 198.4, 173.1, 152.8, 144.0, 141.1 (t, *J* = 27.2 Hz), 134.1, 132.1, 129.3, 128.1, 126.2, 126.0, 125.3, 122.8, 121.6, 117.9, 113.8, 44.5 (t, *J* = 26.7 Hz), 38.5 (t, *J* = 2.9 Hz), 35.3, 30.9, 21.6. ^{19}F NMR (376 MHz, Chloroform-*d*) 91.70, 92.37, 97.44, 98.11. FT-IR: *v* (cm^{-1})3746, 3622, 3462, 2980, 2421, 1681, 1611, 1456, 1315, 1173, 1067, 753. HRMS [ESI] calcd for $C_{27}H_{22}F_2N_2OSNa$ $[M+Na]^+$ 499.1262, found 499.1272.

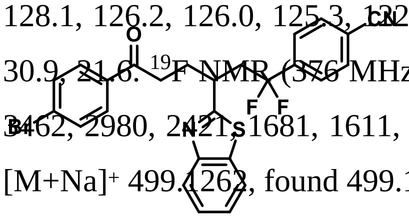

4-(3-(benzo[*d*]thiazol-2-yl)-6-(4-(tert-butyl)phenyl)-1,1-difluoro-6-oxohexyl)benzonitrile (**3d**)

66%, colorless oil. ^{1}H NMR (400 MHz, Chloroform-*d*) δ 7.91 (d, *J* = 8.1 Hz, 1H), 7.86 – 7.75 (m, 3H), 7.57 – 7.36 (m, 8H), 3.68 – 3.58 (m, 1H), 3.09 – 2.86 (m, 3H), 2.73-2.60 (m, 1H), 2.41 – 2.24 (m, 2H), 1.31 (s, 9H). ^{13}C NMR (100 MHz, Chloroform-*d*) δ 198.4, 173.2, 157.0, 152.7, 134.0, 132.1, 127.9, 126.2, 126.0 (t, *J* = 60.8 Hz) 125.5, 125.3, 122.7, 121.6, 44.5 (t, *J* = 27.5 Hz), 38.5, 35.3, 35.1, 31.1, 30.9. ^{19}F NMR (376 MHz, Chloroform-*d*) 91.79, 92.45, 97.49, 98.15. FT-IR: *v* (cm^{-1})3087, 2369, 2323, 2136, 1937, 1686, 1601, 1380, 1240, 1065, 756. HRMS [ESI] calcd for $C_{30}H_{28}F_2N_2OSNa$ $[M+Na]^+$ 525.1783, found 525.1778.

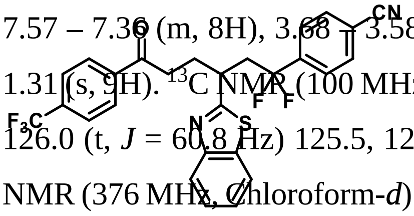

4-(3-(benzo[*d*]thiazol-2-yl)-1,1-difluoro-6-(4-fluorophenyl)-6-oxohexyl)benzonitrile (**3e**)

61%, colorless oil.^{1}H NMR (400 MHz, Chloroform-*d*) δ 7.97 – 7.81 (m, 4H), 7.62 – 7.38 (m, 6H), 7.09 (t, *J* = 8.6 Hz, 2H), 3.74 – 3.60 (m, 1H), 3.10 – 2.89 (m, 3H), 2.75 – 2.61 (m, 1H), 2.44 – 2.27 (m, 2H). ^{13}C NMR (100 MHz, Chloroform-*d*) δ 197.1, 173.0, 167.1, 164.5, 152.6, 141.0 (t, *J* = 26.6 Hz), 134.5, 133.0, 132.1, 130.7, 126.1, 126.0 (t, *J* = 6.2 Hz) , 125.4, 122.7, 121.7, 117.8, 115.8, 115.6, 113.9, 44.5 (t, *J* = 26.5 Hz), 38.4, 35.3, 30.8. ^{19}F NMR (376 MHz, Chloroform-*d*) 91.73, 92.39, 97.62, 98.29, 104.95. FT-IR: *v* (cm^{-1}) 3073, 2321, 2173, 2058, 1684, 1597, 1507, 1235, 1160, 763. HRMS [ESI] calcd for $C_{26}H_{19}F_3N_2OSNa$ $[M+Na]^+$ 497.1062, found 487.1032.

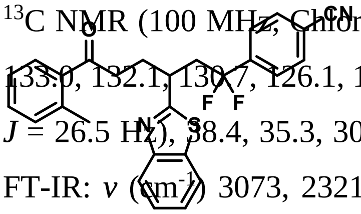

4-(3-(benzo[*d*]thiazol-2-yl)-6-(4-bromophenyl)-1,1-difluoro-6-oxohexyl)benzonitrile (**3f**)

30%, colorless oil. ^{1}H NMR (400 MHz, Chloroform-*d*) δ 7.98 – 7.88 (m, 1H), 7.87 – 7.68 (m, 3H), 7.60 – 7.37 (m, 8H), 3.73 – 3.55 (m, 1H), 3.15 – 2.83 (m, 3H), 2.74 – 2.60 (m, 1H), 2.43 – 2.25 (m, 2H). ^{13}C NMR (100 MHz, Chloroform-*d*) δ 198.8, 197.7, 173.0, 152.6, 135.2, 134.4, 132.2, 131.9, 129.5, 128.6, 128.4, 128.0, 126.4, 126.0 (t, *J* = 6.4 Hz), 125.4, 122.7, 121.7, 113.8, 44.5 (t, *J* = 30.6 Hz), 38.3, 35.4,

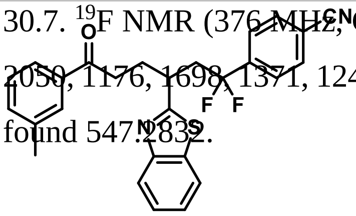

30.7. ^{19}F NMR (376 MHz, Chloroform-*d*) 91.92, 92.58, 97.60, 98.26. FT-IR: *v* (cm^{-1}) 3084, 2323, 2149, 2050, 1176, 1698, 1371, 1245, 1140, 753. HRMS [ESI] calcd for $C_{26}H_{19}BrF_2N_2OSNa$ $[M+Na]^+$ 547.0262, found 547.2832.

4-(3-(benzo[*d*]thiazol-2-yl)-1,1-difluoro-6-oxo-6-(4-(trifluoromethyl)phenyl)hexyl)benzonitrile (**3g**)

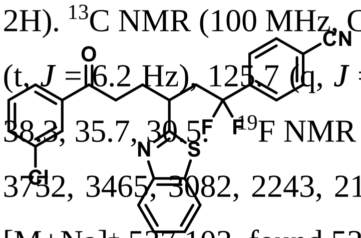

51%, colorless oil. ^{1}H NMR (400 MHz, Chloroform-*d*) δ 7.93 (t, *J* = 8.3 Hz, 3H), 7.82 (d, *J* = 7.9 Hz, 1H), 7.69 – 7.35 (m, 8H), 3.74 – 3.59 (m, 1H), 3.14 – 2.87 (m, 3H), 2.75 – 2.60 (m, 1H), 2.46 – 2.28 (m, 2H). ^{13}C NMR (100 MHz, Chloroform-*d*) δ 197.8, 172.9, 152.2, 141.0, 139.1, 132.2, 128.3, 126.4, 126.0 (t, *J* = 6.2 Hz), 125.7 (q, *J* = 3.6 Hz) , 125.5, 122.7, 121.7, 121.3, 117.9, 113.9, 44.5 (t, *J* = 27.1 Hz), 38.3, 35.7, 30.5. ^{19}F NMR (376 MHz, Chloroform-*d*) 63.17. 91.78, 92.45, 97.76, 98.42. FT-IR: *v* (cm^{-1}) 3732, 3465, 3082, 2243, 2126, 1693, 1320, 1140,1128, 760. HRMS [ESI] calcd for $C_{27}H_{19}F_5N_2OSNa$ $[M+Na]^+$ 537.103, found 537.1007.

4-(3-(benzo[*d*]thiazol-2-yl)-1,1-difluoro-6-oxo-6-(o-tolyl)hexyl)benzonitrile (**3h**)

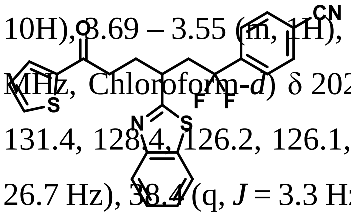

54%, coloress oil. ^{1}H NMR (400 MHz, Chloroform-*d*) δ 7.88 (dd, *J* = 35.9, 7.9 Hz, 2H), 7.72 – 7.16 (m, 10H), 3.69 – 3.55 (m, 1H), 3.10 – 2.85 (m, 3H), 2.72 – 2.61 (m, 1H), 2.59 – 2.18 (m, 5H). ^{13}C NMR (100 MHz, Chloroform-*d*) δ 202.6, 173.0, 152.8, 141.1 (t, *J* = 26.9 Hz), 138.1, 137.5, 134.5, 132.1, 132.0, 131.4, 128.4, 126.2, 126.1, 126.0 (t, *J* = 6.3 Hz) , 125.7, 125.3, 122.8, 121.6, 117.9, 113.8, 44.5 (q, *J* = 26.7 Hz), 38.4 (q, *J* = 3.3 Hz), 38.2, 30.9, 21.3. ^{19}F NMR (376 MHz, Chloroform-*d*) 91.70, 92.36, 97.52, 98.19. FT-IR: *v* (cm^{-1}) 3732, 3607, 3082, 2456, 2240, 2018, 1681, 1251, 1168, 1055, 754. HRMS [ESI] calcd for $C_{27}H_{22}F_2N_2OSNa$ $[M+Na]^+$ 483.1313, found 483.1298.

4-(3-(benzo[*d*]thiazol-2-yl)-1,1-difluoro-6-oxo-6-(m-tolyl)hexyl)benzonitrile (**3i**)

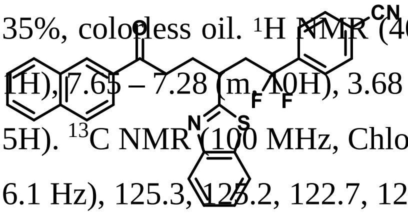

35%, colorless oil. ^{1}H NMR (400 MHz, Chloroform-*d*) δ 7.92 (d, *J* = 8.1 Hz, 1H), 7.82 (d, *J* = 8.0 Hz, 1H), 7.65 – 7.28 (m, 10H), 3.68 – 3.57 (m, 1H), 3.14 – 2.82 (m, 3H), 2.73 – 2.61 (m, 1H), 2.40 – 2.27 (m, 5H). ^{13}C NMR (100 MHz, Chloroform-d) δ 199.0, 138.4, 136.6, 134.0, 132.1, 128.5, 126.3, 126.0 (t, *J* = 6.1 Hz), 125.3, 12[illegible].2, 122.7, 121.7, 77.3, 44.5, 38.4, 35.4, 30.9, 21.3. FT-IR: *v* (cm^{-1}) 3080, 2513, 2316, 2141, 2011, 1650, 1490, 1290, 1140, 1053, 759. HRMS [ESI] calcd for $C_{27}H_{22}F_2N_2OSH$ [M+H]$^+$ 461.1494, found 461.1469.

4-(3-(benzo[*d*]thiazol-2-yl)-6-(3-chlorophenyl)-1,1-difluoro-6-oxohexyl)benzonitrile (**3j**)

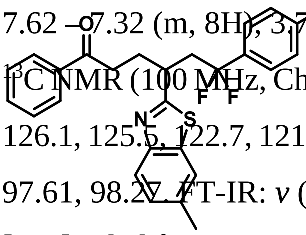

44%, colorless oil.^{1}H NMR (400 MHz, Chloroform-*d*) δ 8.02 – 7.80 (m, 3H), 7.71 (d, *J* = 8.9 Hz, 1H), 7.62 – 7.32 (m, 8H), 3.72 – 3.57 (m, 1H), 3.18 – 2.83 (m, 3H), 2.75 – 2.60 (m, 1H), 2.44 – 2.28 (m, 2H). ^{13}C NMR (100 MHz, Chloroform-*d*) δ 197.5, 173.1, 152.4, 138.0, 135.0, 133.2, 132.2, 130.0, 128.1, 126.4, 126.1, 125.5, 122.7, 121.7, 113.9, 44.8, 38.3, 35.5, 30.6. ^{19}F NMR (376 MHz, Chloroform-*d*) 91.78, 92.44, 97.61, 98.27. FT-IR: *v* (cm^{-1}) 3747, 2926, 2856, 2381, 2324, 2136, 1690, 1314, 1170, 1068, 763. HRMS [ESI] calcd for $C_{26}H_{19}ClF_2N_2OSNa$ [M+Na]$^+$ 503.0767, found 503.0763.

4-(3-(benzo[*d*]thiazol-2-yl)-1,1-difluoro-6-oxo-6-(thiophen-2-yl)hexyl)benzonitrile (**3k**)

38%, colorless oil. ^{1}H NMR (400 MHz, Chloroform-*d*) δ 7.93 – 7.79 (m, 2H), 7.63 – 7.35 (m, 8H), 7.12 – 6.99 (m, 1H), 3.68 – 3.55 (m, 1H), 3.06 – 2.85 (m, 3H), 2.72 – 2.58 (m, 1H), 2.43 – 2.25 (m, 2H). ^{13}C NMR (100 MHz, Chloroform-*d*) δ 191.7, 172.9, 152.8, 143.8, 141.0, 134.6, 133.8, 132.1, 131.9, 128.1, 126.3, 126.1, 126.0 (t, *J* = 6.4 Hz), 125.3, 122.8, 121.7, 117.9, 113.8, 44.4 (t, *J* = 26.9 Hz), 38.4 (t, *J* = 3.2 Hz), 36.1, 30.9. ^{19}F NMR (376 MHz, Chloroform-*d*) 91.93, 92.60, 97.42, 98.09. FT-IR: *v* (cm^{-1}) 3083,

2317, 2137, 1933, 1658, 1514, 1420, 1233, 1140, 1054, 724. HRMS [ESI] calcd for $C_{24}H_{18}F_2N_2OS_2Na$ $[M+Na]^+$ 475.0721, found 475.3212.

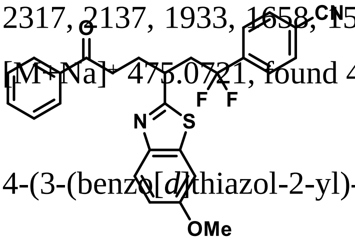

4-(3-(benzo[*d*]thiazol-2-yl)-1,1-difluoro-6-(naphthalen-2-yl)-6-oxohexyl)benzonitrile (**3l**)

39%, colorless oil. ^{1}H NMR (400 MHz, Chloroform-*d*) δ 8.16 – 7.64 (m, 5H), 7.60 – 7.34 (m, 10H), 3.72 – 3.56 (m, 1H), 3.13 – 2.82 (m, 3H), 2.73 – 2.59 (m, 1H), 2.42 – 2.24 (m, 2H). ^{13}C NMR (100 MHz, Chloroform-*d*) δ 197.6, 173.0, 152.6, 141.0 (t, *J* = 26.7 Hz), 139.7, 139.3, 134.8, 134.4, 132.2, 129.4, 129.0, 128.9, 128.1, 126.4, 126.0 (t, *J* = 6.3 Hz), 125.9, 125.4, 123.1, 122.7, 121.9, 121.7, 117.9, 113.8, 44.5 (t, *J* = 26.8 Hz), 38.3, 35.4, 30.7. ^{19}F NMR (376 MHz, Chloroform-*d*) 91.88, 92.54, 97.61, 98.27. FT-IR: *v* (cm^{-1}) 3750, 3610, 3463, 2923, 2374, 1683, 1586, 1487, 1304, 1254, 1167, 760. HRMS [ESI] calcd for $C_{30}H_{22}F_2N_2OSNa$ $[M+Na]^+$ 519.1313, found 519.0464.

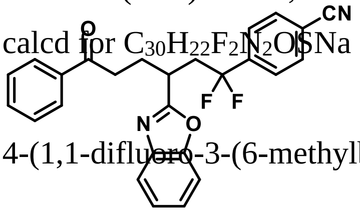

4-(1,1-difluoro-3-(6-methylbenzo[d]thiazol-2-yl)-6-oxo-6-phenylhexyl)benzonitrile (**3m**)

71%, colorless oil. ^{1}H NMR (400 MHz, Chloroform-*d*) δ 7.87 – 7.74 (m, 3H), 7.63 – 7.49 (m, 6H), 7.39 (t, *J* = 7.7 Hz, 2H), 7.29 – 7.26 (m, 1H), 3.65 – 3.52 (m, 1H), 3.07 – 2.87 (m, 3H), 2.72 – 2.59 (m, 1H), 2.50 – 2.23 (m, 5H). ^{13}C NMR (100 MHz, Chloroform-*d*) δ 198.8, 171.9, 150.9, 141.1 (t, *J* = 26.8 Hz), 136.6, 135.4, 134.7, 133.2, 132.1, 128.6, 127.8, 126.0 (t, *J* = 6.3 Hz), 122.3, 121.4, 117.9, 113.7, 44.5 (t, *J* = 26.8 Hz), 38.4, 35.4, 30.8, 21.5. ^{19}F NMR (376 MHz, Chloroform-*d*) 91.42, 92.08, 97.67, 98.34. FT-IR: *v* (cm^{-1}) 3086, 2507, 2317, 2140, 1943, 1684, 1453, 1248, 1173, 1050, 741. HRMS [ESI] calcd for $C_{27}H_{22}F_2N_2OSNa$ $[M+Na]^+$483.1313, found 483.1304.

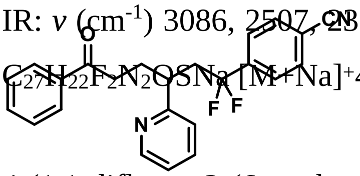

4-(1,1-difluoro-3-(6-methoxybenzo[d]thiazol-2-yl)-6-oxo-6-phenylhexyl)benzonitrile (**3n**)

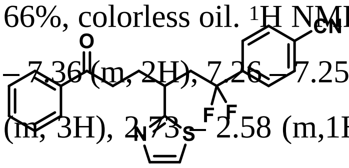

66%, colorless oil. ^{1}H NMR (400 MHz, Chloroform-*d*) δ 7.86 – 7.72 (m, 3H), 7.61 – 7.50 (m, 5H), 7.46 – 7.36 (m, 2H), 7.26 – 7.25 (1H), 7.13 – 7.03 (1H), 3.94 – 3.84 (m, 3H), 3.64 – 3.55 (m, 1H), 3.10 – 2.87 (m, 3H), 2.73 – 2.58 (m,1H), 2.42 – 2.25 (m,2H). ^{13}C NMR (100 MHz, Chloroform-*d*) δ 198.8, 170.6, 157.8, 141.1 (t, *J* = 27.0 Hz), 136.5, 135.6, 133.2, 132.1, 128.6, 128.0, 126.0 (t, *J* = 6.0 Hz), 123.1, 117.9, 115.6, 113.7, 55.9, 44.5 (t, *J* = 26.0 Hz), 38.2, 35.4, 30.7. ^{19}F NMR (376 MHz, Chloroform-*d*) 91.95, 92.61, 97.41, 98.08. FT-IR: *v* (cm^{-1}) 3077, 2320, 2137, 1998, 1681, 1453, 1224, 1051, 830, 740. HRMS [ESI] calcd for $C_{27}H_{22}F_2N_2O_2SNa$ [M+Na]$^+$499.1262, found 499.1247.

4-(3-(benzo[*d*]oxazol-2-yl)-1,1-difluoro-6-oxo-6-phenylhexyl)benzonitrile (**3o**)

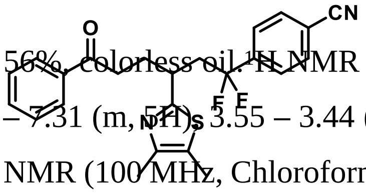

56%, colorless oil. ^{1}H NMR (400 MHz, Chloroform-*d*) δ 7.91 – 7.82 (m, 2H), 7.64 – 7.51 (m, 6H), 7.45 – 7.31 (m, 5H), 3.55 – 3.44 (m, 1H), 3.08 – 2.91 (m, 3H), 2.73 – 2.60 (m, 1H), 2.41 – 2.28 (m, 2H). ^{13}C NMR (100 MHz, Chloroform-*d*) δ 198.5, 166.7, 150.3, 140.6 (t, *J* = 32.5 Hz), 136.5, 133.2, 132.0, 128.6, 128.0, 126.0 (t, *J* = 6.4 Hz), 125.9, 125.9, 125.1, 124.6, 119.7, 113.7, 110.5, 76.7, 42.6 (t, *J* = 27.3 Hz), 35.4, 34.0 (t, *J* = 4.0 Hz), 28.5.^{19}F NMR (376 MHz, Chloroform-*d*) 91.74, 92.41, 98.49, 99.16. FT-IR: *v* (cm^{-1}) 2996, 2760, 2167, 1683, 1452, 1220, 1248, 1167, 745. HRMS [ESI] calcd for $C_{24}H_{18}F_2N_2OS_2Na$ [M+Na]$^+$ 453.1385, found 453.1373.

4-(1,1-difluoro-6-oxo-6-phenyl-3-(pyridin-2-yl)hexyl)benzonitrile (**3p**)

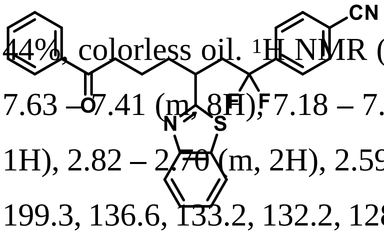

44%, colorless oil. ^{1}H NMR (400 MHz, Chloroform-*d*) δ 8.51 (d, *J* = 4.6 Hz, 1H), 7.87 – 7.80 (m, 2H), 7.63 – 7.41 (m, 8H), 7.18 – 7.09 (m, 1H), 7.01 (d, *J* = 7.5 Hz, 1H), 3.24 – 3.15 (m, 1H), 3.06 – 2.91 (m, 1H), 2.82 – 2.70 (m, 2H), 2.59 – 2.46 (m, 1H), 2.26-2.17 (m, 2H). ^{13}C NMR (100 MHz, Chloroform-*d*) δ 199.3, 136.6, 133.2, 132.2, 128.6, 127.9, 126.0, 122.1, 118.1, 113.7, 43.6 (t, *J* = 26.3 Hz), 40.8, 35.6, 30.6.

^{19}F NMR (376 MHz, Chloroform-*d*) 91.86, 92.50, 97.11, 97.78. FT-IR: *v* (cm^{-1}) 2916, 2850, 2323, 2129, 2006, 1674, 1466, 1151, 745. HRMS [ESI] calcd for $C_{24}H_{20}F_2N_2OSNa$ [M+Na]$^+$ 413.1436, found 413.1431.

4-(1,1-difluoro-6-oxo-6-phenyl-3-(thiazol-2-yl)hexyl)benzonitrile (**3q**)

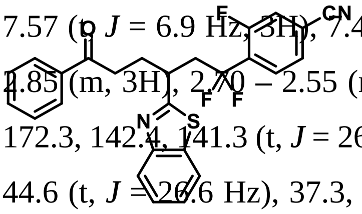

37%, colorless oil. ^{1}H NMR (400 MHz, Chloroform-*d*) δ 7.87 (d, *J* = 7.4 Hz, 2H), 7.72 – 7.64 (m, 3H), 7.57 (t, *J* = 6.9 Hz, 3H), 7.45 (t, *J* = 7.6 Hz, 2H), 7.21 (d, *J* = 3.1 Hz, 1H), 3.66 – 3.57 (m, 1H), 3.06 – 2.85 (m, 3H), 2.70 – 2.55 (m, 1H), 2.38 – 2.20 (m, 2H). ^{13}C NMR (100 MHz, Chloroform-*d*) δ 198.9, 172.3, 142.4, 141.3 (t, *J* = 26.8 Hz), 136.6, 133.2, 132.3, 128.6, 127.9, 126.0 (t, *J* = 6.0 Hz), 118.5, 113.9, 44.6 (t, *J* = 26.6 Hz), 37.3, 35.3, 31.2. ^{19}F NMR (376 MHz, Chloroform-*d*) 92.61, 93.28, 96.97, 97.63. FT-IR: *v* (cm^{-1}) 3087, 2370, 2321, 2136, 2013, 1683, 1448, 1317, 1248, 1173, 1044, 741. HRMS [ESI] calcd for $C_{22}H_{18}F_2N_2OSNa$ [M+Na]$^+$ 419.1, found 419.0985.

4-(3-(4,5-dimethylthiazol-2-yl)-1,1-difluoro-6-oxo-6-phenylhexyl)benzonitrile (**3r**)

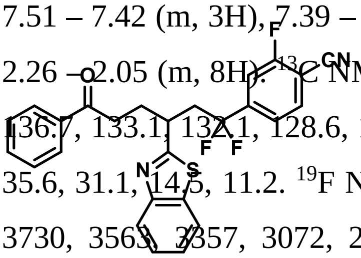

40%, colorless oil. ^{1}H NMR (400 MHz, Chloroform-*d*) δ 7.83 – 7.76 (m, 2H), 7.57 (d, *J* = 8.4 Hz, 2H), 7.51 – 7.42 (m, 3H), 7.39 – 7.33 (m, 2H), 3.33-3.25 (m, 1H), 2.90 – 2.70 (m, 3H), 2.56 – 2.42 (m, 1H), 2.26 – 2.05 (m, 8H). ^{13}C NMR (100 MHz, Chloroform-*d*) δ 199.0, 167.3, 147.5, 141.3 (t, *J* = 26.8 Hz), 136.7, 133.1, 132.1, 128.6, 128.0, 126.1, 125.6, 118.0, 113.6, 44.7 (t, *J* = 26.6 Hz), 37.5 (t, *J* = 3.1 Hz), 35.6, 31.1, 14.5, 11.2. ^{19}F NMR (376 MHz, Chloroform-*d*) 91.01, 91.68, 97.84, 98.50. FT-IR: *v* (cm^{-1}) 3730, 3563, 3357, 3072, 2321, 2129, 2007, 1314, 1250, 1213, 1063, 747. HRMS [ESI] calcd for $C_{24}H_{22}F_2N_2OSNa$ [M+Na]$^+$ 447.1313, found 447.1295.

4-(3-(benzo[*d*]thiazol-2-yl)-1,1-difluoro-7-oxo-7-phenylheptyl)benzonitrile (**3t**)

59%, colorless oil. ^{1}H NMR (400 MHz, Chloroform-*d*) δ 7.92 (t, *J* = 7.8 Hz, 3H), 7.83 (d, *J* = 7.9 Hz, 1H), 7.60 – 7.37 (m, 9H), 3.59 – 3.50 (m, 1H), 3.07 – 2.90 (m, *J* = 6.8 Hz, 3H), 2.73 – 2.59 (m, 1H), 2.04 – 1.94 (m, 2H), 1.84 – 1.66 (m, 4H). ^{13}C NMR (100 MHz, Chloroform-*d*) δ 199.4, 173.8, 136.8, 133.1, 132.1, 128.6, 128.0, 126.3, 126.1, 126.0 (t, *J* = 6.2 Hz), 125.3, 122.6, 121.6, 39.1, 39.0, 37.8, 36.2, 21.4. ^{19}F NMR (376 MHz, Chloroform-*d*) 91.83, 92.49, 97.65, 98.31. FT-IR: *ν* (cm^{-1}) 3743, 3612, 3469, 2919, 2441, 2269, 1754, 1678, 1361, 1313, 1241, 1108, 760.HRMS [ESI] calcd for $C_{27}H_{22}F_2N_2OSNa$ [M+Na]$^+$ 483.1313, found 483.1297.

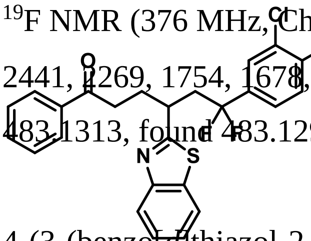

4-(3-(benzo[*d*]thiazol-2-yl)-1,1-difluoro-6-oxo-6-phenylhexyl)-3-fluorobenzonitrile (**4a**)

77%, colorless oil. ^{1}H NMR (400 MHz, Chloroform-*d*) δ 7.93 – 7.77 (m, 4H), 7.56 – 7.37 (m, 6H), 7.28 (d, *J* = 7.9 Hz, 1H), 7.21 (d, *J* = 10.0 Hz, 1H), 3.69 – 3.56 (m, 1H), 3.25 – 2.76 (m, 4H), 2.44 – 2.26 (m, 2H). ^{13}C NMR (100 MHz, Chloroform-*d*) δ 198.7, 172.7, 152.7, 136.6, 134.5, 133.2, 128.6, 128.0, 128.0, 127.8, 127.7, 126.3, 125.4, 122.7, 121.6, 119.9, 119.6, 115.4, 43.0 (t, *J* = 26.0 Hz), 38.5 (t, *J* = 2.0 Hz), 35.4, 30.8. ^{19}F NMR (376 MHz, Chloroform-*d*) 88.62, 89.30, 96.89, 97.55, 110.28, 114.84. FT-IR: *ν* (cm^{-1}) 3094, 2590, 2317, 2134, 2017, 1687, 1311, 1254, 1064, 754. HRMS [ESI] calcd for $C_{26}H_{19}F_3N_2OSNa$ [M+Na]$^+$ 487.1062, found 487.1043.

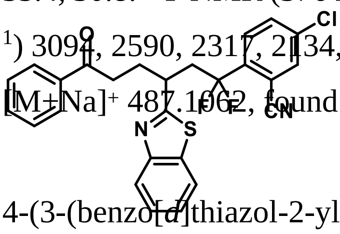

4-(3-(benzo[*d*]thiazol-2-yl)-1,1-difluoro-6-oxo-6-phenylhexyl)-2-fluorobenzonitrile (**4b**)

82%, colorless oil. ^{1}H NMR (400 MHz, Chloroform-*d*) δ 7.91 – 7.80 (m, 4H), 7.54 – 7.46 (m, 3H), 7.39 (t, *J* = 7.7 Hz, 3H), 7.32 – 7.25 (m, 2H), 3.68 – 3.58 (m, 1H), 3.07 – 2.93 (m, 3H), 2.73 – 2.61 (m, 1H), 2.40 – 2.26 (m, 2H). ^{13}C NMR (100 MHz, $CDCl_3$) δ 198.7, 172.7, 164.0, 161.4, 152.8, 144.1, 144.0, 143.8 (t, *J* = 27.7 Hz), 143.6, 143.5, 136.5, 134.5, 133.6, 133.3, 128.6, 128.0, 126.4, 125.4, 122.8, 121.7, 114.1, 114.0, 113.9, 113.8, 113.8, 113.7, 113.0, 102.8, 102.7, 44.3 (t, *J* = 26.6 Hz), 38.3 (t, *J* = 1.3 Hz), 35.3, 30.8. ^{19}F NMR (376 MHz, Chloroform-*d*) 90.86, 91.52, 97.82, 98.49, 104.85. FT-IR: *ν* (cm^{-1}) 3082, 2369,

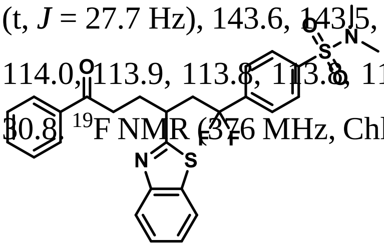

2129, 2013, 1937, 1681, 1507, 1433, 1064, 751. HRMS [ESI] calcd for $C_{26}H_{19}F_3N_2OSNa$ $[M+Na]^+$ 487.1062, found 487.1034.

4-(3-(benzo[*d*]thiazol-2-yl)-1,1-difluoro-6-oxo-6-phenylhexyl)-2-chlorobenzonitrile (**4c**)

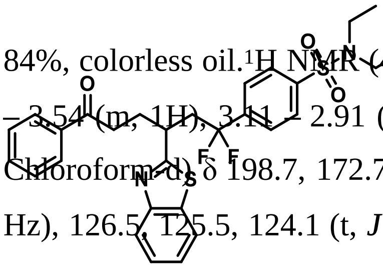

84%, colorless oil. ^{1}H NMR (400 MHz, Chloroform-*d*) δ 7.95 – 7.79 (m, 4H), 7.56 – 7.36 (m, 8H), 3.72 – 3.54 (m, 1H), 3.11 – 2.91 (m, 3H), 2.75 – 2.62 (m, 1H), 2.41 – 2.28 (m, 2H). ^{13}C NMR (100 MHz, Chloroform-*d*) δ 198.7, 172.7, 152.4, 137.1, 136.5, 134.3, 133.8, 133.3, 128.6, 128.0, 127.0 (t, *J* = 6.8 Hz), 126.5, 125.5, 124.1 (t, *J* = 5.6 Hz), 122.7, 121.7, 115.1, 44.3 (t, *J* = 26.5 Hz), 38.3, 35.3, 30.8. ^{19}F NMR (376 MHz, Chloroform-*d*) 90.23, 90.90, 98.68, 99.34. FT-IR: *v* (cm^{-1}) 3079, 2926, 2141, 2056, 1946, 1683, 1237, 1063, 750. HRMS [ESI] calcd for $C_{26}H_{19}ClF_2N_2OSNa$ $[M+Na]^+$503.0767, found 503.0783.

2-(3-(benzo[d]thiazol-2-yl)-1,1-difluoro-6-oxo-6-phenylhexyl)-5-chlorobenzonitrile (**4g**)

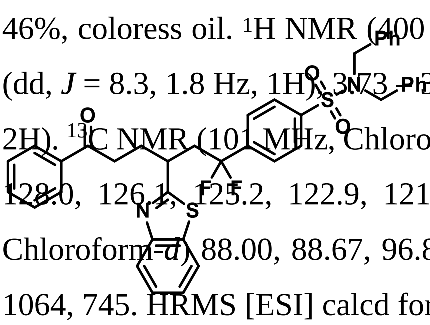

46%, coloress oil. ^{1}H NMR (400 MHz, Chloroform-*d*) δ 7.91 – 7.76 (m, 4H), 7.54 – 7.33 (m, 7H), 7.23 (dd, *J* = 8.3, 1.8 Hz, 1H), 3.73 – 3.62 (m, 1H), 3.33 – 3.17 (m, 1H), 3.06 – 2.80 (m, 3H), 2.44 – 2.28 (m, 2H). ^{13}C NMR (101 MHz, Chloroform-*d*) δ 198.7, 172.5, 139.4, 136.6, 135.1, 134.6, 133.2, 130.2, 128.6, 128.0, 126.1, 125.2, 122.9, 121.6, 116.2, 43.0 (t, *J* = 26.2 Hz), 38.4, 30.8. ^{19}F NMR (376 MHz, Chloroform-*d*) 88.00, 88.67, 96.85, 97.52. FT-IR: *v* (cm^{-1}) 3092, 2569, 2120, 2006, 1689, 1500, 1431, 1064, 745. HRMS [ESI] calcd forC26H19ClF2N2OSNa $[M+Na]^+$ 503.0767, found 503.0671.

4-(3-(benzo[*d*]thiazol-2-yl)-1,1-difluoro-6-oxo-6-phenylhexyl)-N,N-dimethylbenzenesulfonamide (**4h**)

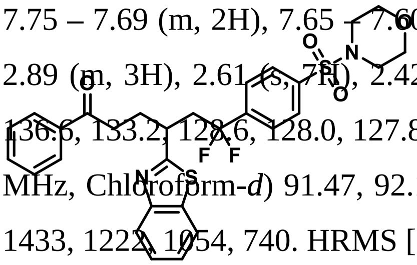

60%, colorless oil.^{1}H NMR (400 MHz, Chloroform-d) δ 7.94 (d, *J* = 8.1 Hz, 1H), 7.87 – 7.79 (m, 3H), 7.75 – 7.69 (m, 2H), 7.65 – 7.60 (d, *J* = 8.4 Hz, 2H), 7.55 – 7.35 (m, 5H), 3.71 – 3.62 (m, 1H), 3.14 – 2.89 (m, 3H), 2.61 (s, 7H), 2.42 – 2.26 (m, 2H). ^{13}C NMR (100 MHz, Chloroform-*d*) δ 198.8, 146.0, 136.6, 133.2, 128.6, 128.0, 127.8, 126.0, 125.9, 125.2, 122.8, 1217, 38.4, 37.8, 35.4, 30.9. ^{19}F NMR (376 MHz, Chloroform-*d*) 91.47, 92.13, 96.84, 97.50. FT-IR: *ν* (cm^{-1}) 3077, 2310, 2323, 2139, 1677, 1618, 1433, 1222, 1054, 740. HRMS [ESI] calcd for $C_{27}H_{26}F_2N_2O_3S_2Na$ [M+Na]$^+$551.1245, found 551.3458.

4-(3-(benzo[*d*]thiazol-2-yl)-1,1-difluoro-6-oxo-6-phenylhexyl)-N,N-diethylbenzenesulfonamide (**4i**)

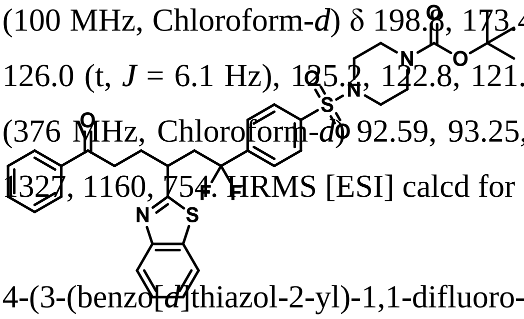

70%, colorless oil.^{1}H NMR (400 MHz, Chloroform-*d*) δ 7.96 (d, *J* = 8.1 Hz, 1H), 7.91 – 7.76 (m, 5H), 7.64 (d, *J* = 8.3 Hz, 2H), 7.56 – 7.37 (m, 5H), 3.74 – 3.65 (m, 1H), 3.22 (t, *J* = 6.6 Hz, 4H), 3.12 – 2.92 (m, 3H), 2.77 – 2.62 (m, 1H), 2.45 – 2.28 (m, 2H), 1.82 – 1.73 (m, 4H), 1.41 – 1.20 (m, 2H). ^{13}C NMR (100 MHz, Chloroform-*d*) δ 198.8, 173.4, 152.7, 138.7, 136.6, 134.5, 133.2, 128.6, 128.0, 127.6, 126.3, 126.0 (t, *J* = 6.1 Hz), 125.2, 122.8, 121.7, 47.9, 44.5 (t, *J* = 27.0 Hz), 38.4, 35.5, 30.8, 25.3. ^{19}F NMR (376 MHz, Chloroform-*d*) 92.59, 93.25, 96.06, 96.72. FT-IR: *ν* (cm^{-1}) 3087, 2321, 2253, 2131, 1691, 1327, 1160, 754. HRMS [ESI] calcd for $C_{29}H_{30}F_2N_2O_3S_2Na$ [M+Na]$^+$579.1558, found 579.1380.

4-(3-(benzo[*d*]thiazol-2-yl)-1,1-difluoro-6-oxo-6-phenylhexyl)-N,N-dibenzylbenzenesulfonamide (**4j**)

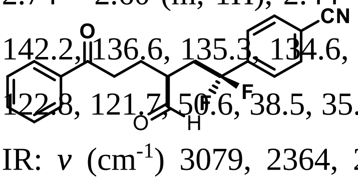

70%, colorless oil.^{1}H NMR (400 MHz, Chloroform-*d*) δ 8.01 – 7.74 (m, 6H), 7.61 – 7.33 (m, 8H), 7.25 (d, *J* = 24.5 Hz, 6H), 7.07 – 7.02 (m, 3H), 4.54 – 4.11 (m, 4H), 3.74 – 3.58 (m, 1H), 3.23 – 2.90 (m, 3H), 2.74 – 2.60 (m, 1H), 2.44 – 2.27 (m, 2H). ^{13}C NMR (100 MHz, Chloroform-*d*) δ 198.8, 173.2, 152.9, 142.2, 136.6, 135.3, 134.6, 133.2, 128.5, 128.5, 128.0, 127.8, 127.3, 126.2, 126.0 (t, *J* = 6.4 Hz), 125.2, 122.8, 121.7, 50.6, 38.5, 35.5, 30.8. ^{19}F NMR (376 MHz, Chloroform-*d*) 92.53, 93.19, 96.09, 96.75. FT-IR: *ν* (cm^{-1}) 3079, 2364, 2323, 2053, 1996, 1936, 1767, 1245, 1055, 760. HRMS [ESI] calcd for $C_{39}H_{34}F_2N_2O_3S_2Na$ [M+Na]$^+$692.2053, found 692.2011.

4-(benzo[*d*]thiazol-2-yl)-6,6-difluoro-6-(4-(morpholinosulfonyl)phenyl)-1-phenylhexan-1-one (**4k**)

88%, colorless oil.^{1}H NMR (400 MHz, Chloroform-*d*) δ 7.96 (d, *J* = 8.1 Hz, 1H), 7.92 – 7.81 (m, 3H), 7.75 – 7.35 (m, 9H), 3.99 – 3.52 (m, 5H), 3.34 – 2.82 (m, 7H), 2.79 – 2.63 (m, 1H), 2.50 – 2.21 (m, 2H). ^{13}C NMR (100 MHz, Chloroform-*d*) δ 198.8, 173.2, 152.8, 141.4 (t, *J* = 26.8Hz), 136.9, 136.6, 133.2, 128.6, 128.0, 127.9, 126.3, 126.2, 126.1 (t, *J* = 6.3 Hz), 125.3, 122.8, 121.7, 66.1, 45.9, 44.5 (t, *J* = 26.8 Hz), 38.4 (t, *J* = 3.3 Hz), 35.4, 30.9. ^{19}F NMR (376 MHz, Chloroform-*d*) 91.46, 92.12, 96.74, 97.41. FT-IR: *v* (cm^{-1}) 3079, 2321, 2206, 2137, 2056, 1013, 1766, 1061, 771. HRMS [ESI] calcd for $C_{29}H_{28}F_2N_2O_4S_2Na$ [M+Na]$^+$593.1351, found 593.1332.

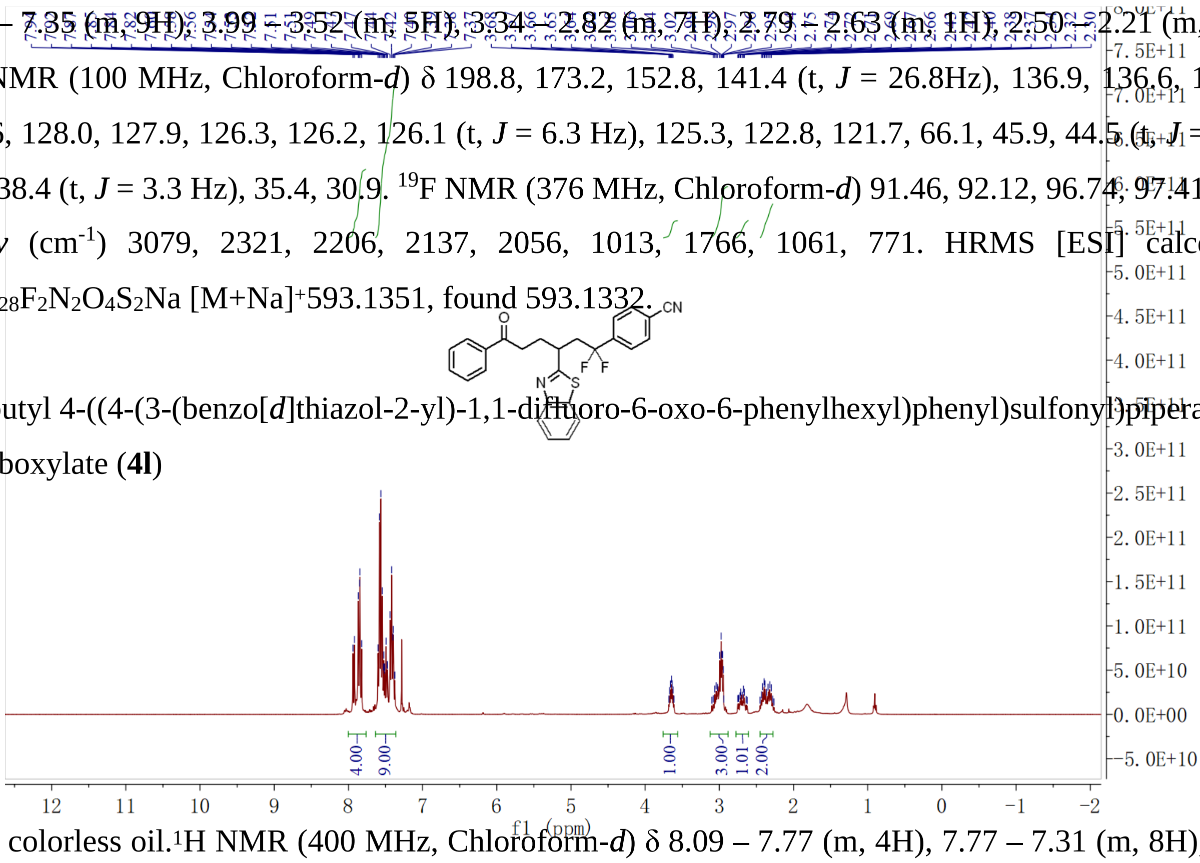

tert-butyl 4-((4-(3-(benzo[*d*]thiazol-2-yl)-1,1-difluoro-6-oxo-6-phenylhexyl)phenyl)sulfonyl)piperazine-1-carboxylate (**4l**)

82%, colorless oil.^{1}H NMR (400 MHz, Chloroform-*d*) δ 8.09 – 7.77 (m, 4H), 7.77 – 7.31 (m, 8H), 7.00 – 6.79 (m, 1H), 3.73 – 3.48 (m, 4H), 3.33 – 2.57 (m, 7H), 2.56 – 2.22 (m, 2H), 1.87 – 1.24 (m, 11H). ^{13}C NMR (100 MHz, Chloroform-*d*) δ 198.8, 173.2, 154.2, 152.7, 137.3, 134.5, 133.2, 128.6, 128.0, 127.8, 126.3, 125.3, 122.8, 121.7, 119.8, 118.8, 45.7, 44.4 (t, *J* = 27.7 Hz), 38.4, 35.4, 30.9, 28.3. ^{19}F NMR (376 MHz, Chloroform-*d*) 91.03, 91.70, 97.04, 97.70. FT-IR: *v* (cm^{-1}) 3089, 2507, 2319, 2139, 1933, 1701, 1251, 1167, 1055, 767.   HRMS [ESI] calcd for $C_{34}H_{37}F_2N_3O_5S_2Na$ [M+Na]$^+$692.2053, found 692.2011.

(S)-4-(1,1-difluoro-3-formyl-6-oxo-6-phenylhexyl)benzonitrile (**9**)

32%, coloress oil. ^{1}H NMR (400 MHz, Chloroform-*d*) δ 9.68 (s, 1H), 7.92 (d, *J* = 8.1 Hz, 2H), 7.74 (d, *J* = 8.1 Hz, 2H), 7.64– 7.55 (m, 3H), 7.48 (d, *J* = 7.7 Hz, 2H), 3.12– 2.95 (m, 2H), 2.80– 2.74 (m, 1H),

2.23– 2.15 (m, 2H), 2.05 – 1.98 (m, 2H). ^{13}C NMR (100 MHz, Chloroform-*d*) δ 201.5, 133.4, 132.6, 128.7, 128.0, 35.0, 29.3, 27.2, 23.4. ^{19}F NMR (376 MHz, Chloroform-*d*) 94.6, 95.2, 95.7, 96.4. HRMS [ESI] calcd for $C_{20}H_{17}F_2NO_2$ $[M+Cl]^-$ 376.0921, found 376.0947.

9. The Sprctra of products

Supplementary Figure 17. 3a ^1H NMR (400 MHz, $CDCl_3$)

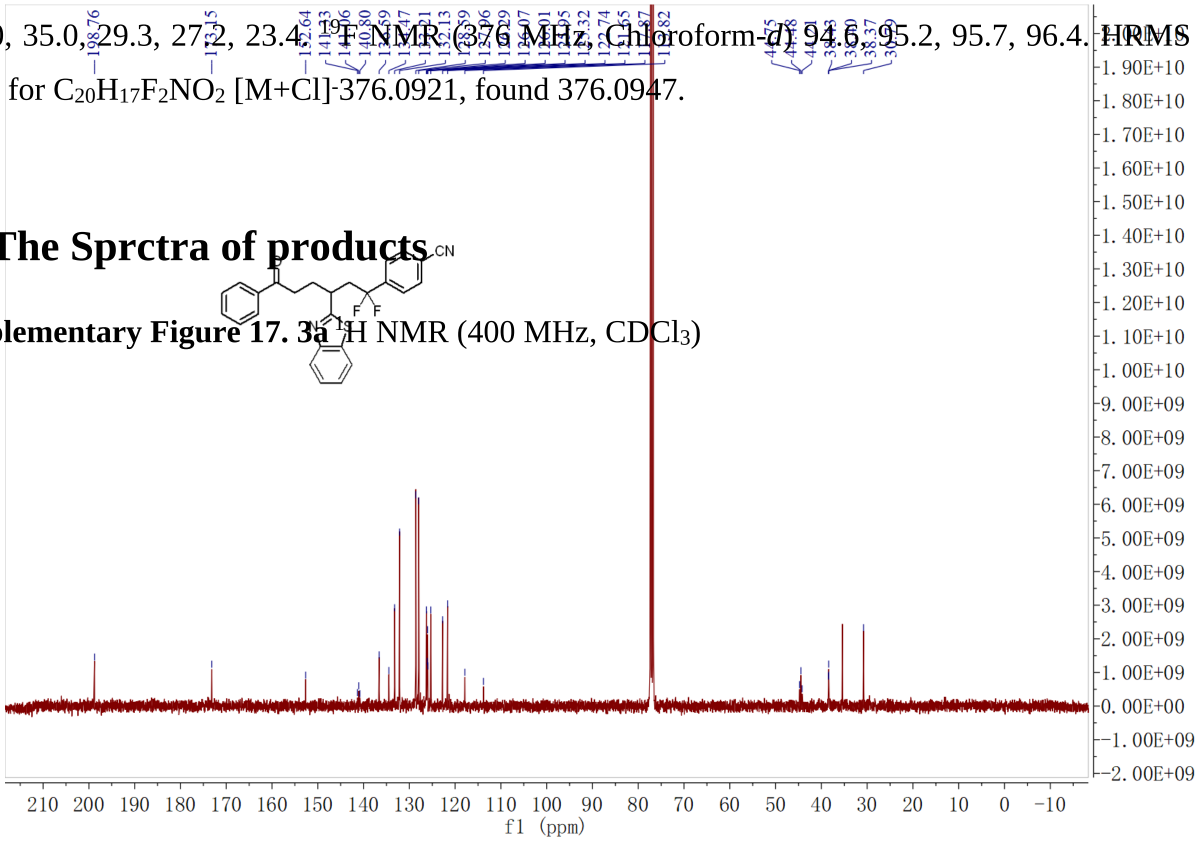

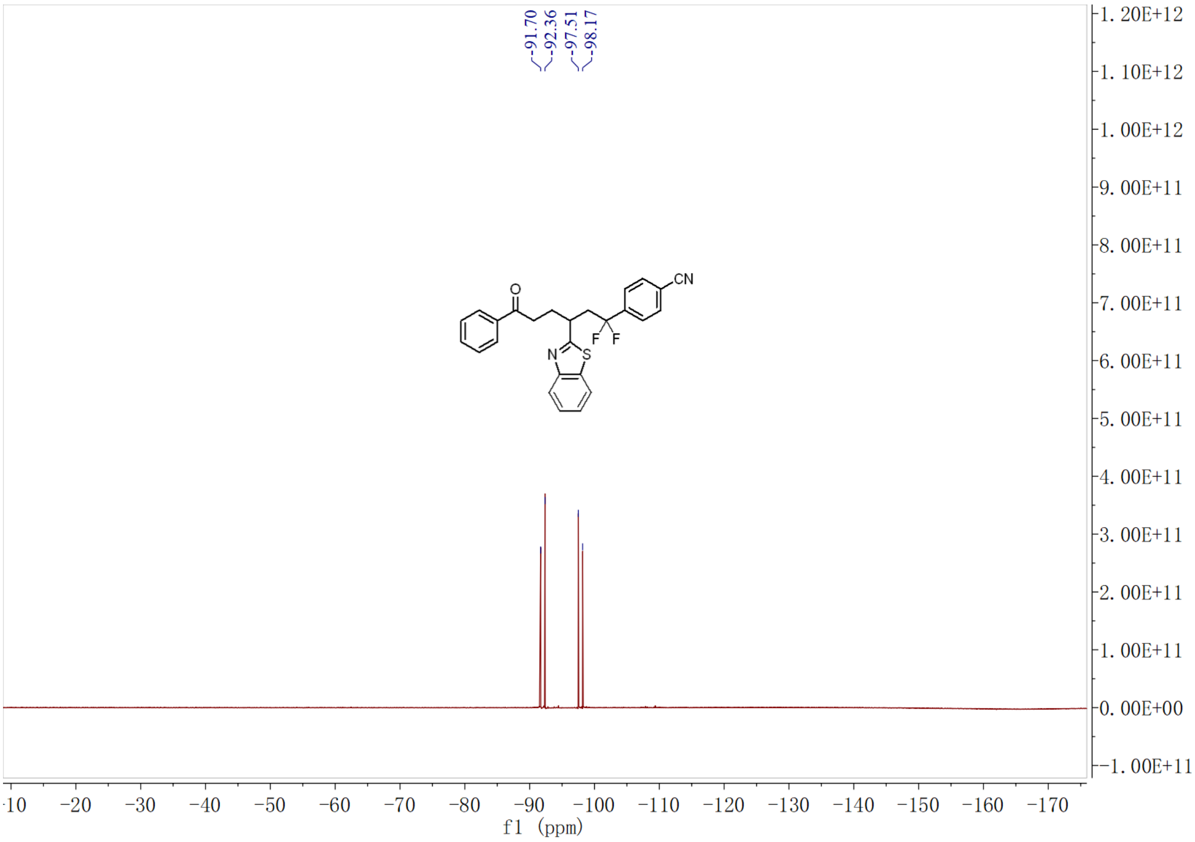

Supplementary Figure 18. 3a ^{13}C NMR (100 MHz, $CDCl_3$)

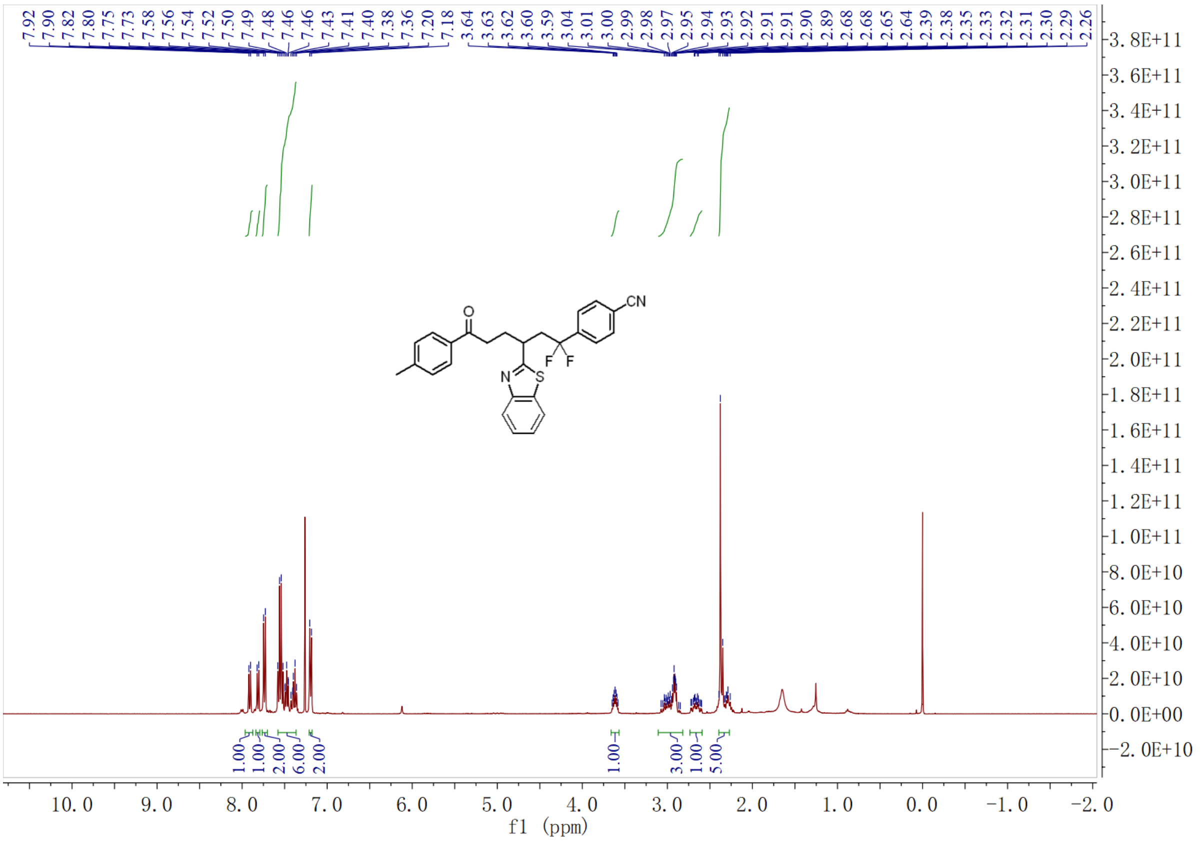

Supplementary Figure 19. 3a ^{19}F NMR (376 MHz, $CDCl_3$)

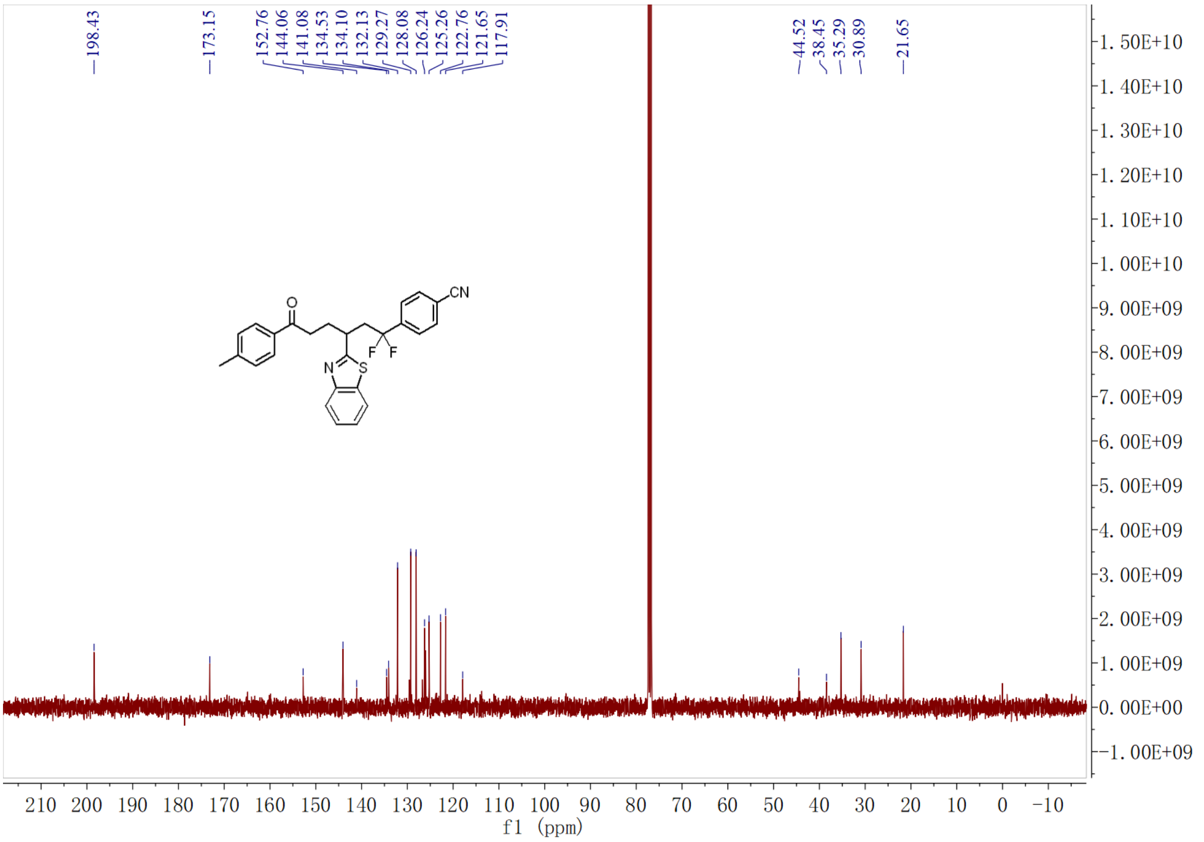

Supplementary Figure 20. 3b ^{1}H NMR (400 MHz, CDCl3)

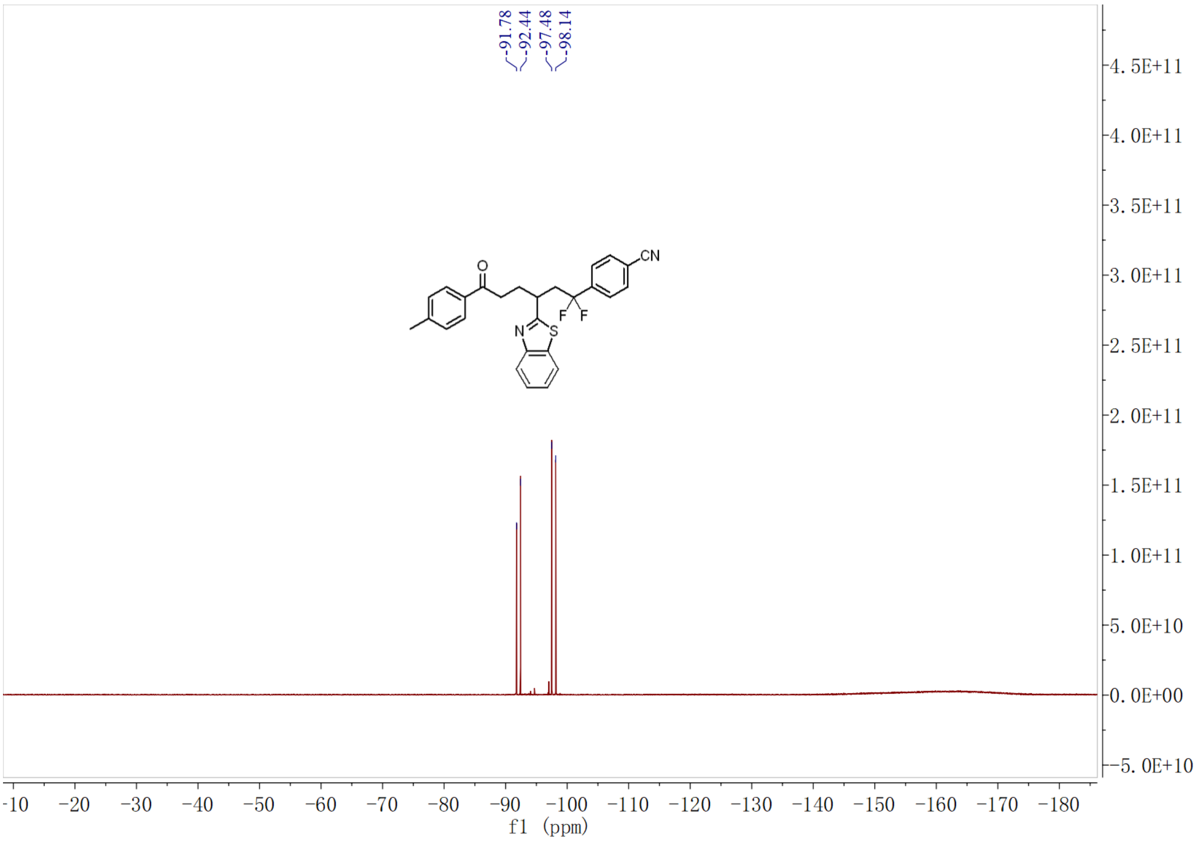

Supplementary Figure 21. 3b ^{13}C NMR (100 MHz, CDCl3)

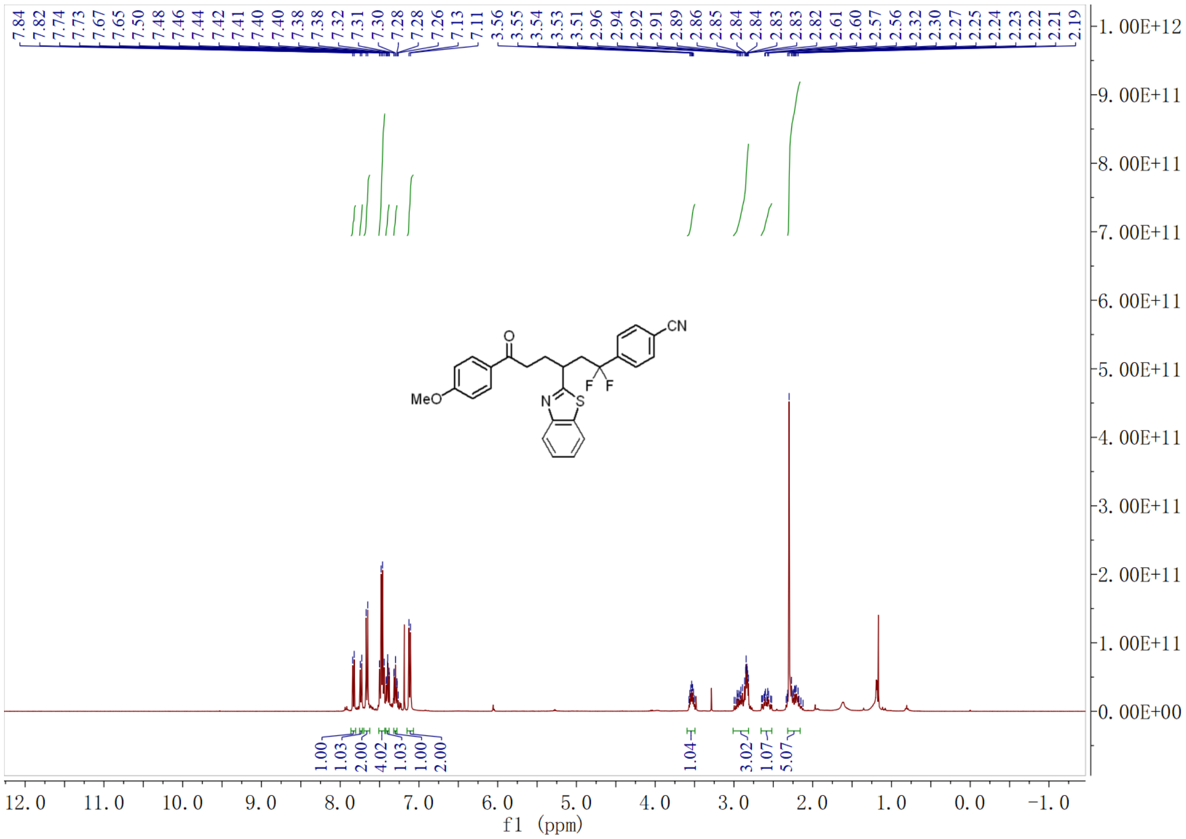

Supplementary Figure 22. 3b ^{19}F NMR (376 MHz, $CDCl_3$)

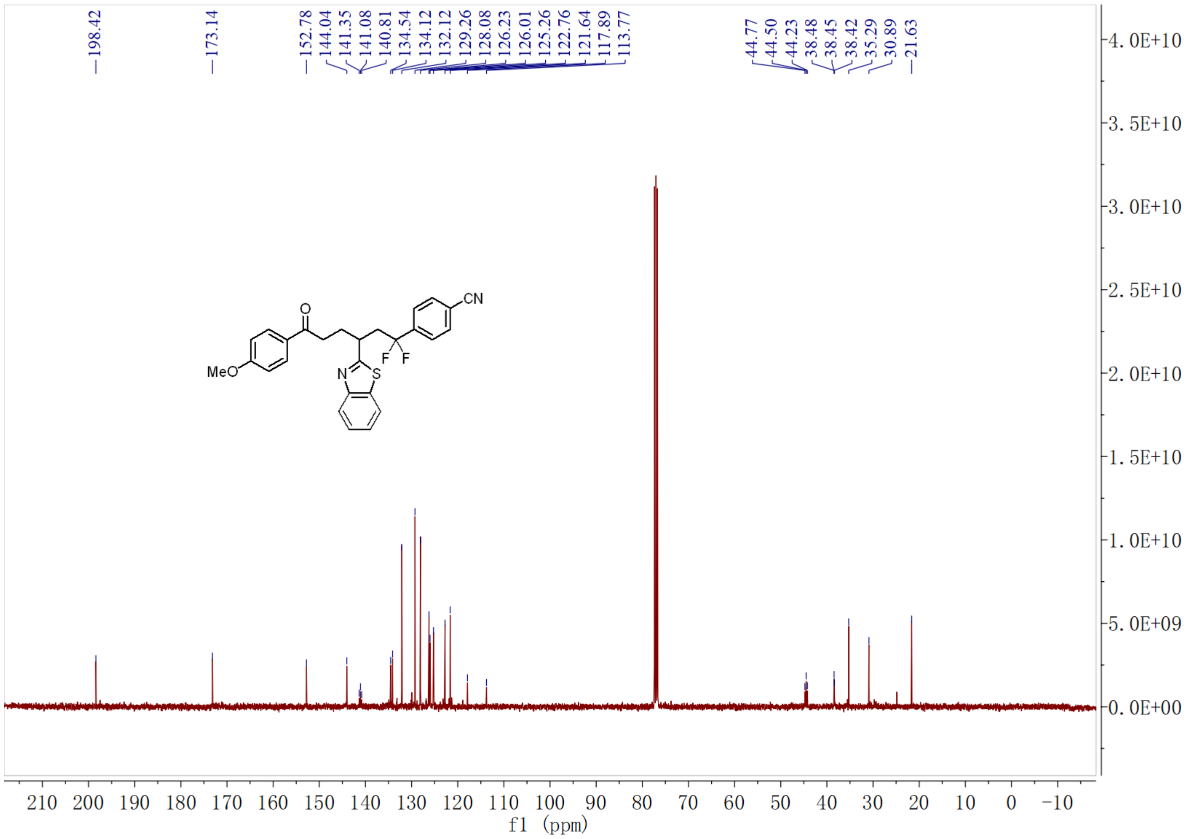

Supplementary Figure 23. 3c ^{1}H NMR (400 MHz, $CDCl_3$)

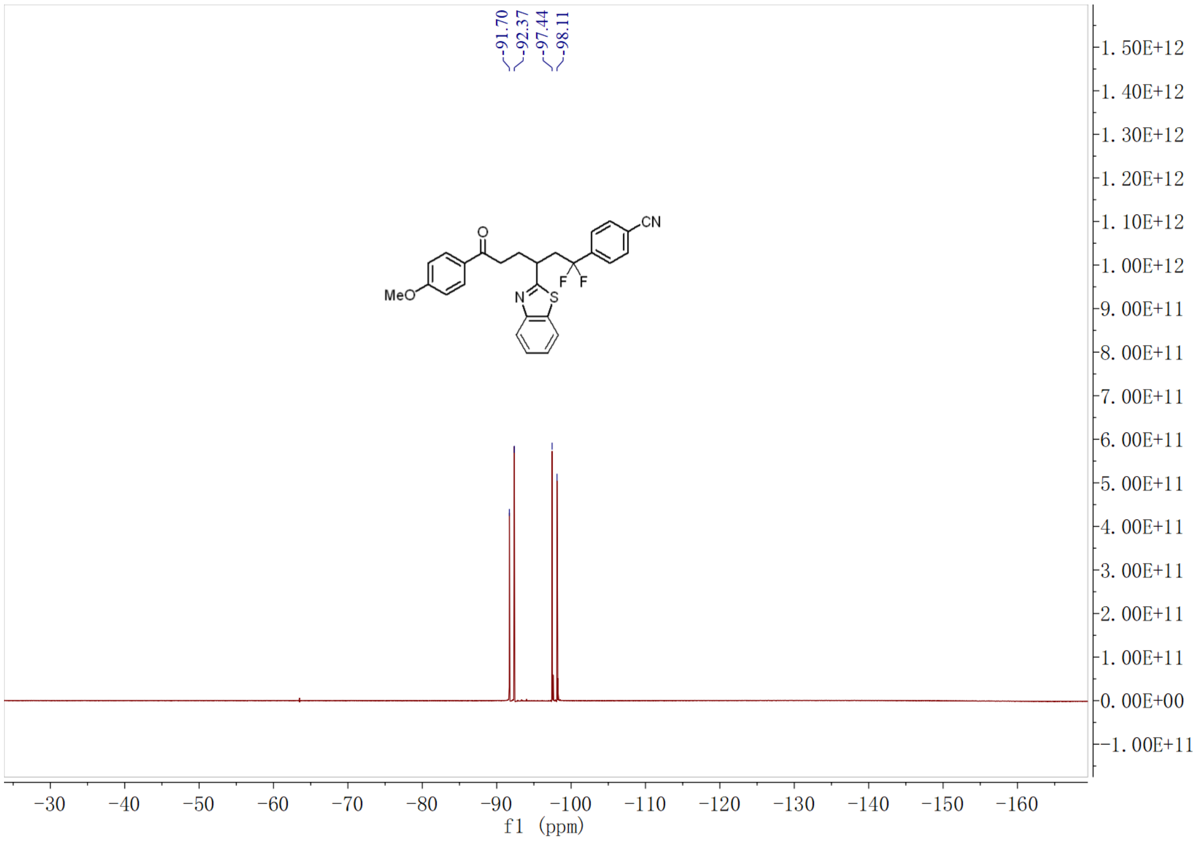

Supplementary Figure 24. 3c ^{13}C NMR (100 MHz, $CDCl_3$)

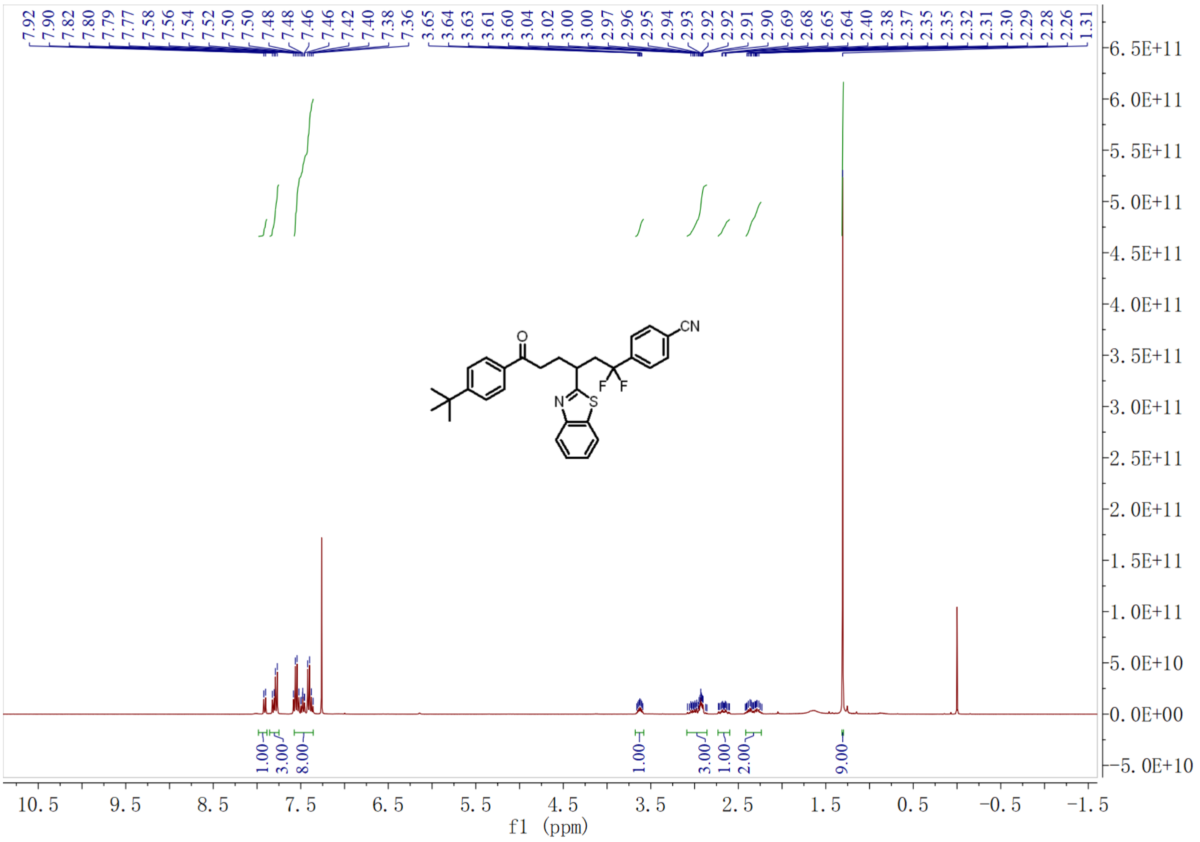

Supplementary Figure 25. 3c ^{19}F NMR (376 MHz, $CDCl_3$)

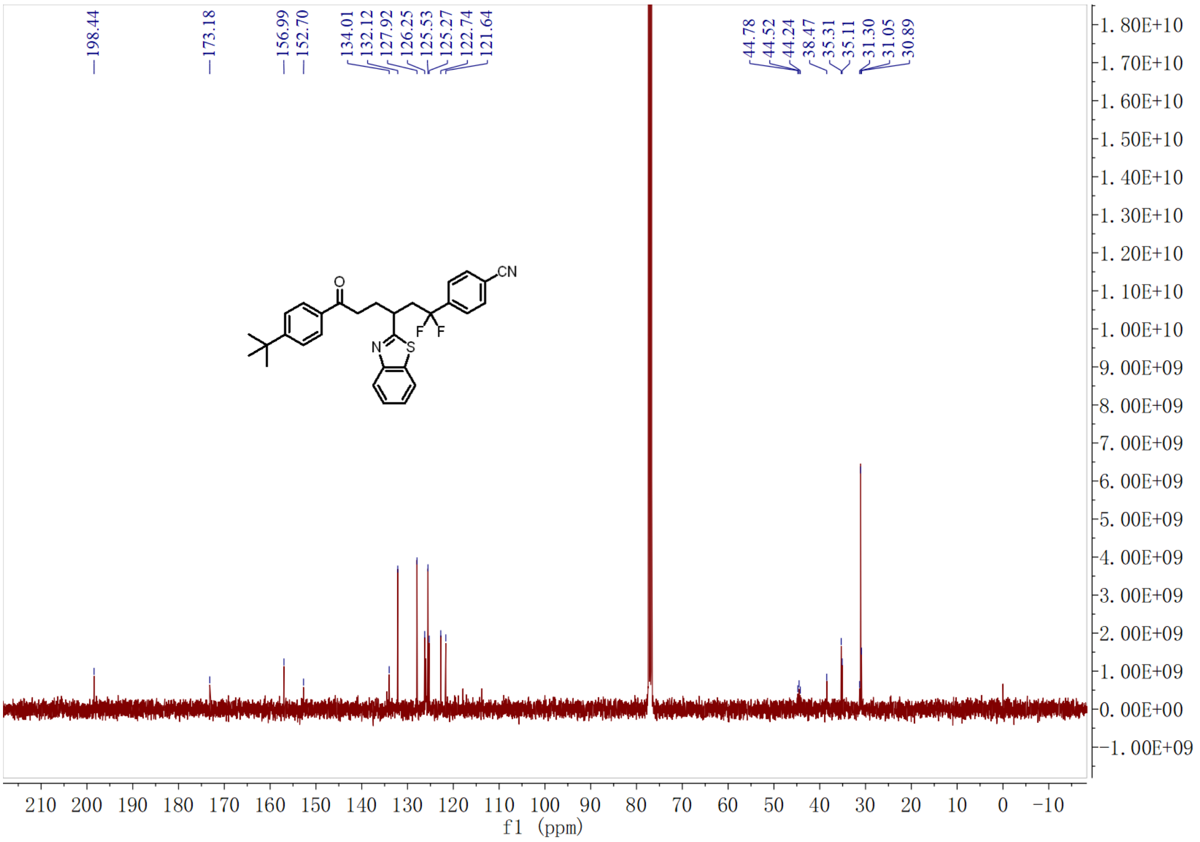

Supplementary Figure 26. 3d ^{1}H NMR (400 MHz, $CDCl_3$)

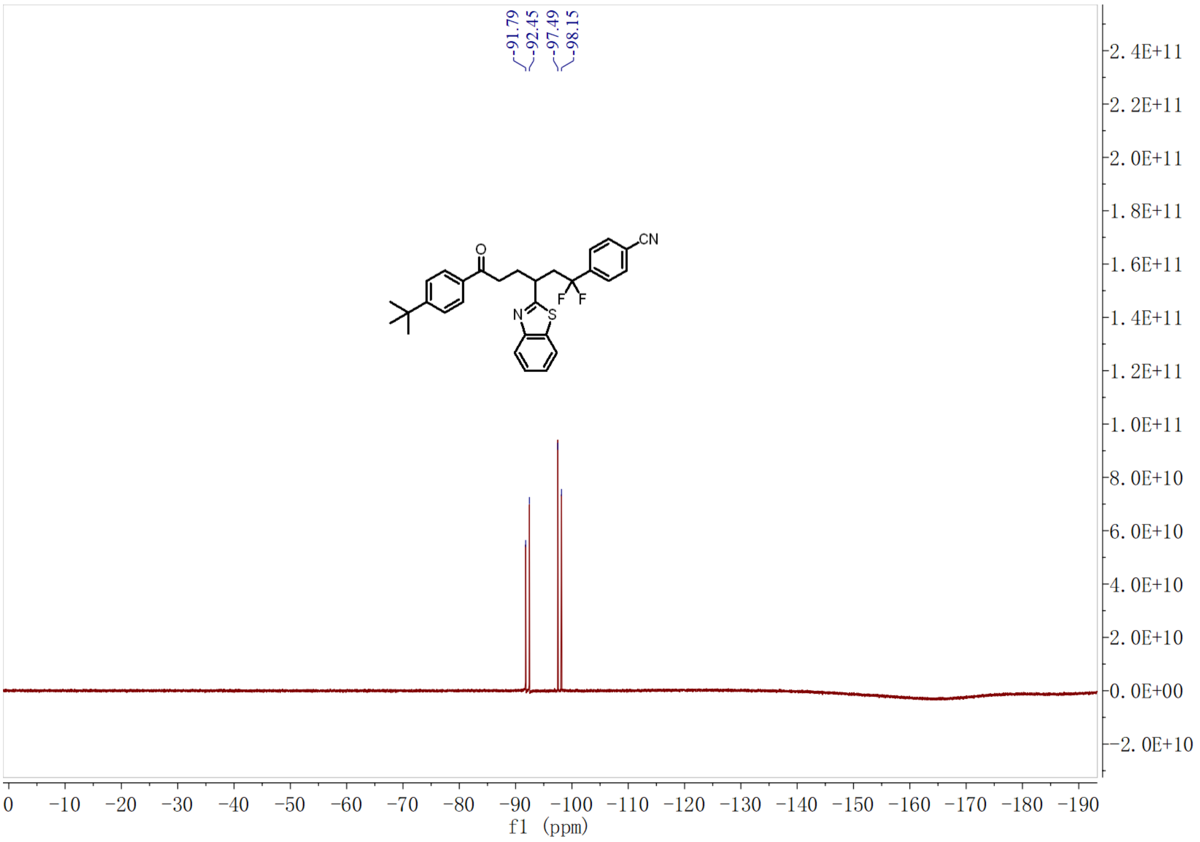

Supplementary Figure 27. 3d ^{13}C NMR (100 MHz, $CDCl_3$)

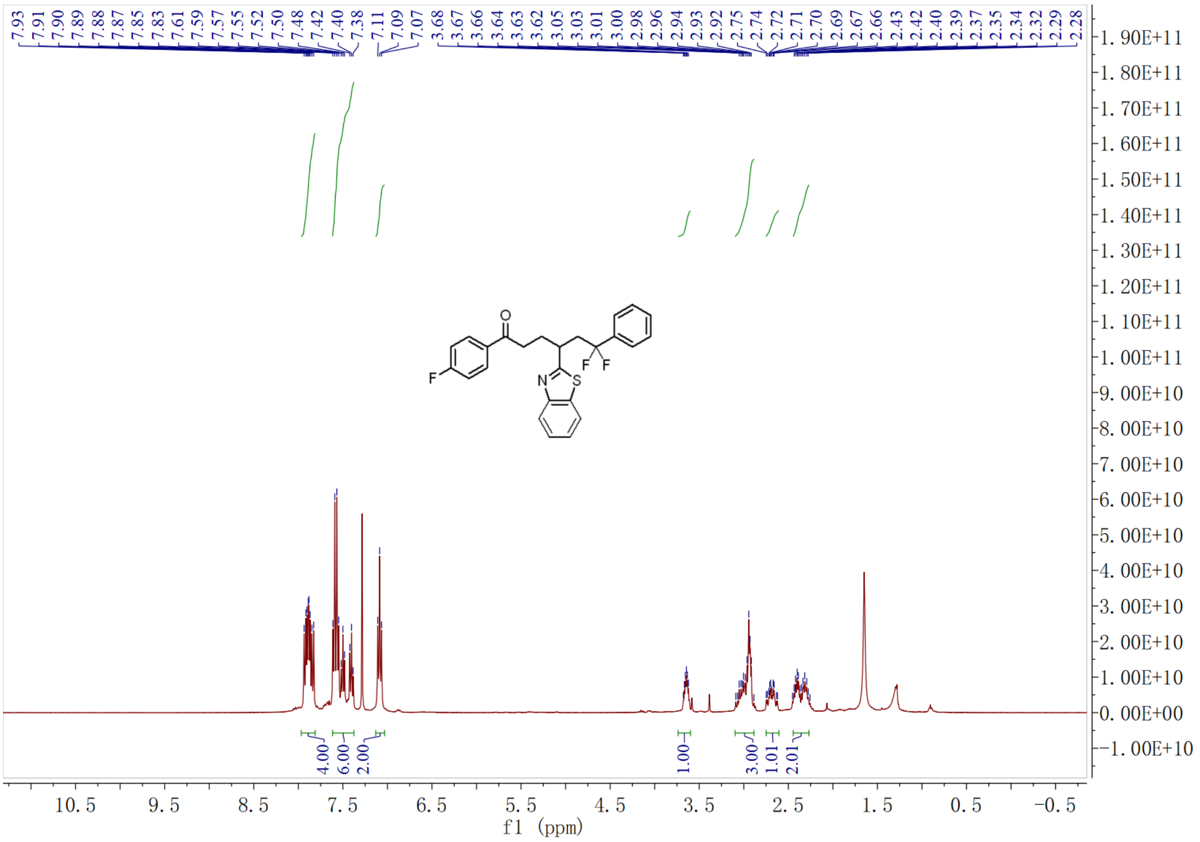

Supplementary Figure 28. 3d ^{19}F NMR (376 MHz, $CDCl_3$)

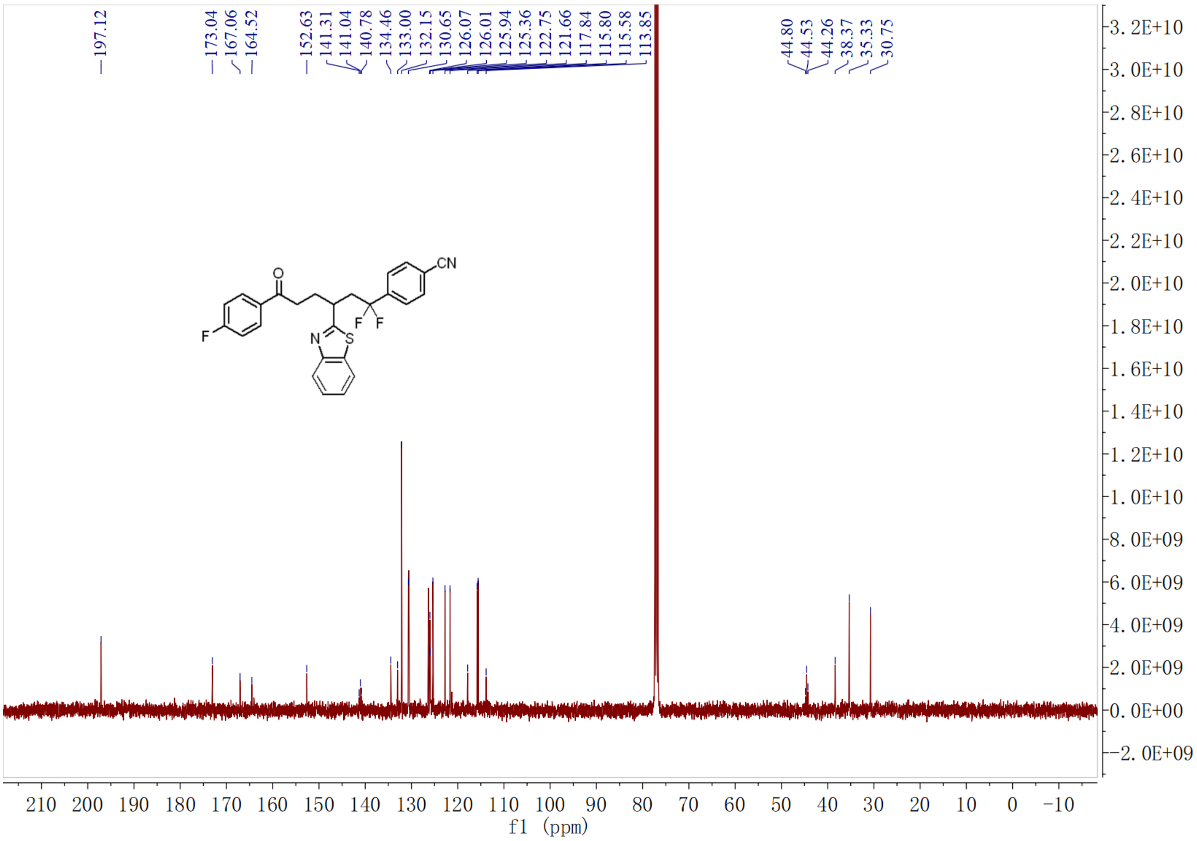

Supplementary Figure 29. 3e ^{1}H NMR (400 MHz, $CDCl_3$)

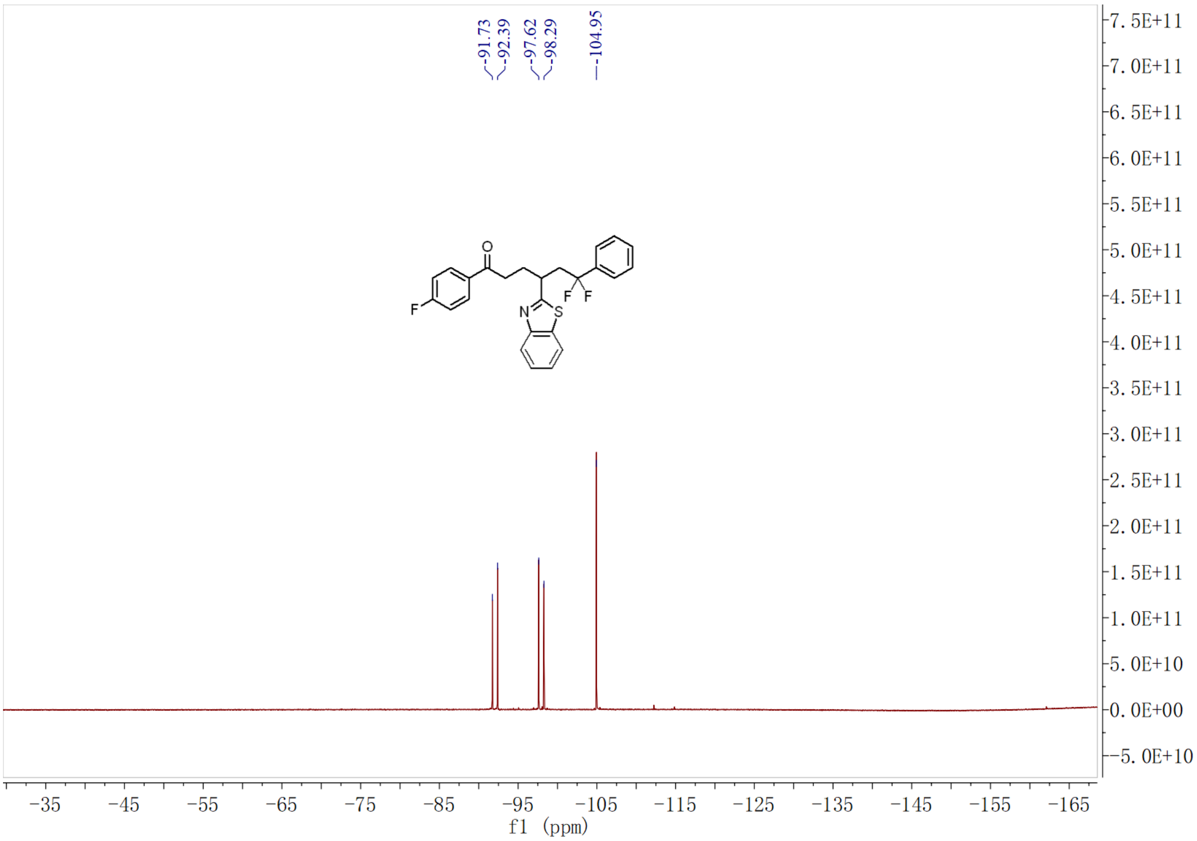

Supplementary Figure 30. 3e ^{13}C NMR (100 MHz, $CDCl_3$)

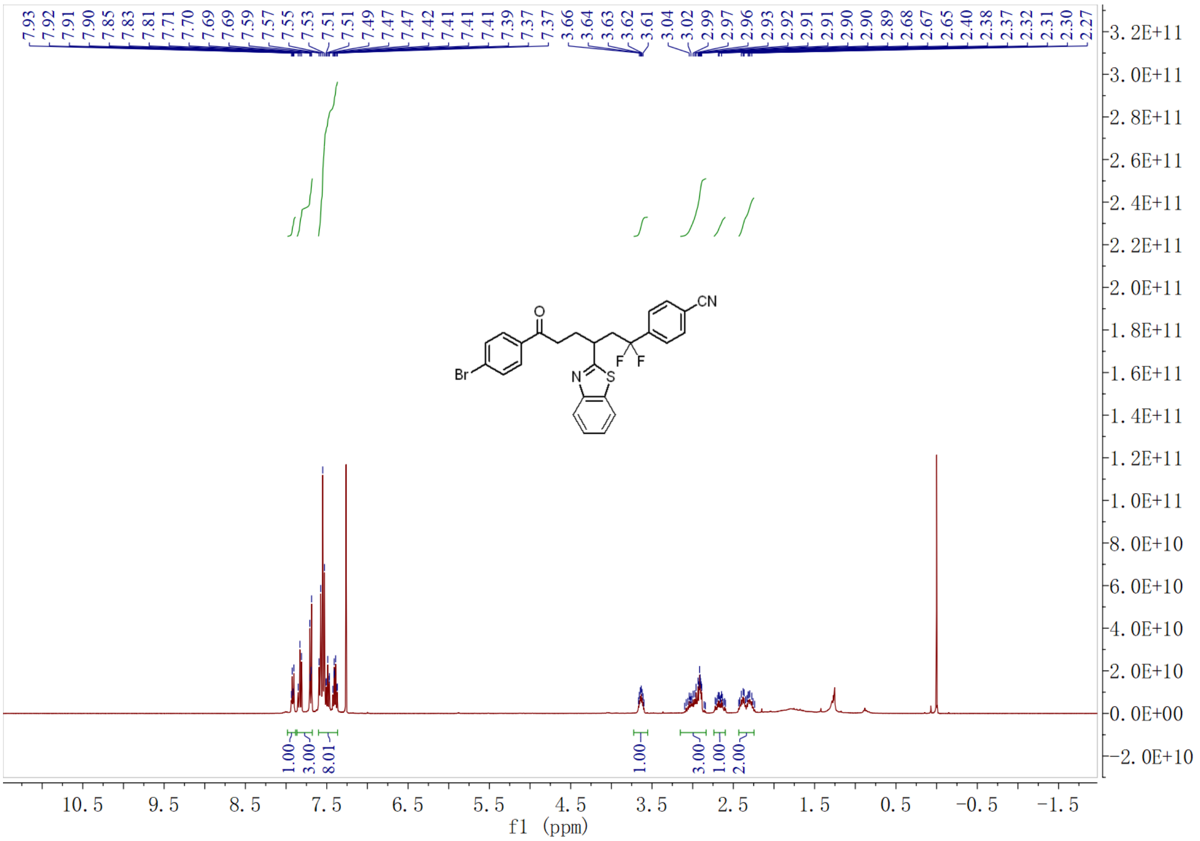

Supplementary Figure 31. 3e ^{19}F NMR (376 MHz, $CDCl_3$)

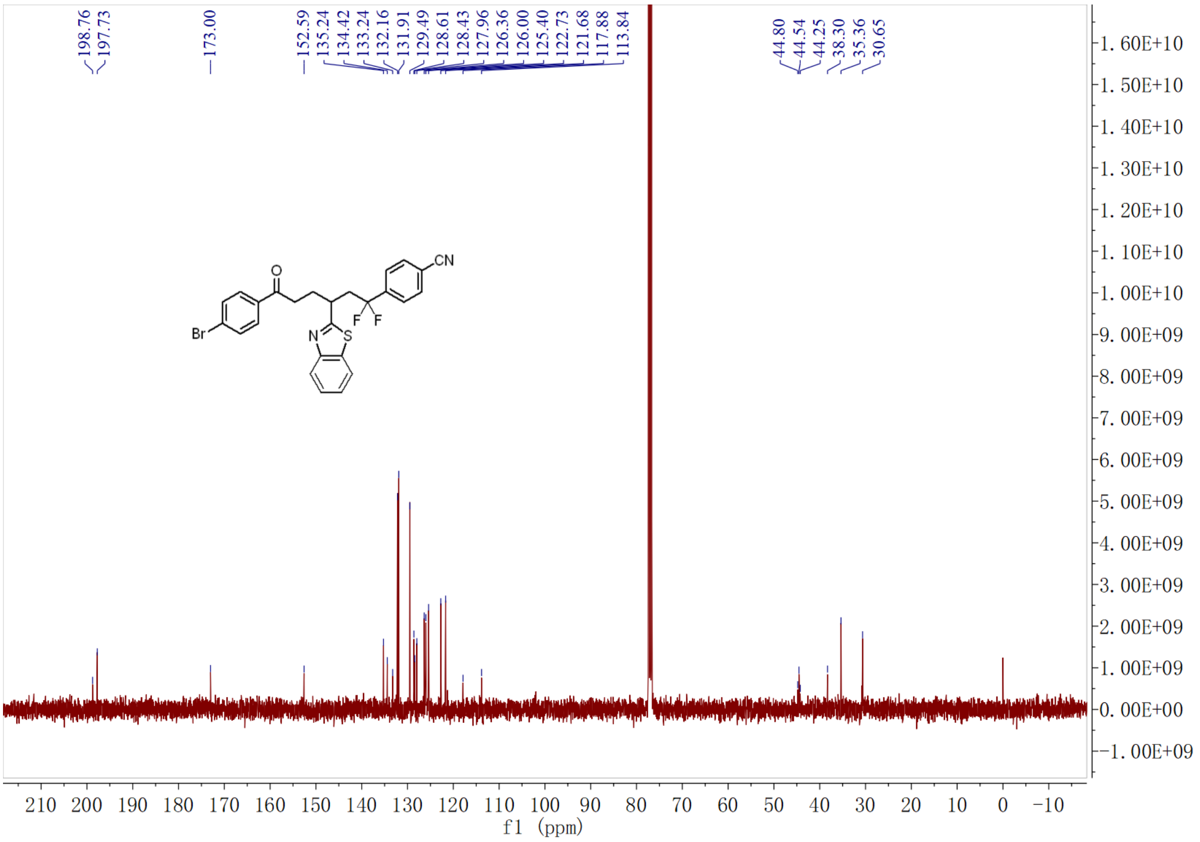

Supplementary Figure 32. 3f ^{1}H NMR (400 MHz, $CDCl_3$)

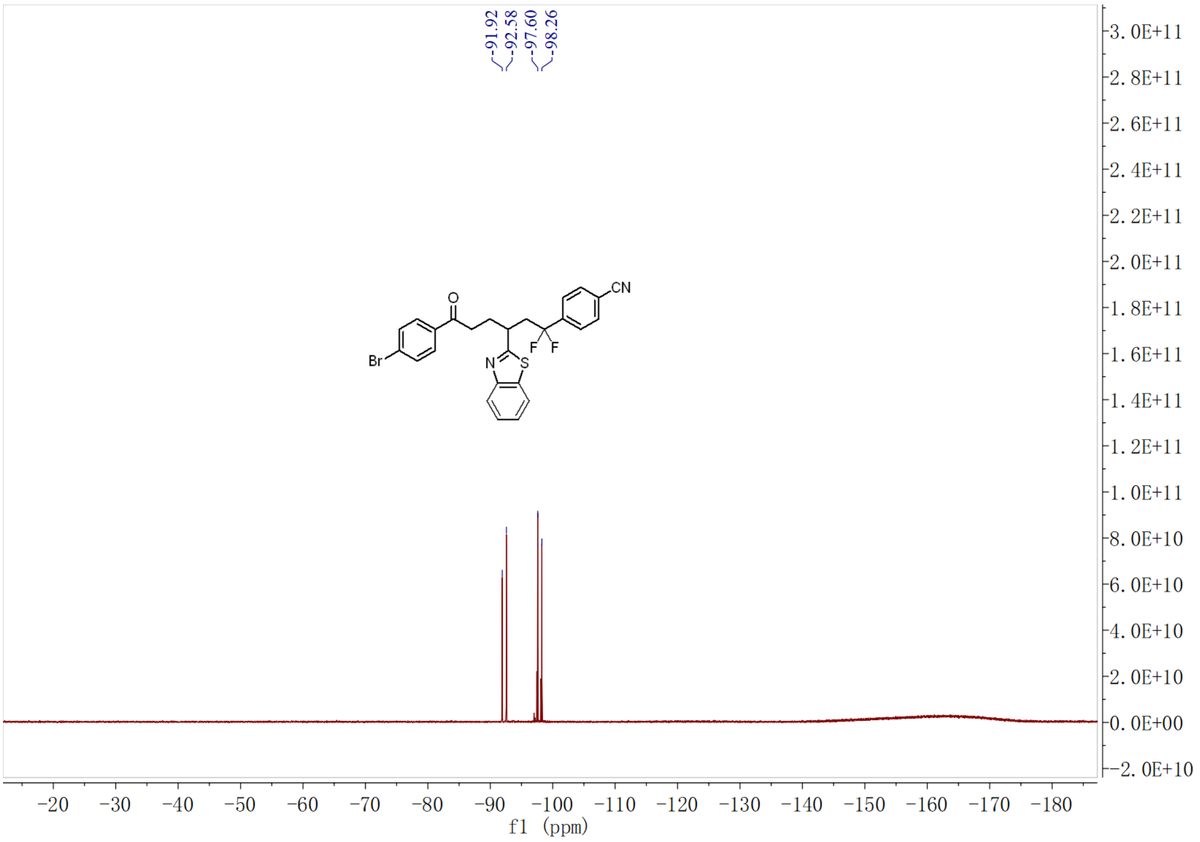

Supplementary Figure 33. 3f ^{13}C NMR (100 MHz, $CDCl_3$)

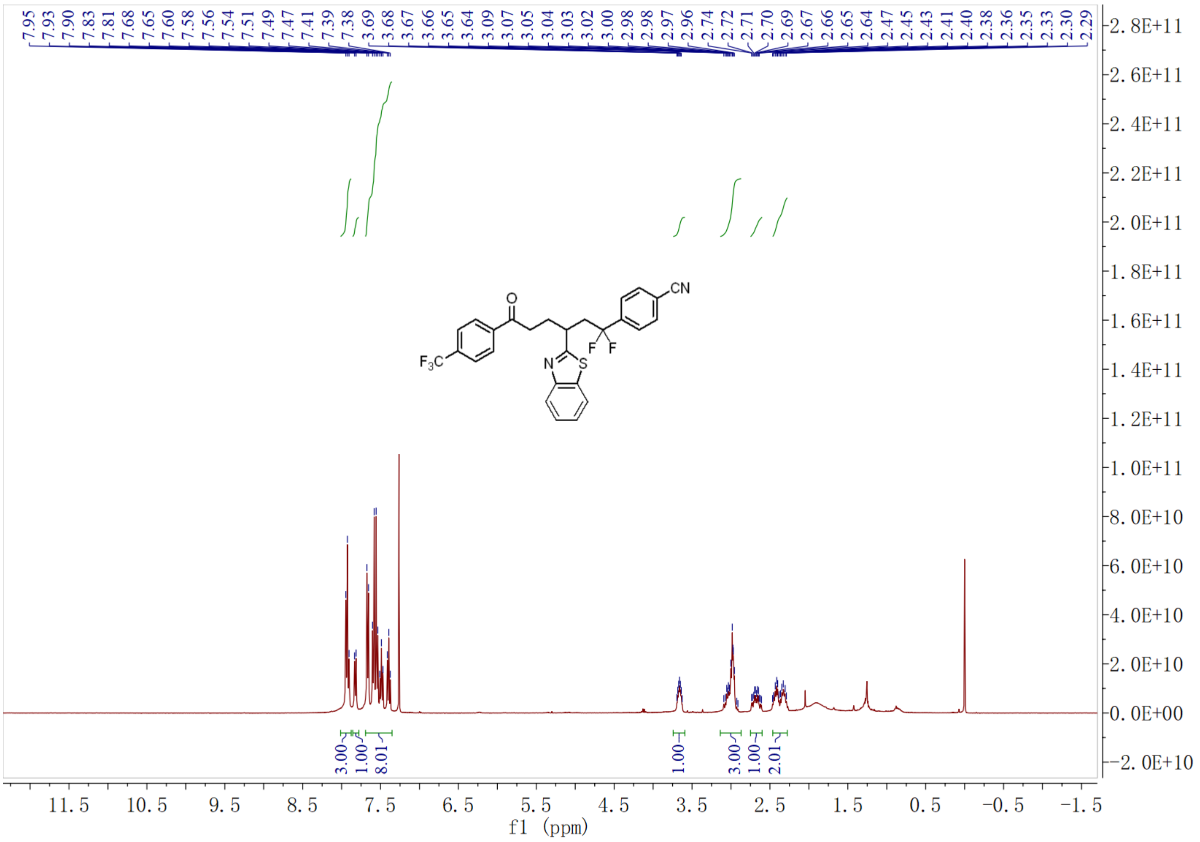

Supplementary Figure 34. 3f ^{19}F NMR (376 MHz, $CDCl_3$)

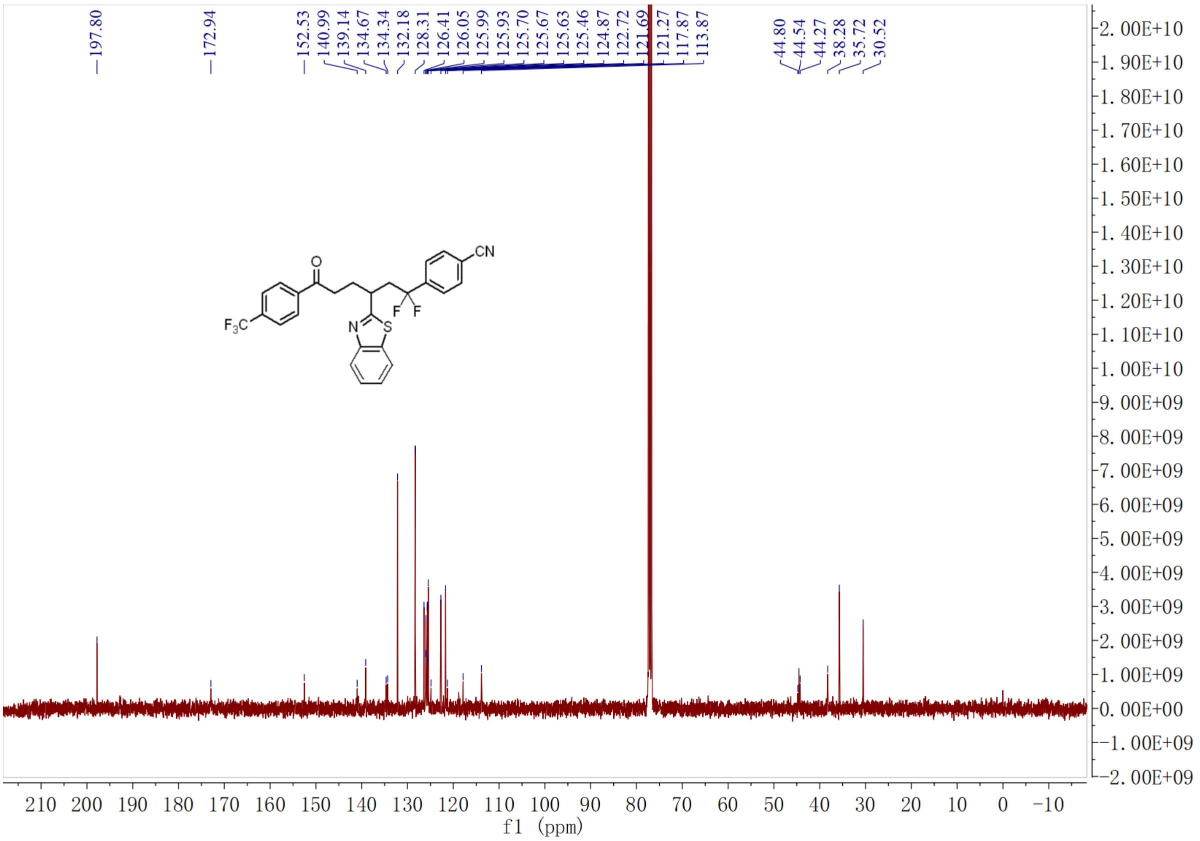

Supplementary Figure 35. 3g ^{1}H NMR (400 MHz, $CDCl_3$)

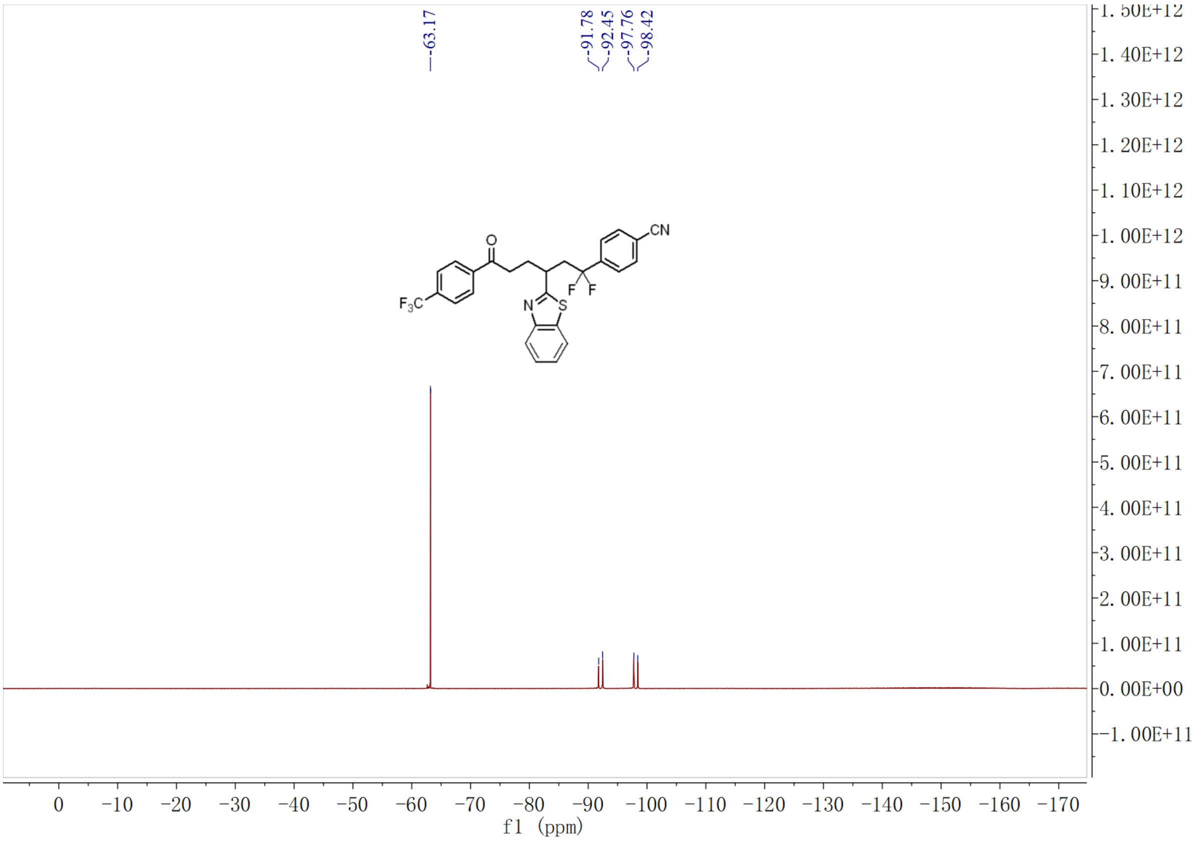

Supplementary Figure 36. 3g ^{13}C NMR (100 MHz, $CDCl_3$)

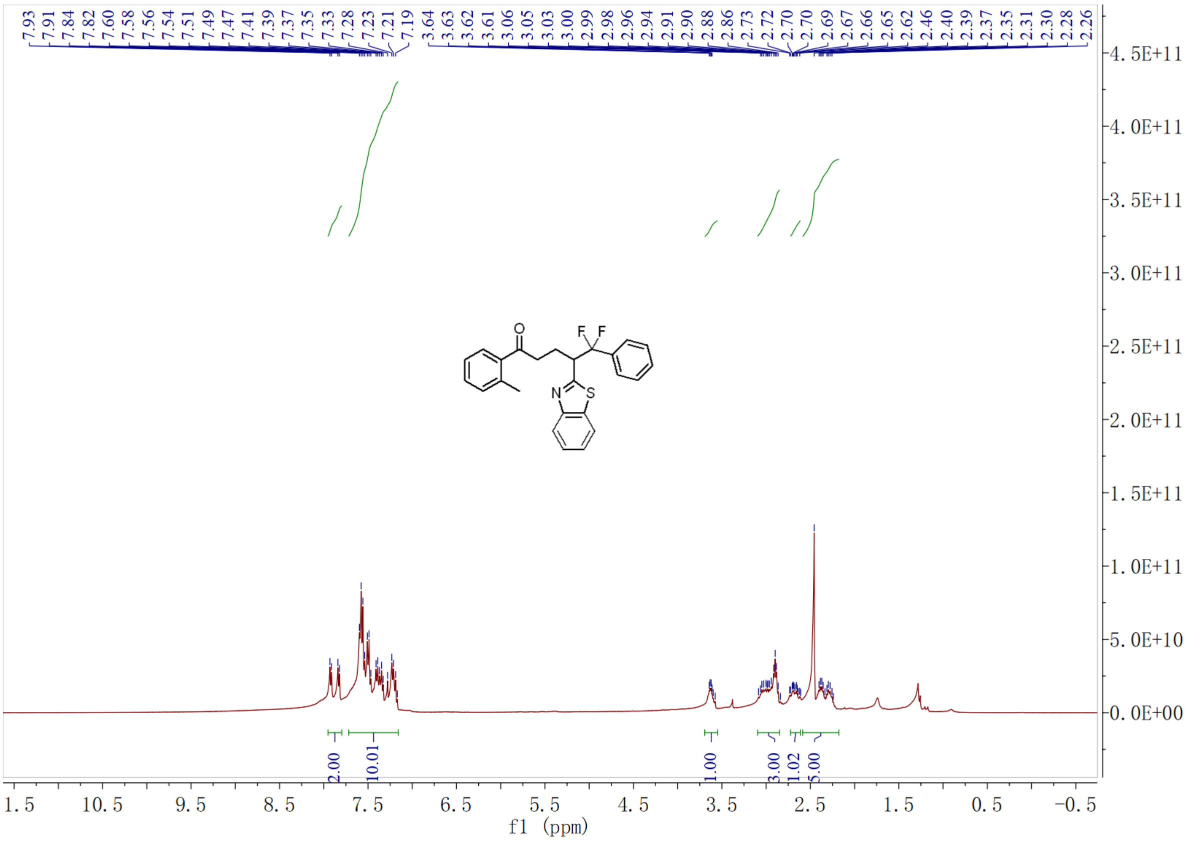

Supplementary Figure 37. 3g ^{19}F NMR (376 MHz, $CDCl_3$)

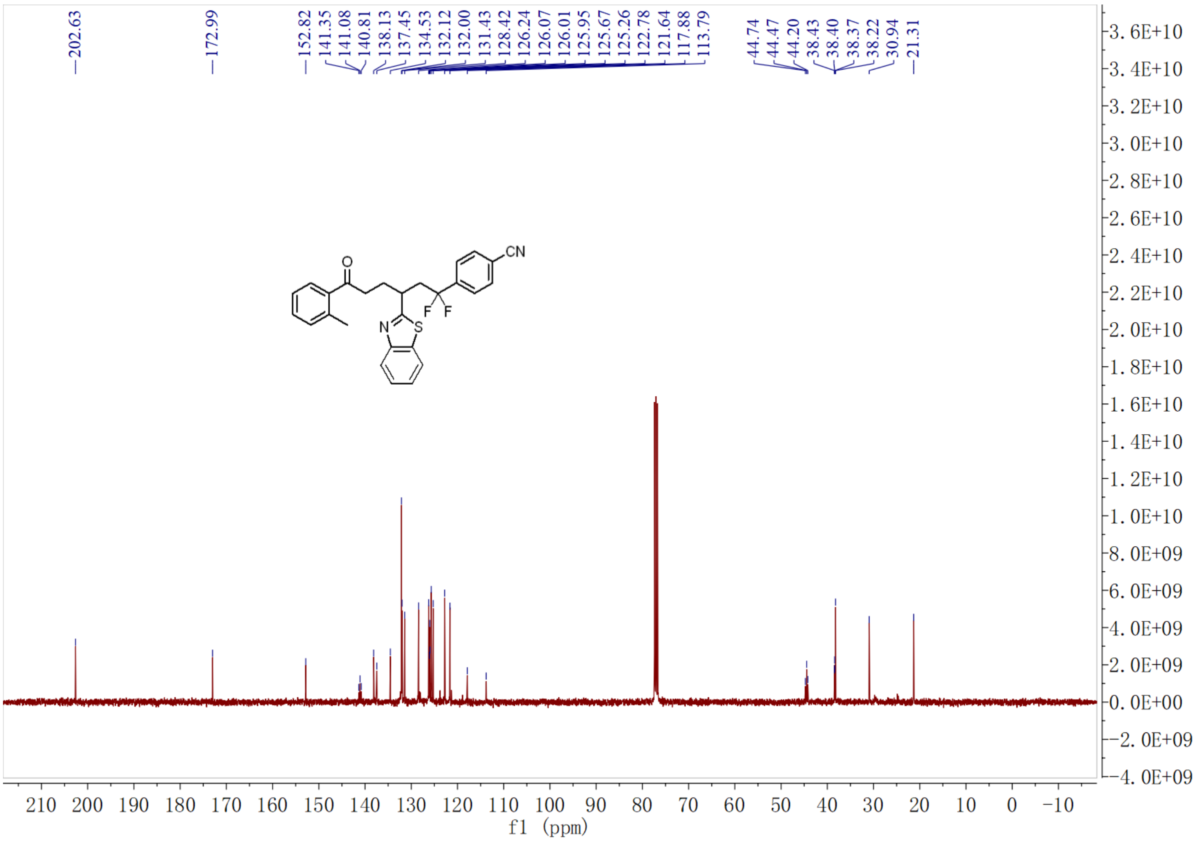

Supplementary Figure 38. 3h ^{1}H NMR (400 MHz, $CDCl_3$)

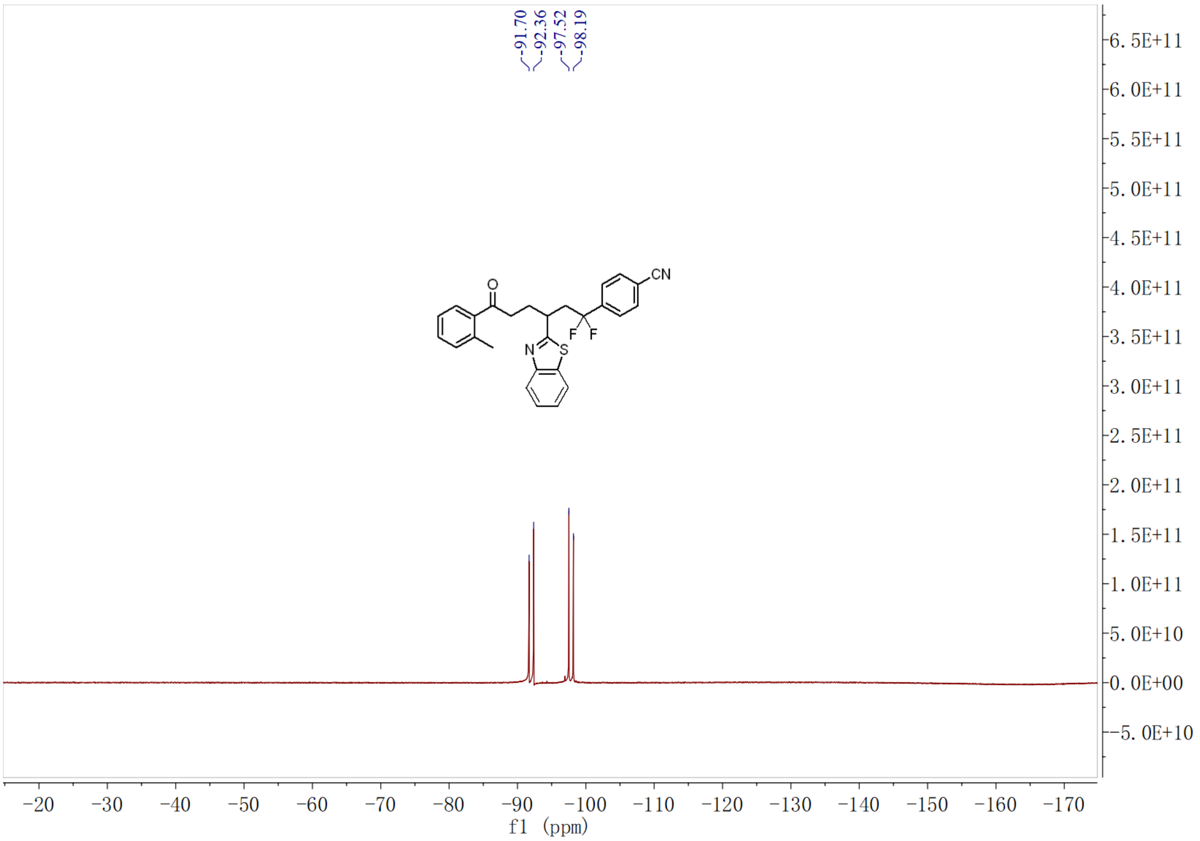

Supplementary Figure 39. 3h ^{13}C NMR (100 MHz, $CDCl_3$)

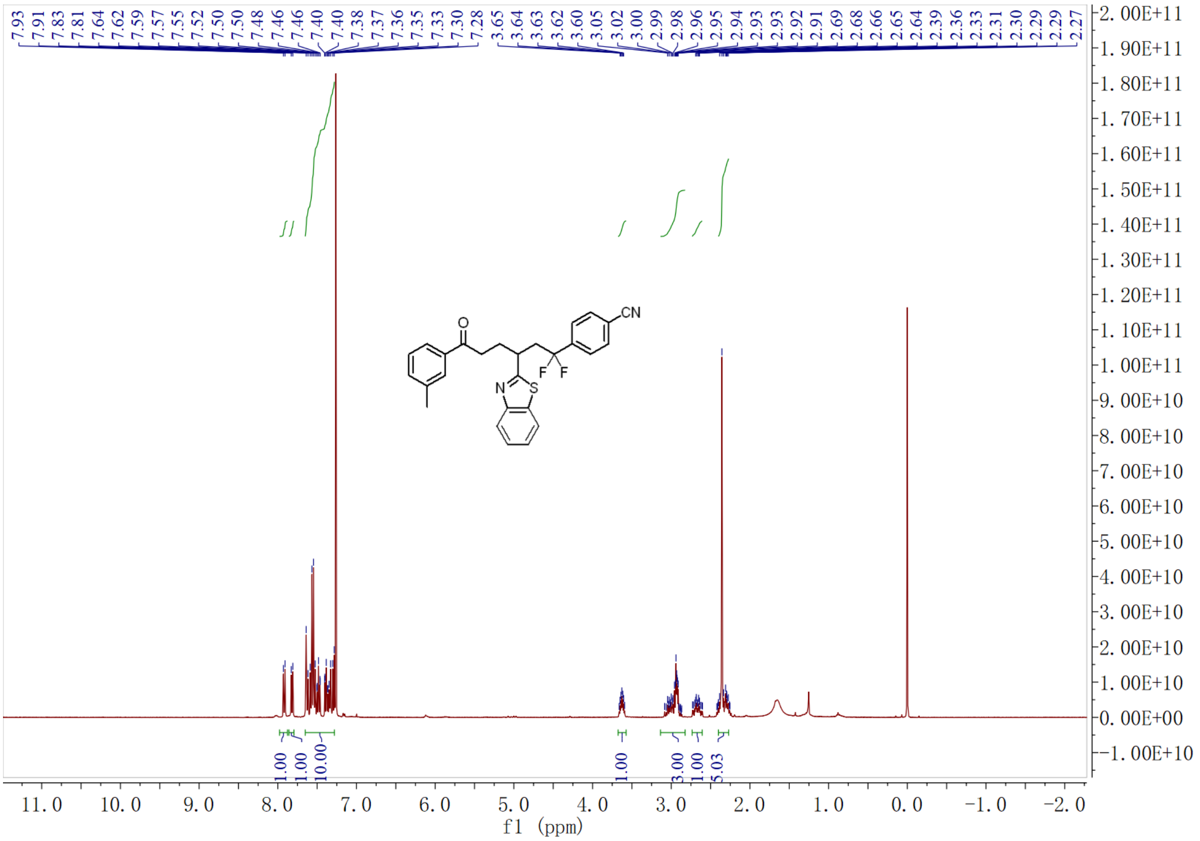

Supplementary Figure 40. 3h ^{19}F NMR (376 MHz, $CDCl_3$)

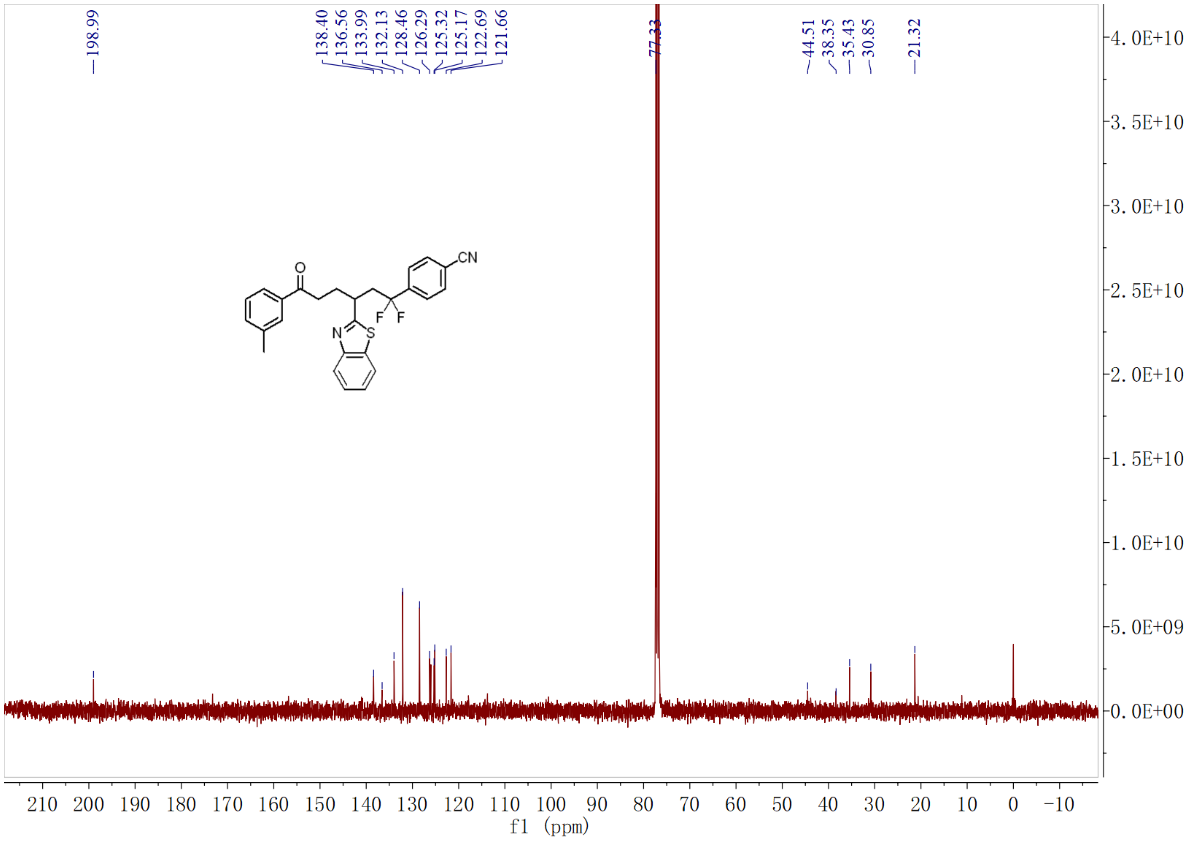

Supplementary Figure 41. 3i ^{1}H NMR (400 MHz, $CDCl_3$)

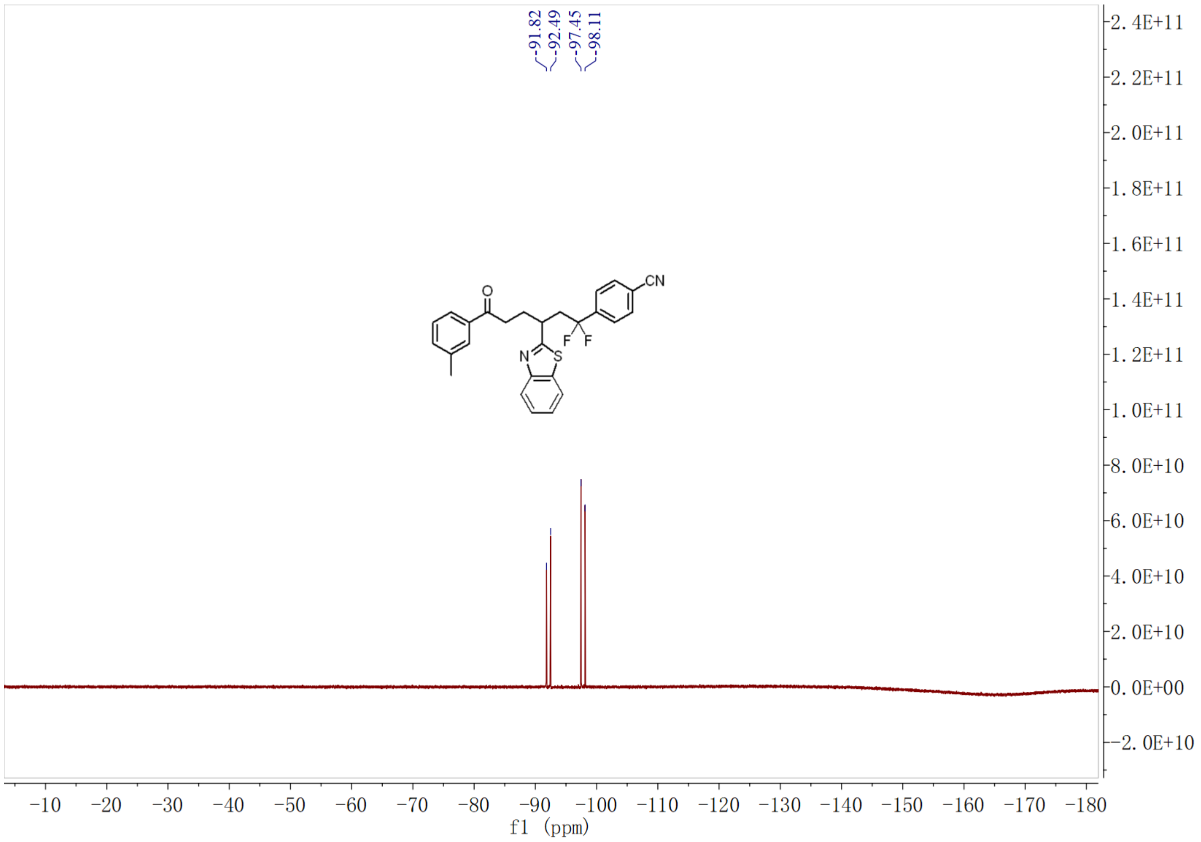

Supplementary Figure 42. 3i ^{13}C NMR (100 MHz, $CDCl_3$)

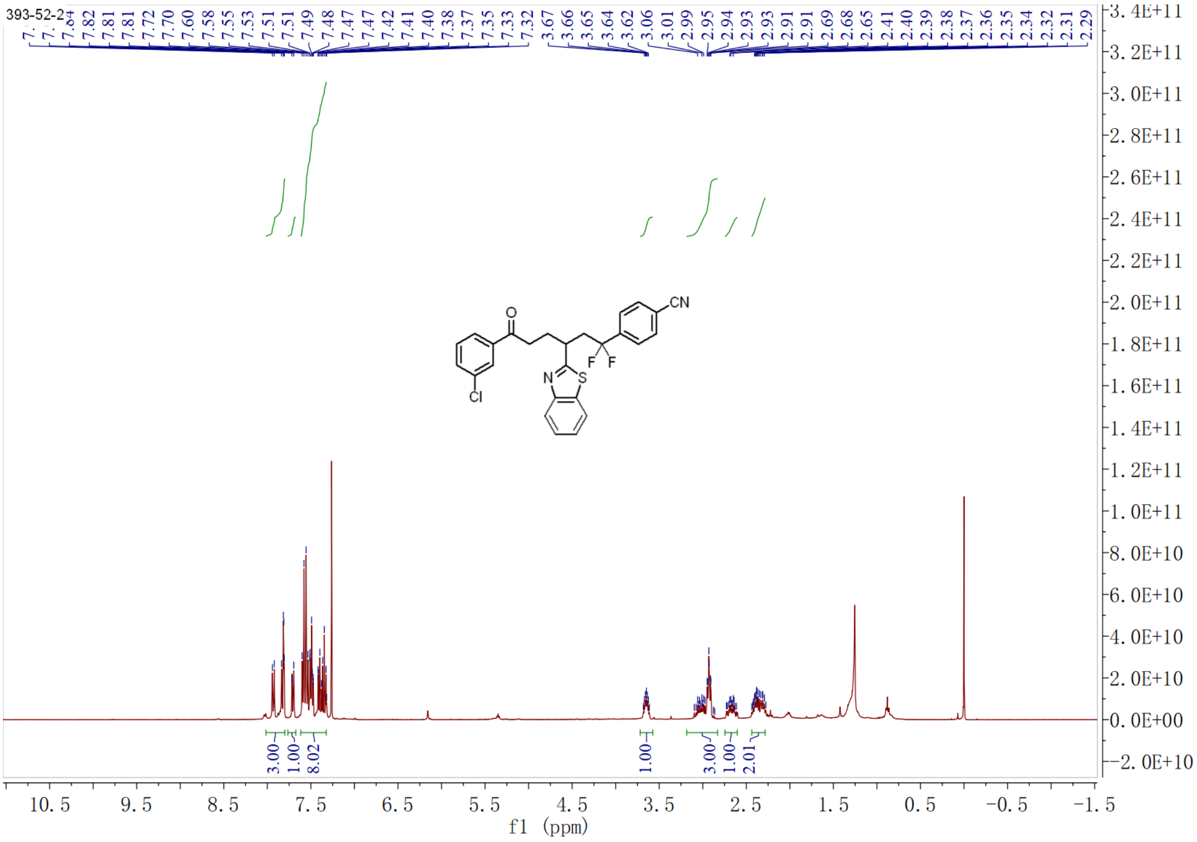

Supplementary Figure 43. 3i ^{19}F NMR (376 MHz, $CDCl_3$)

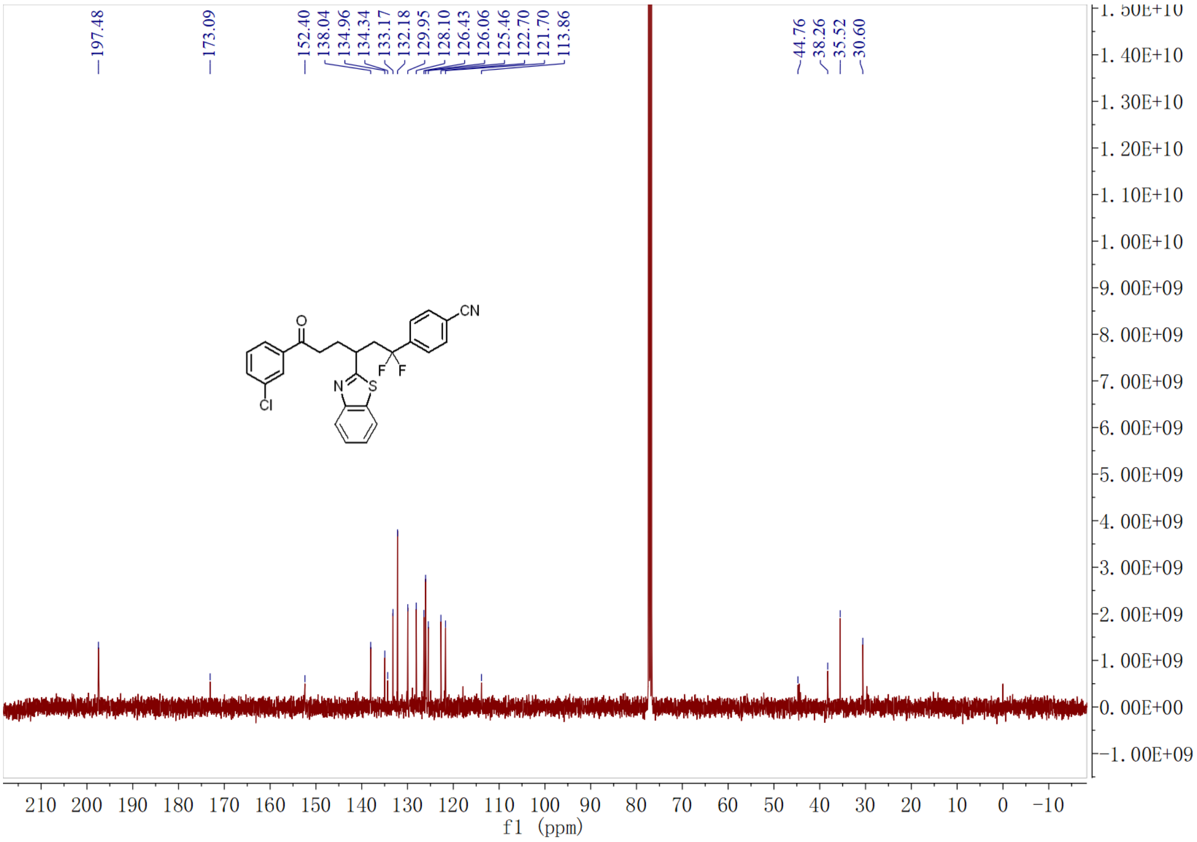

Supplementary Figure 44. 3j ^{1}H NMR (400 MHz, $CDCl_3$)

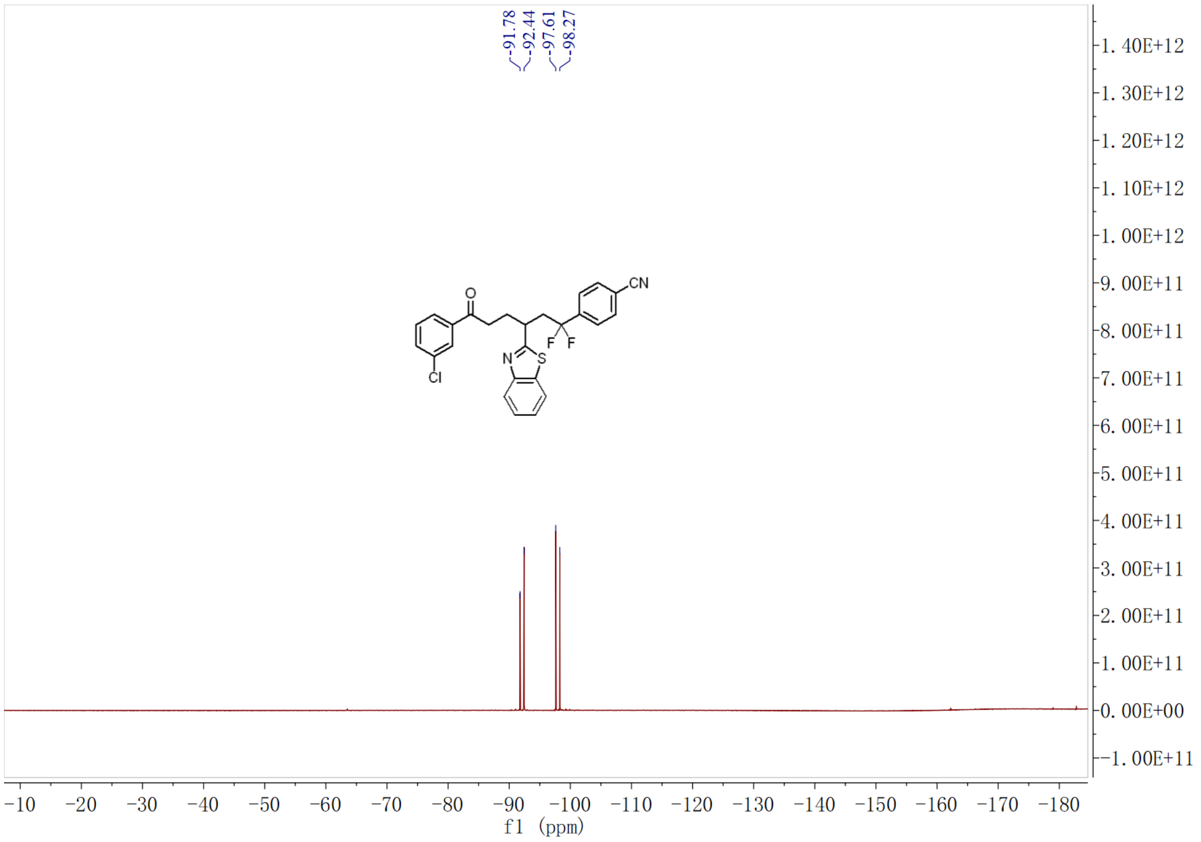

Supplementary Figure 45. 3j ^{13}C NMR (100 MHz, $CDCl_3$)

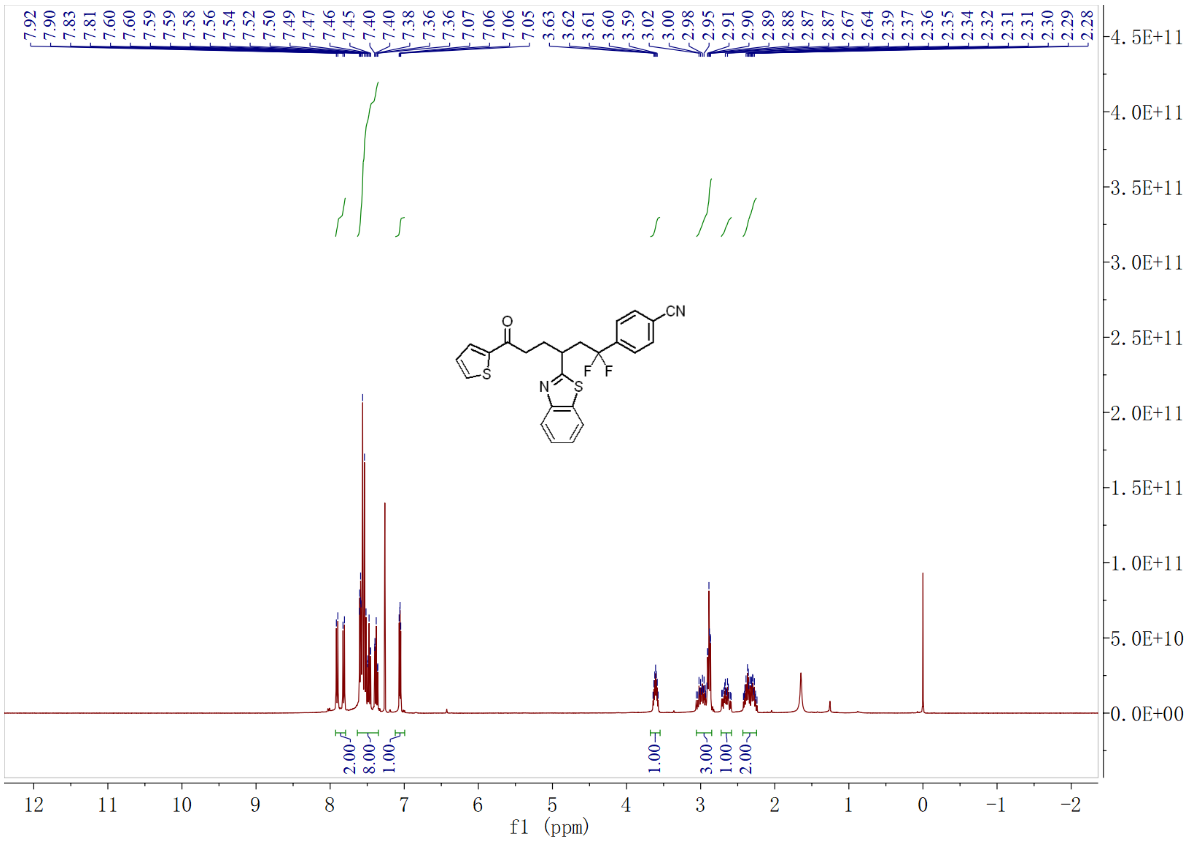

Supplementary Figure 46. 3j ^{19}F NMR (376 MHz, $CDCl_3$)

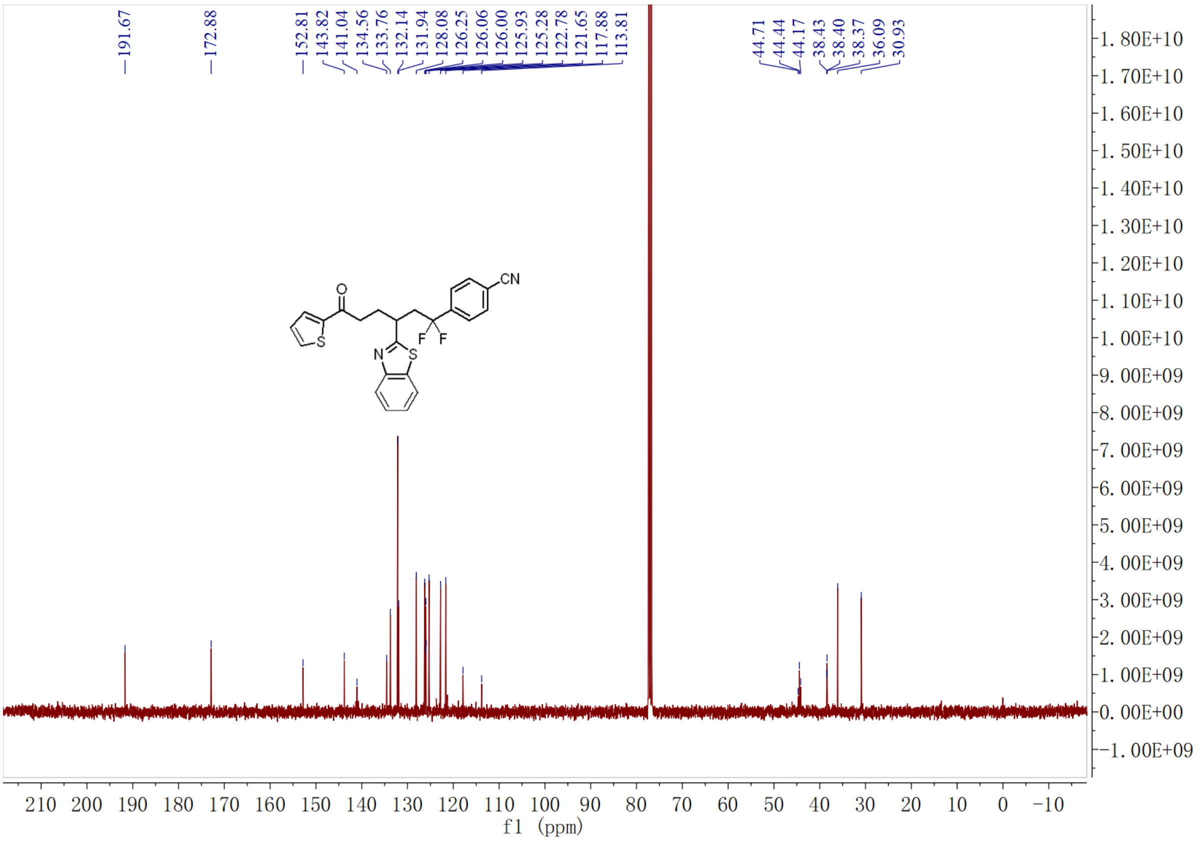

Supplementary Figure 47. 3k ^{1}H NMR (400 MHz, $CDCl_3$)

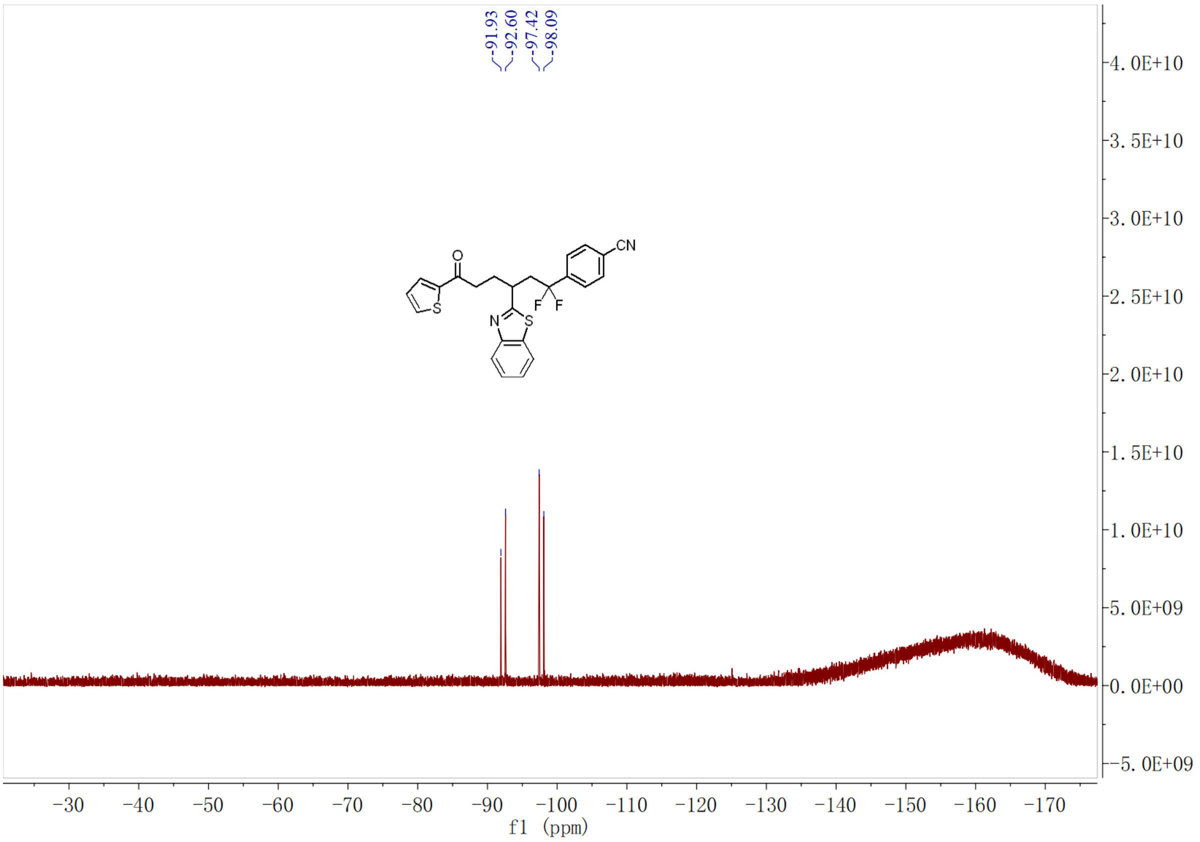

Supplementary Figure 48. 3k ^{13}C NMR (100 MHz, $CDCl_3$)

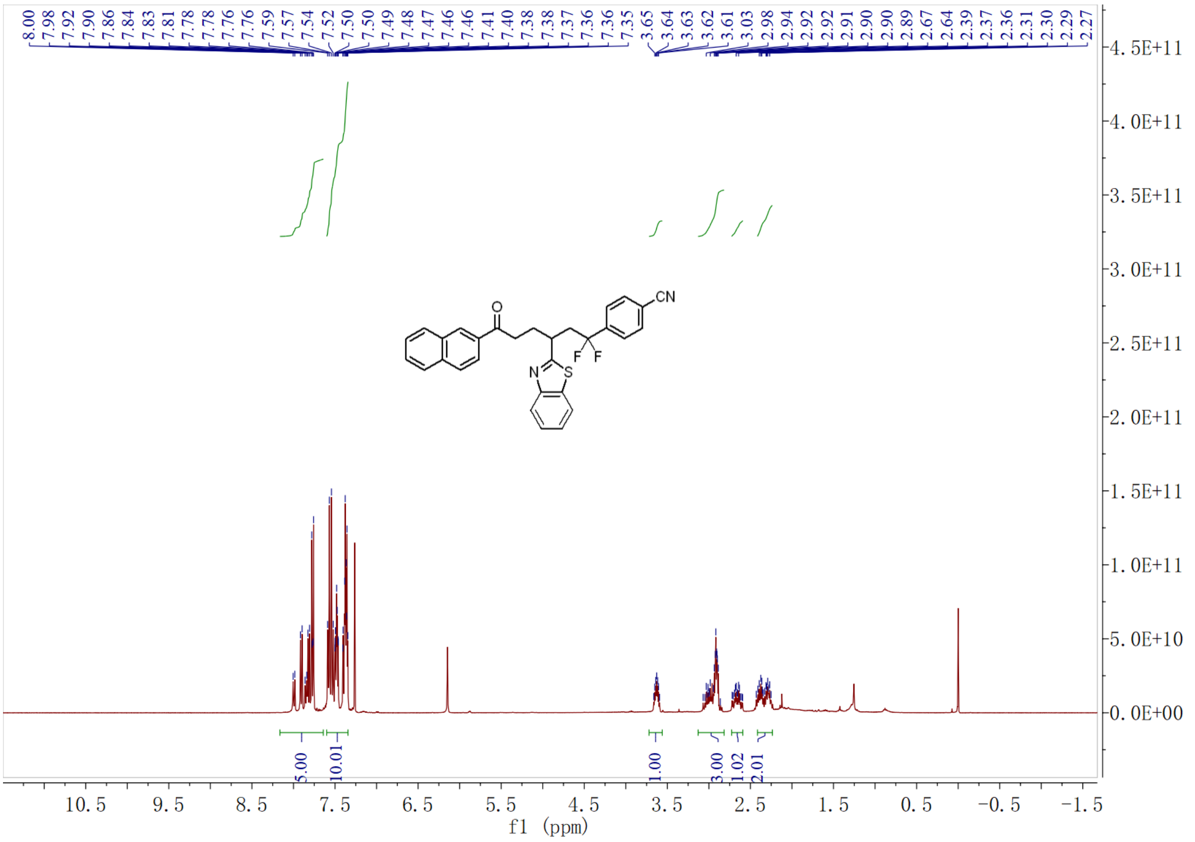

Supplementary Figure 49. 3k ^{19}F NMR (376 MHz, $CDCl_3$)

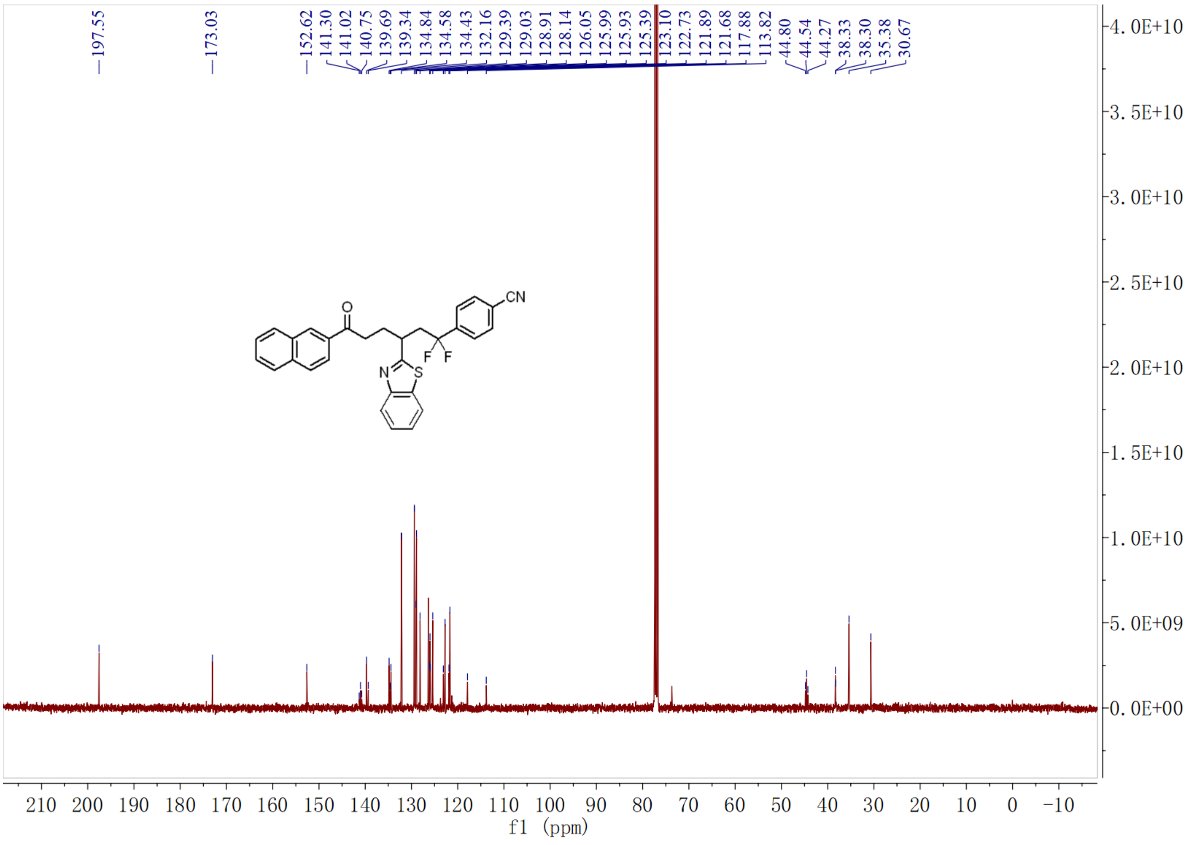

Supplementary Figure 50. 3l ^{1}H NMR (400 MHz, $CDCl_3$)

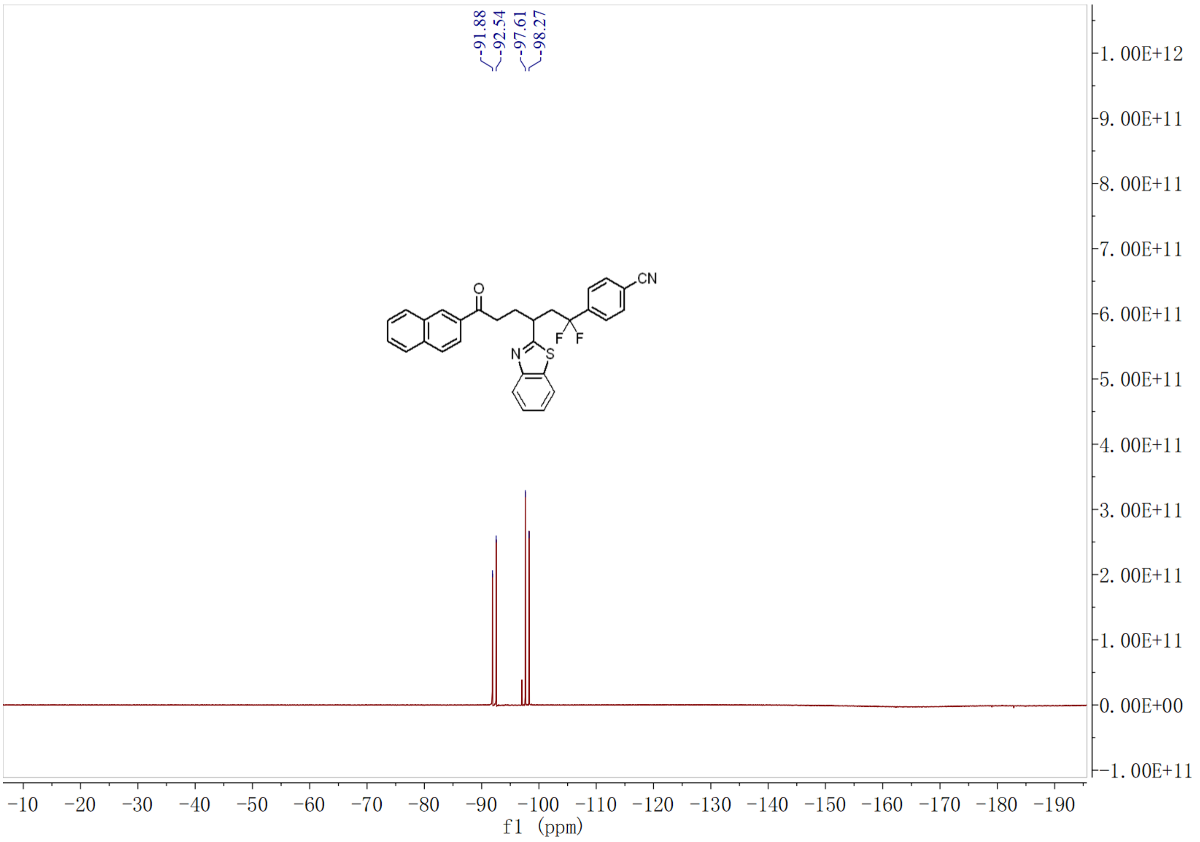

Supplementary Figure 51. 3l ^{13}C NMR (100 MHz, $CDCl_3$)

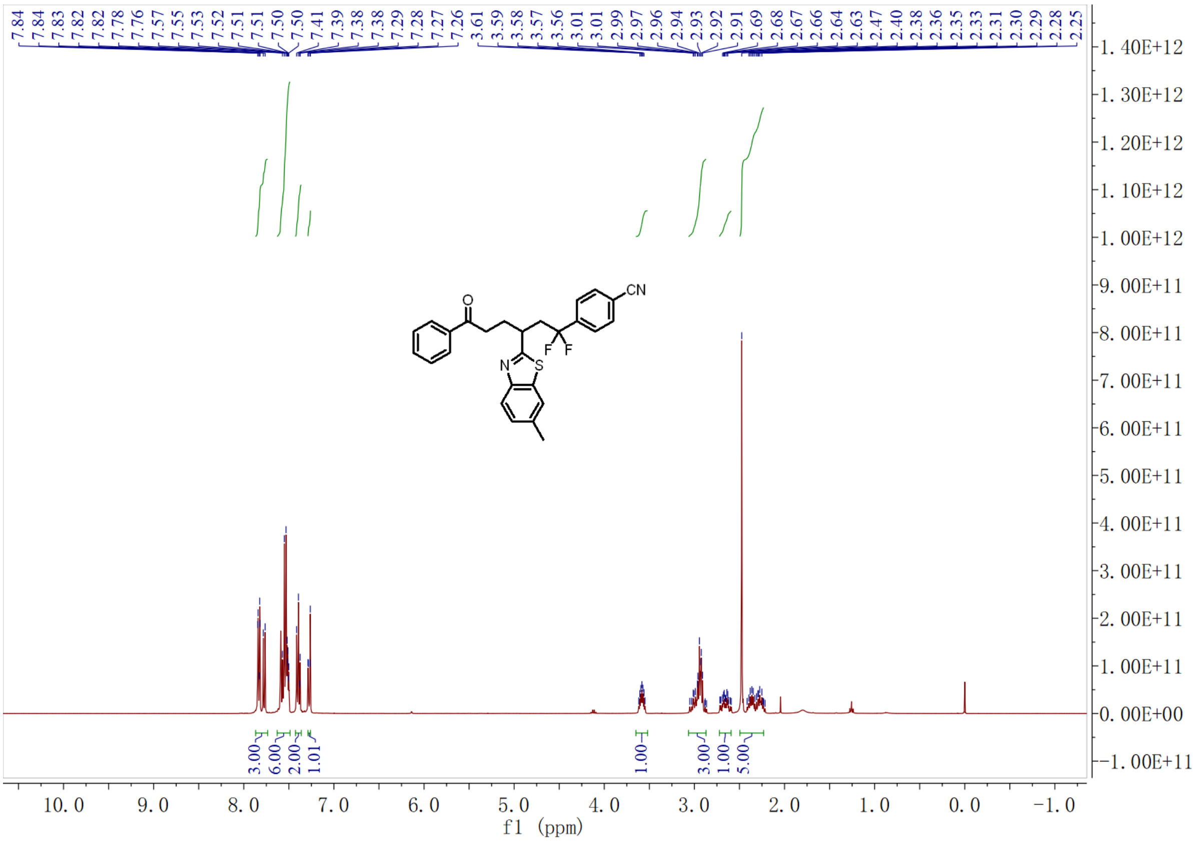

Supplementary Figure 52. 3l ^{19}F NMR (376 MHz, CDCl3)

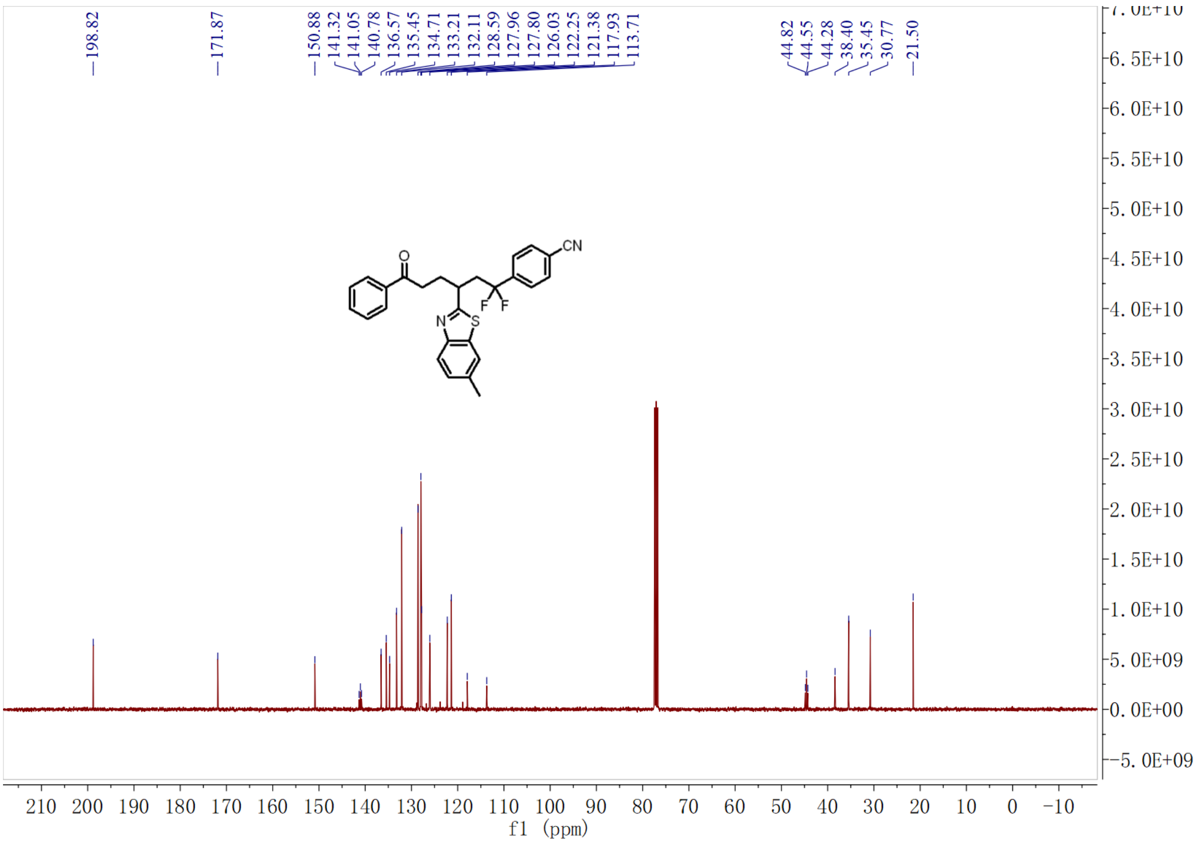

Supplementary Figure 53. 3m ^{1}H NMR (400 MHz, CDCl3)

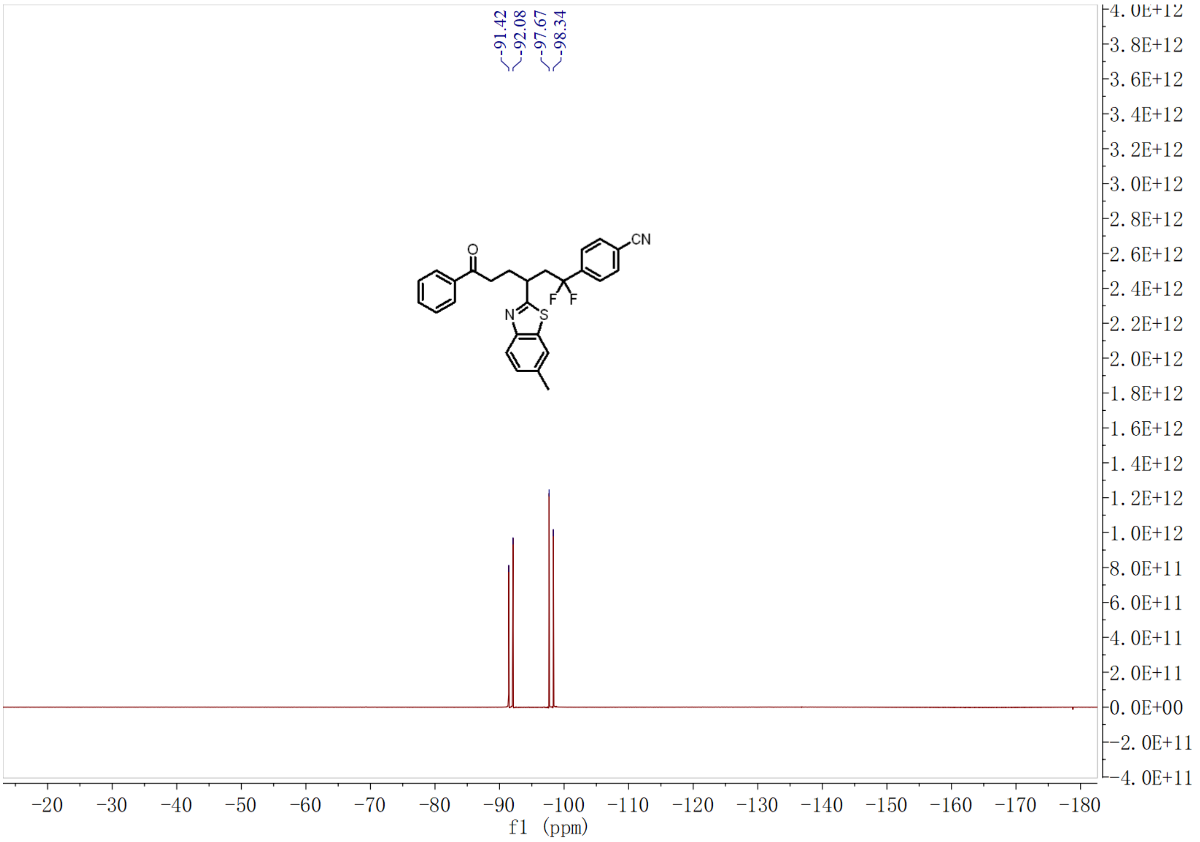

Supplementary Figure 54. 3m ^{13}C NMR (100 MHz, $CDCl_3$)

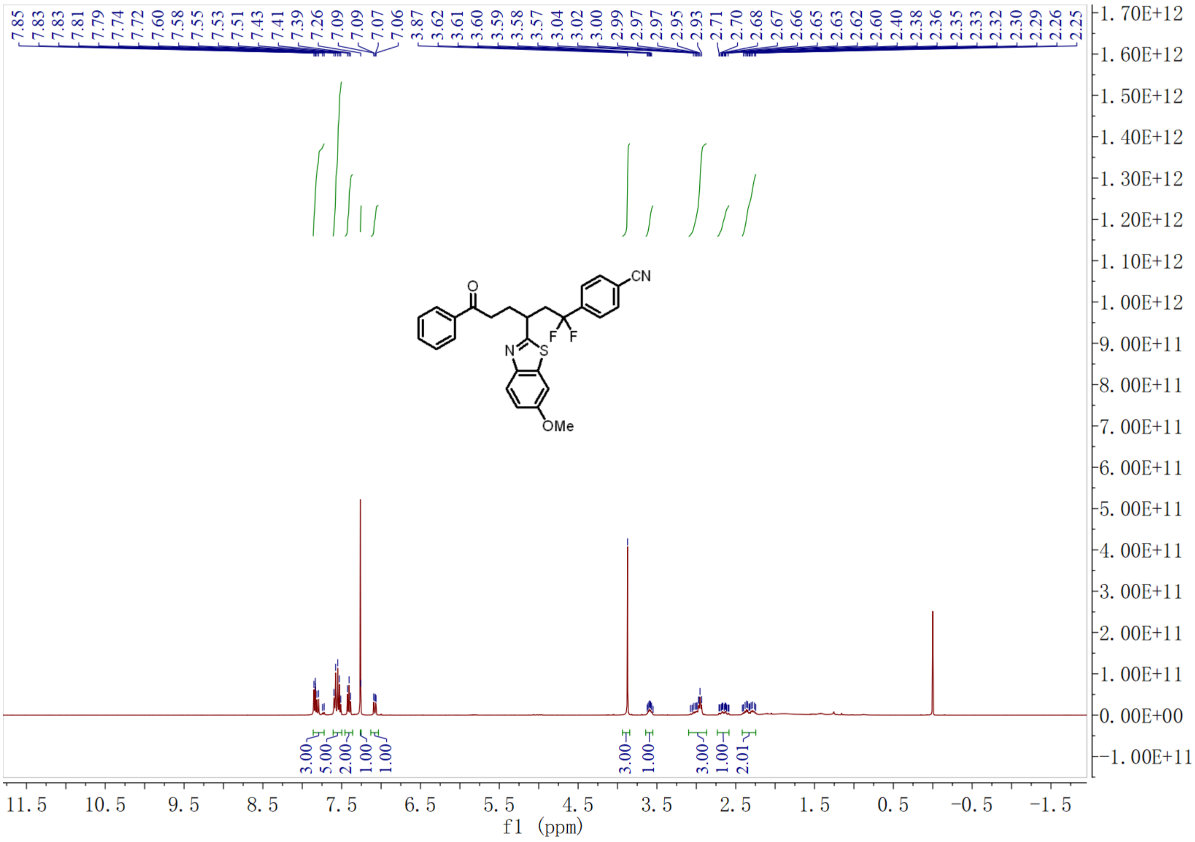

Supplementary Figure 55. 3m ^{19}F NMR (376 MHz, $CDCl_3$)

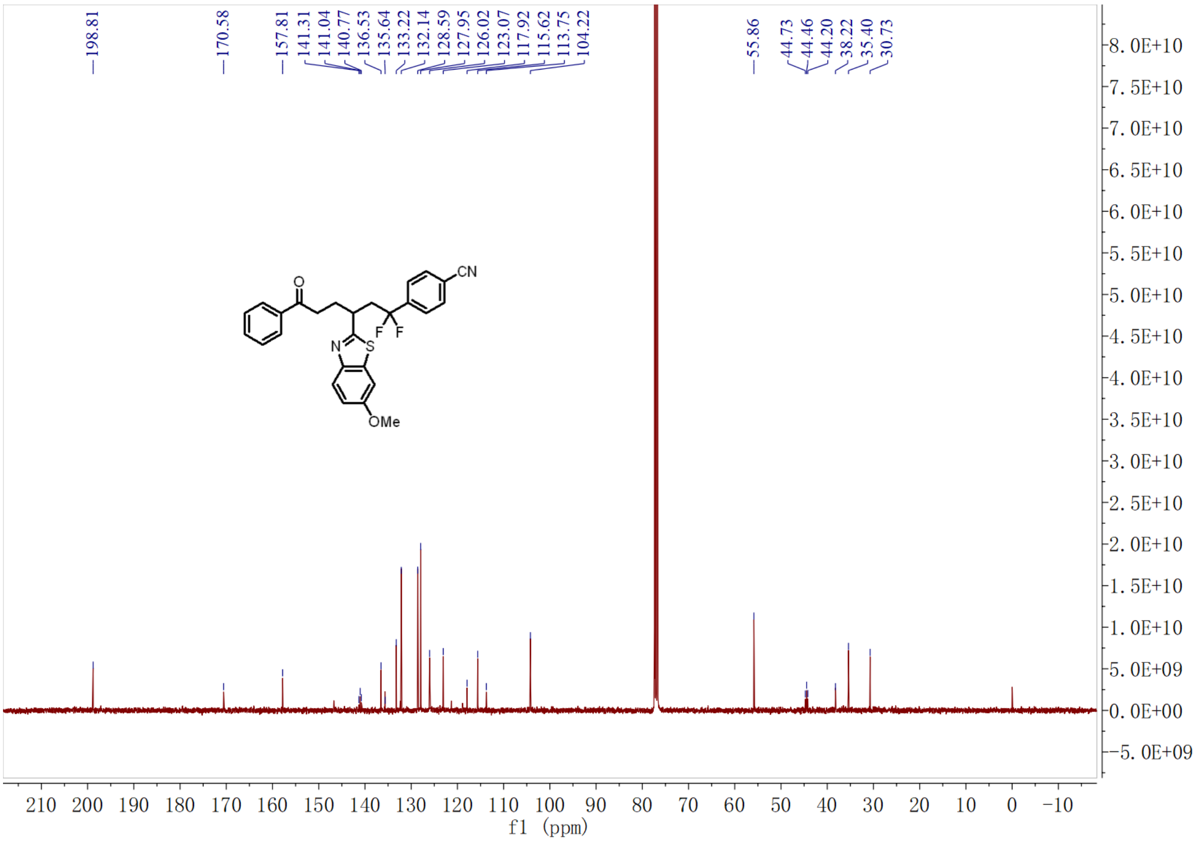

Supplementary Figure 56. 3n ^{1}H NMR (400 MHz, $CDCl_3$)

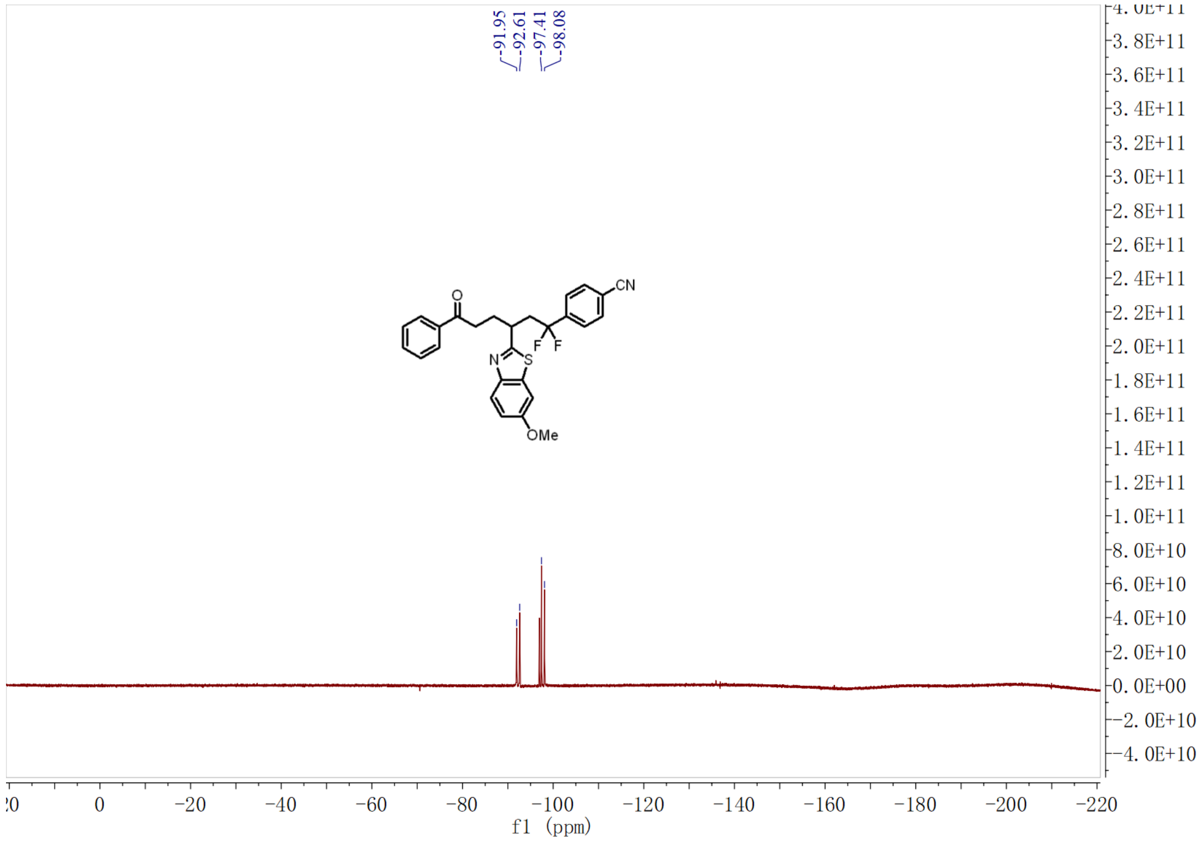

Supplementary Figure 57. 3n ^{13}C NMR (100 MHz, $CDCl_3$)

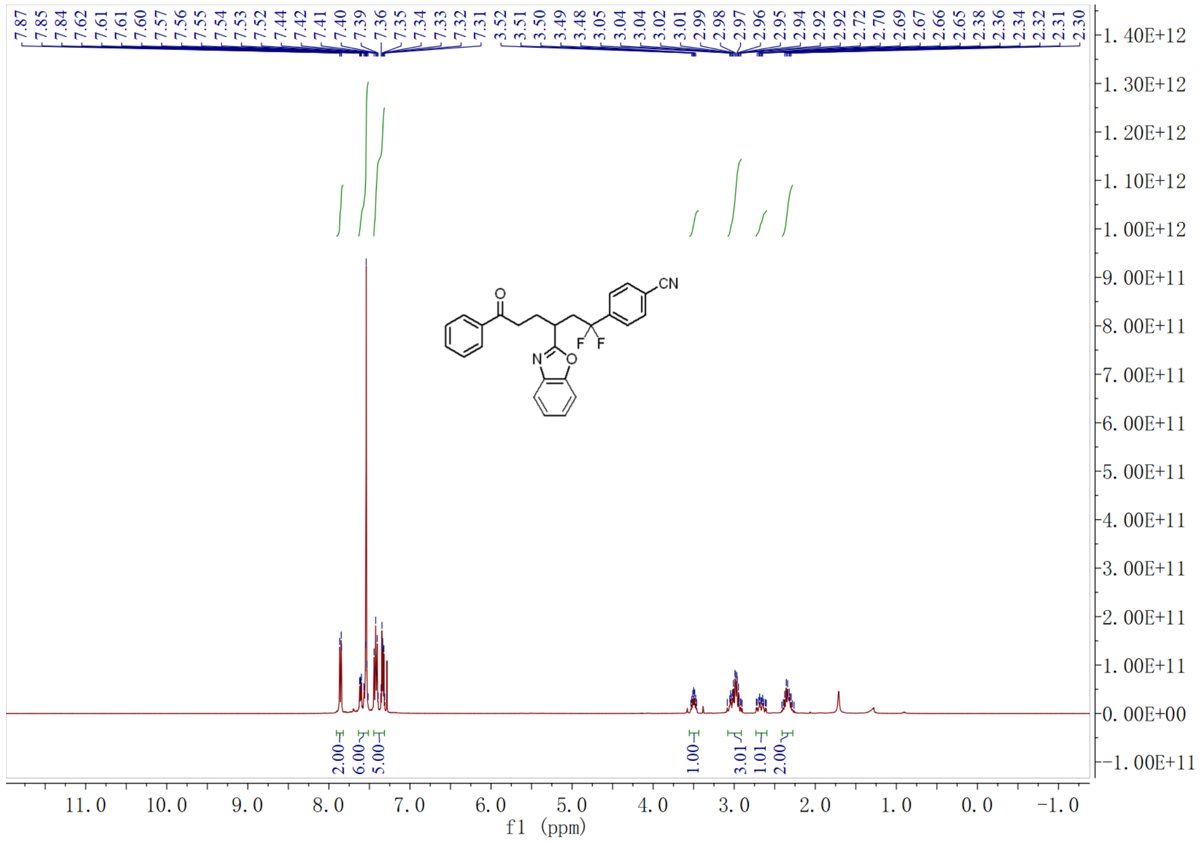

Supplementary Figure 58. 3n ^{19}F NMR (376 MHz, $CDCl_3$)

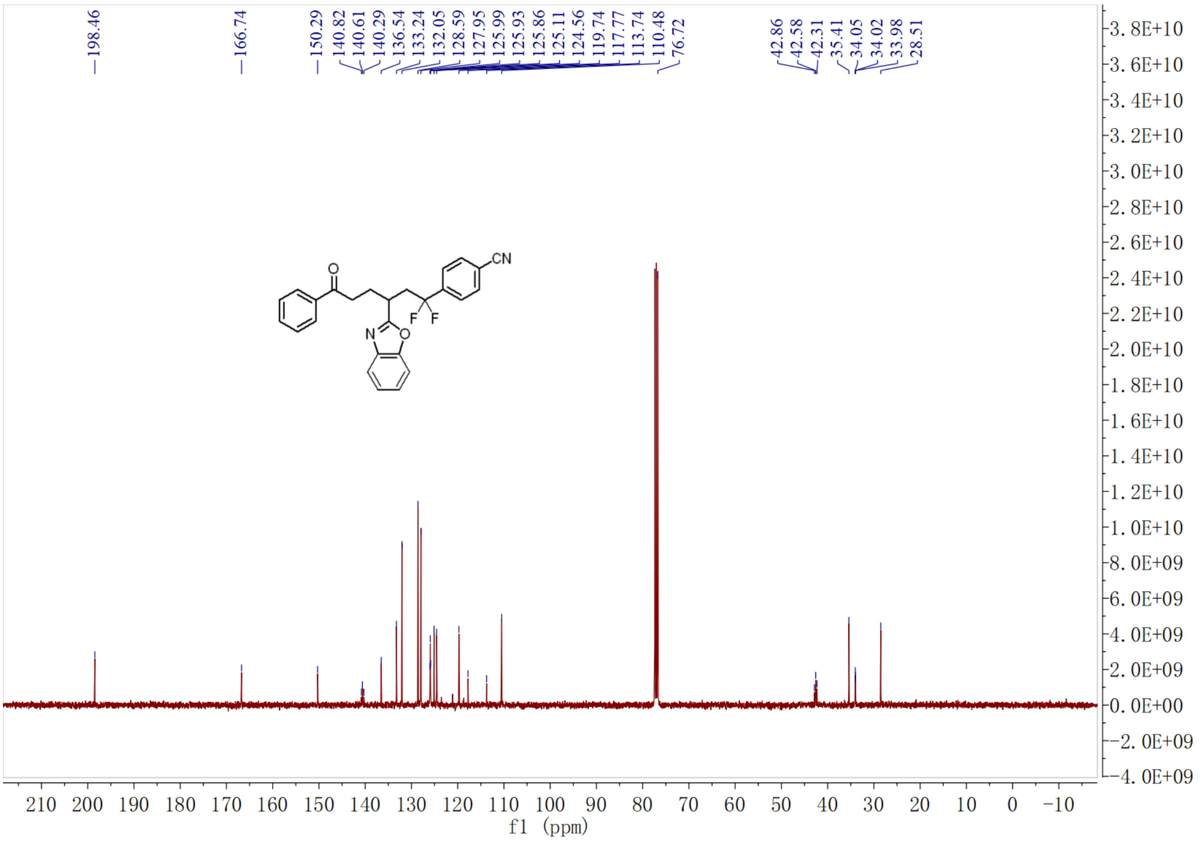

Supplementary Figure 59. 3o ^{1}H NMR (400 MHz, $CDCl_3$)

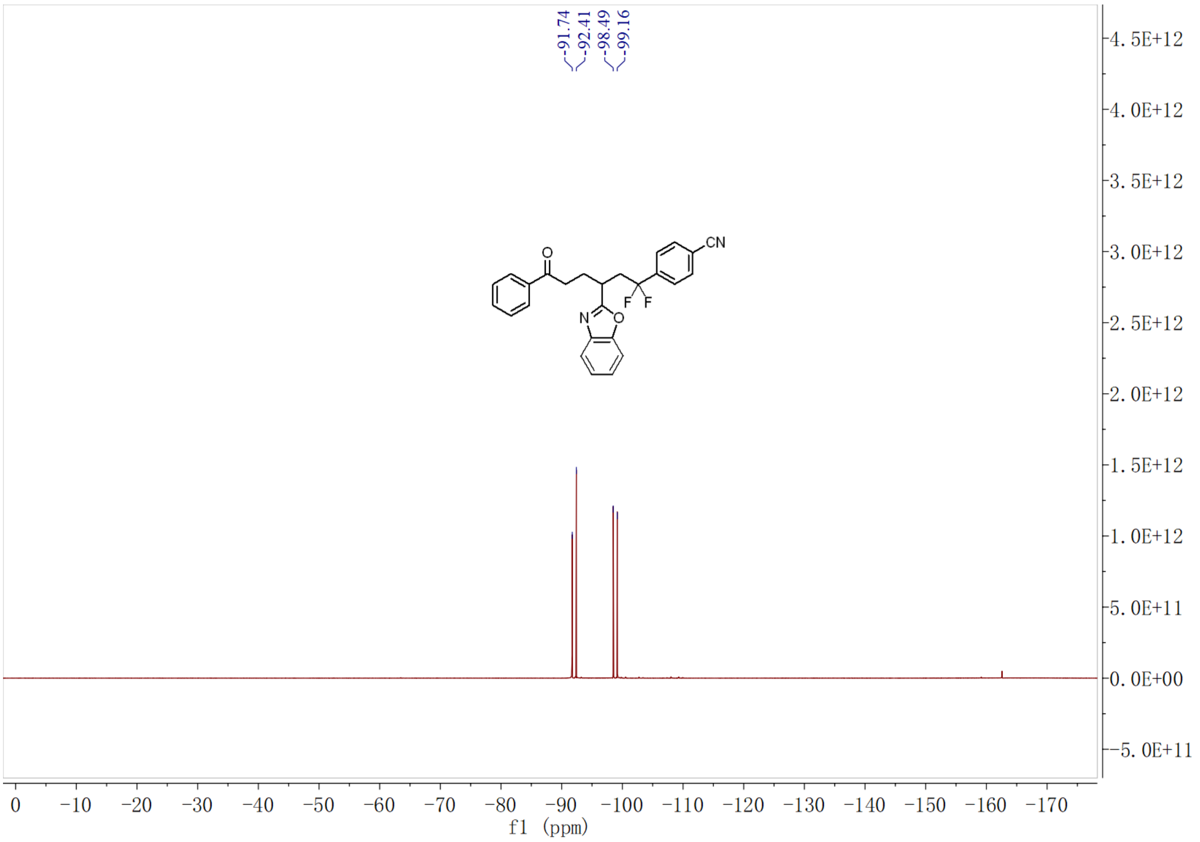

Supplementary Figure 60. 3o ^{13}C NMR (100 MHz, $CDCl_3$)

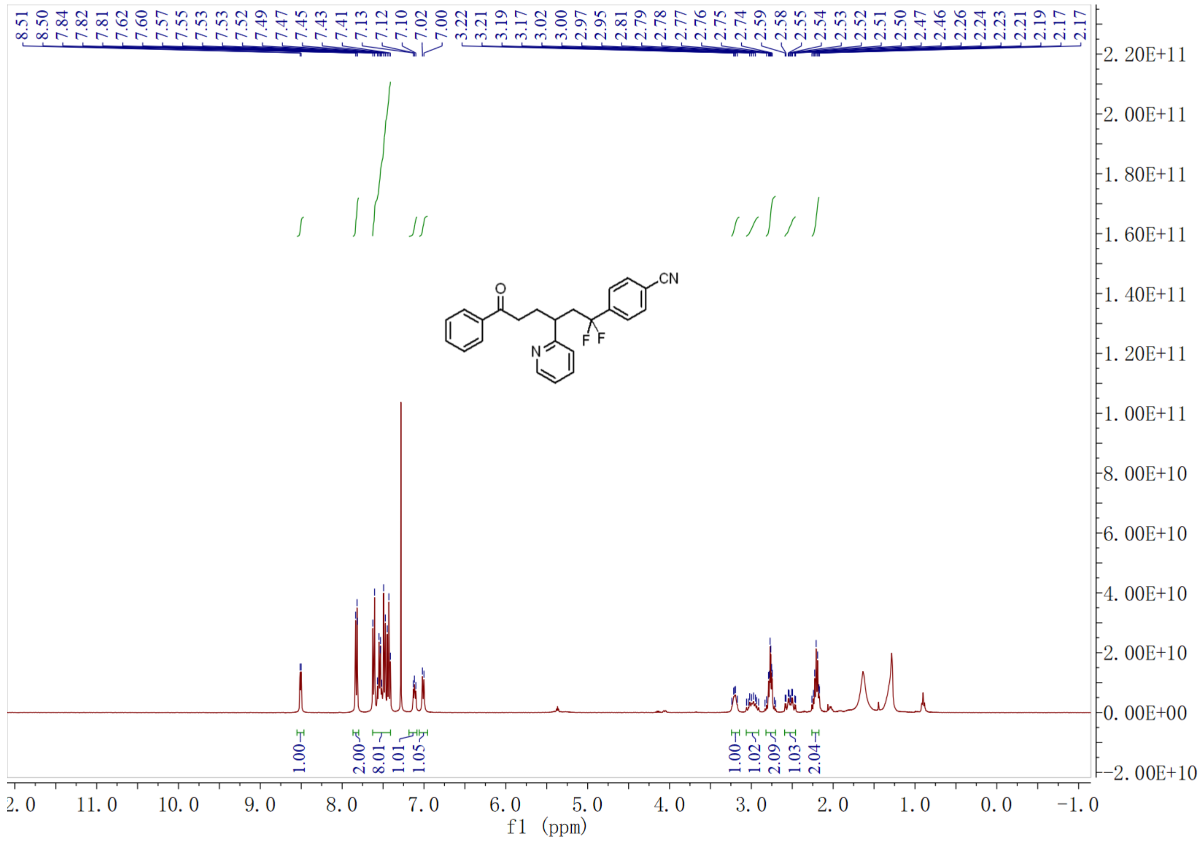

Supplementary Figure 61. 3o ^{19}F NMR (376 MHz, $CDCl_3$)

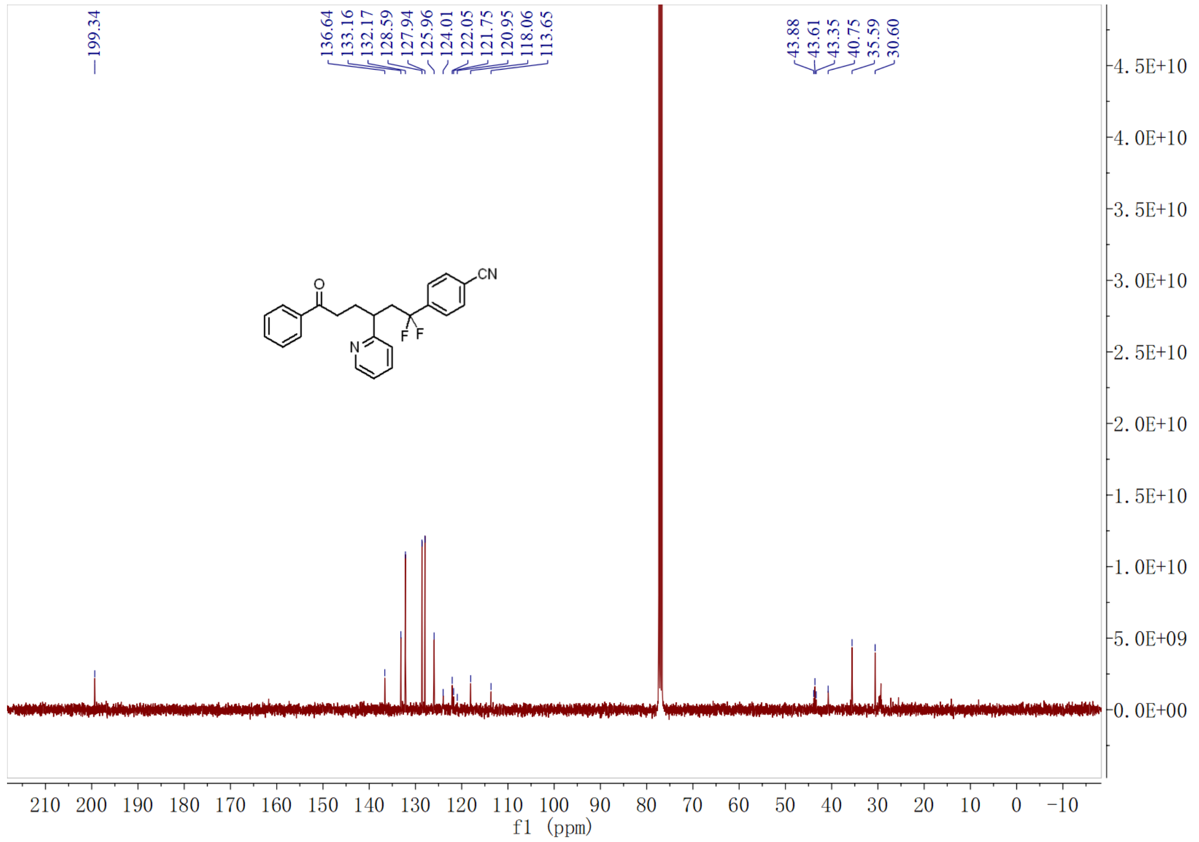

Supplementary Figure 62. 3p ^{1}H NMR (400 MHz, $CDCl_3$)

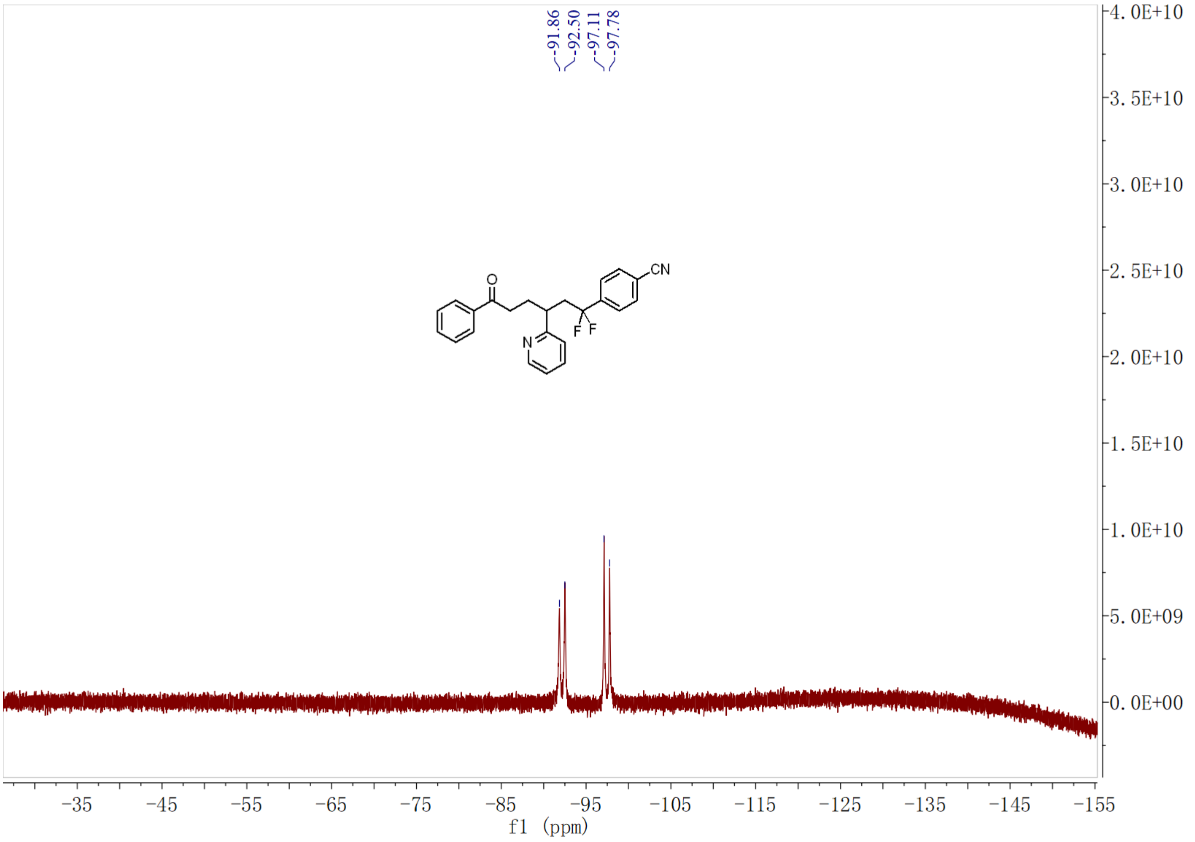

Supplementary Figure 63. 3p ^{13}C NMR (100 MHz, $CDCl_3$)

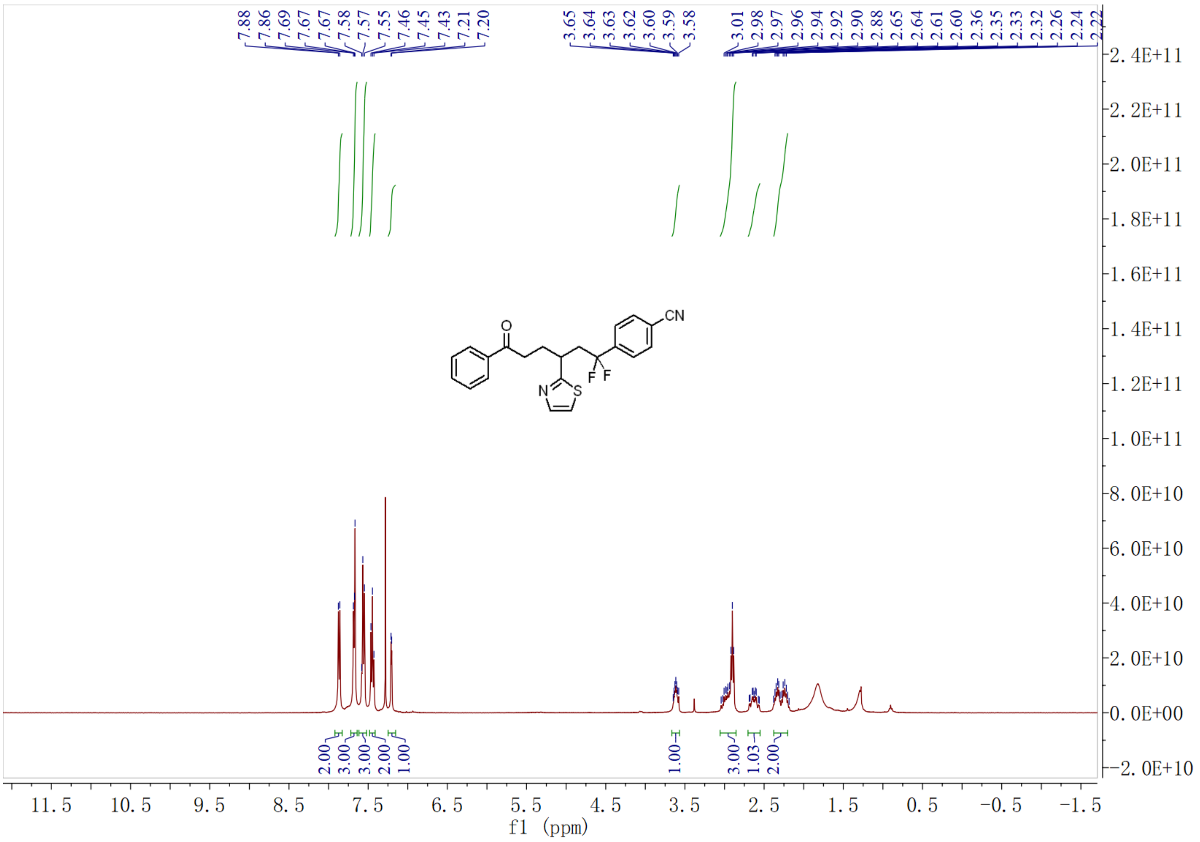

Supplementary Figure 64. 3p ^{19}F NMR (376 MHz, $CDCl_3$)

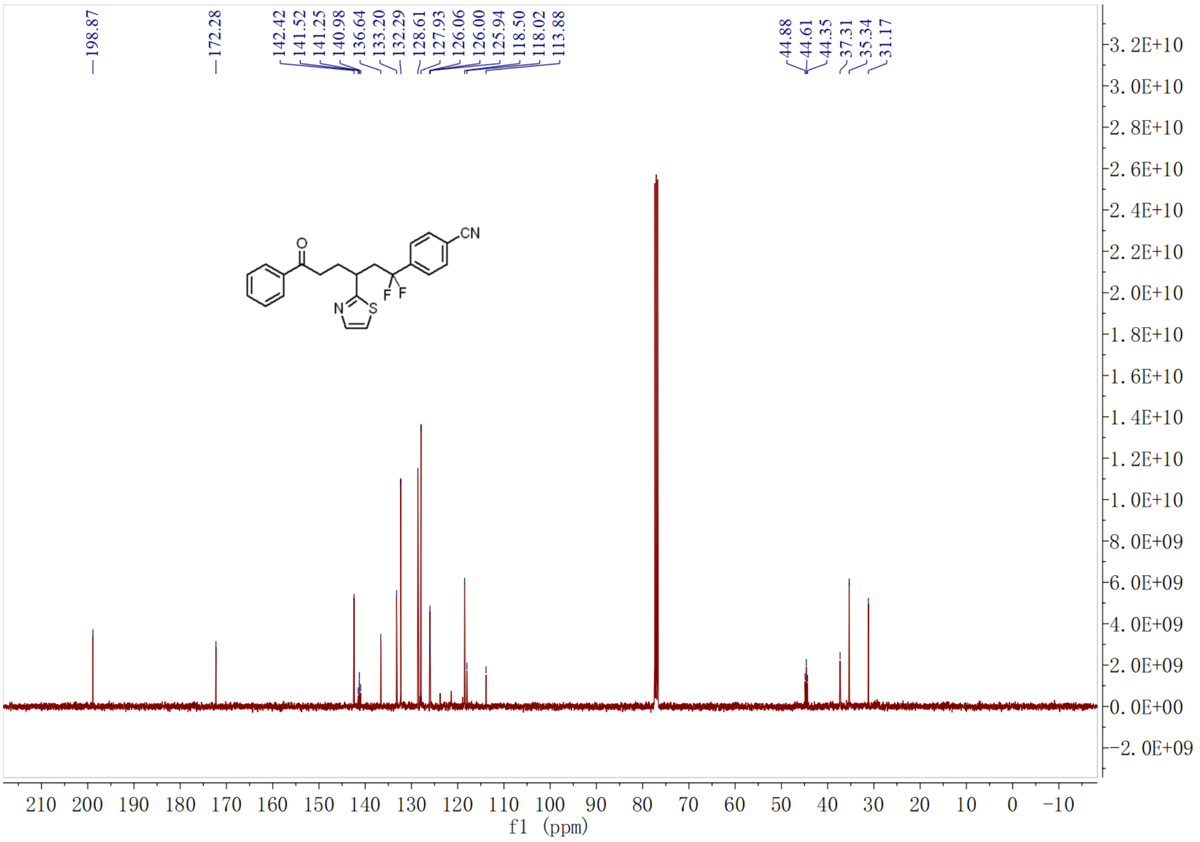

Supplementary Figure 65. 3q ^{1}H NMR (400 MHz, $CDCl_3$)

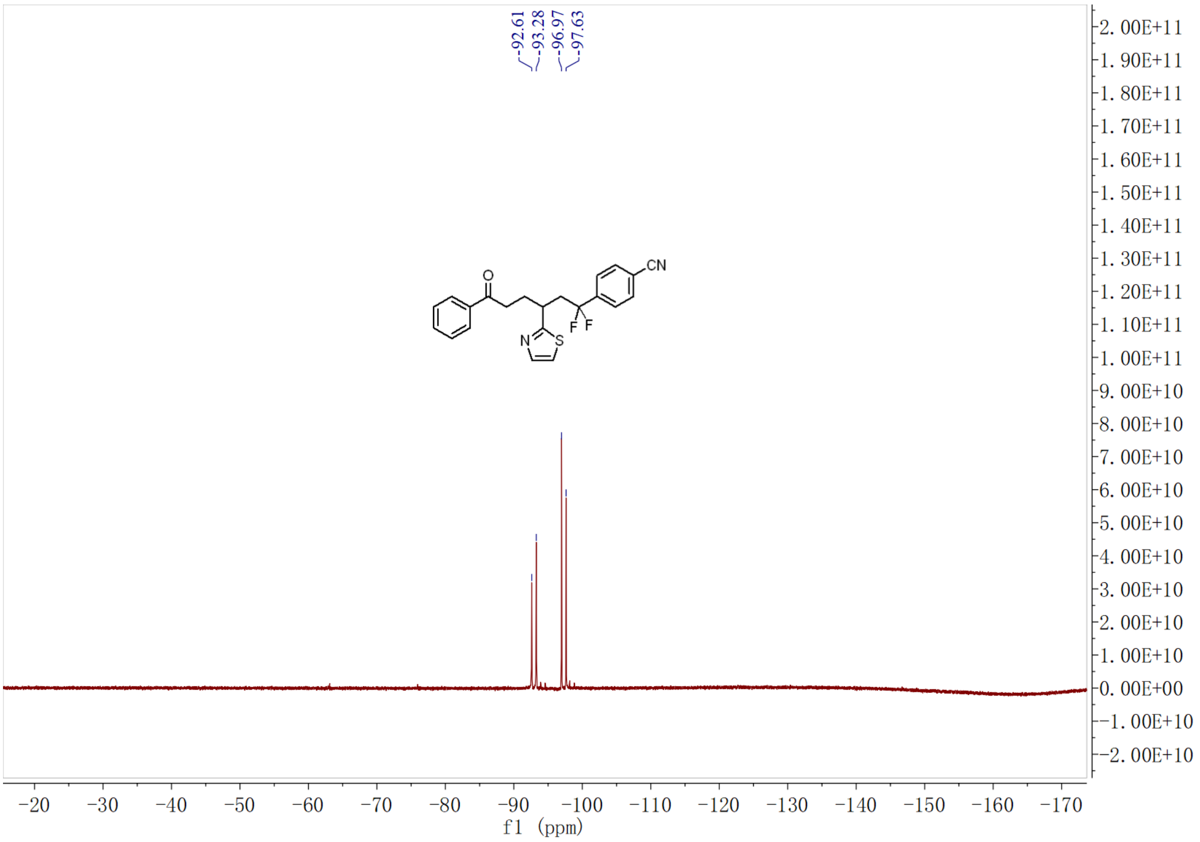

Supplementary Figure 66. 3q ^{13}C NMR (100 MHz, $CDCl_3$)

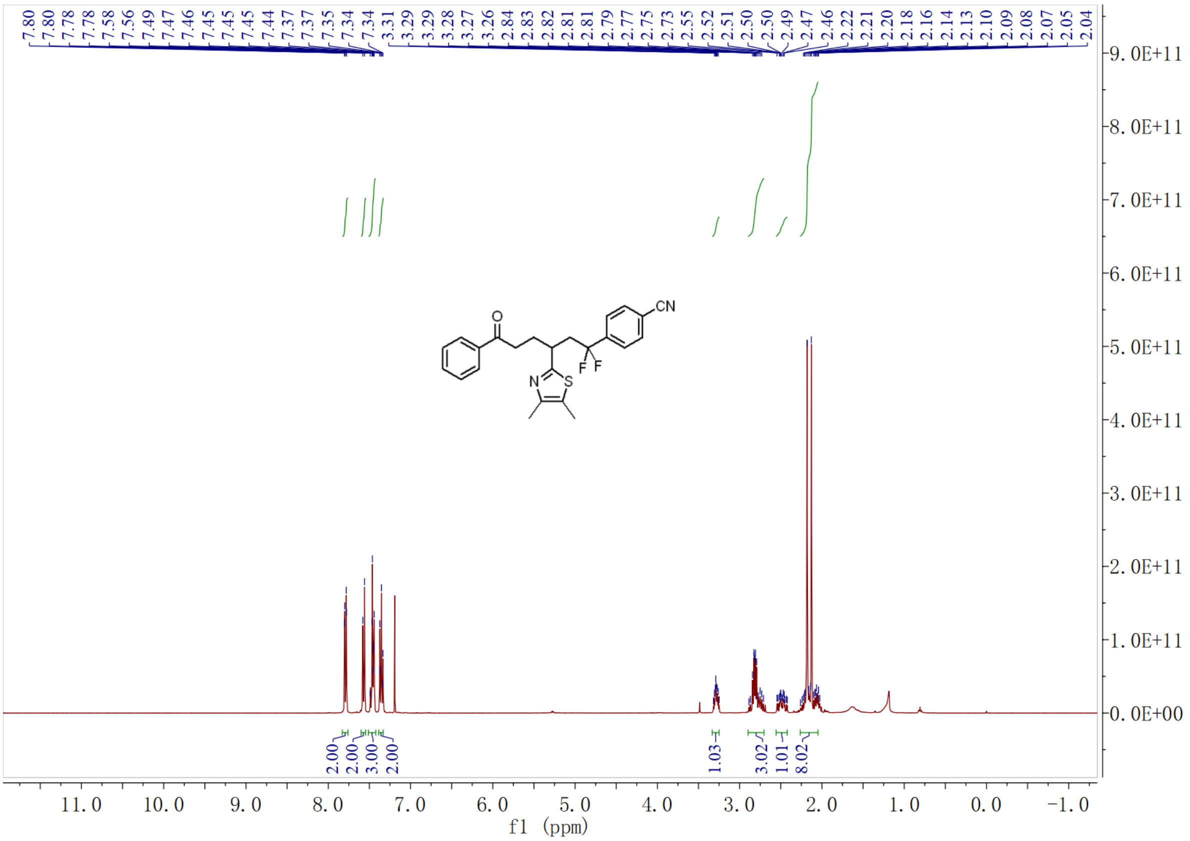

Supplementary Figure 67. 3q ^{19}F NMR (376 MHz, $CDCl_3$)

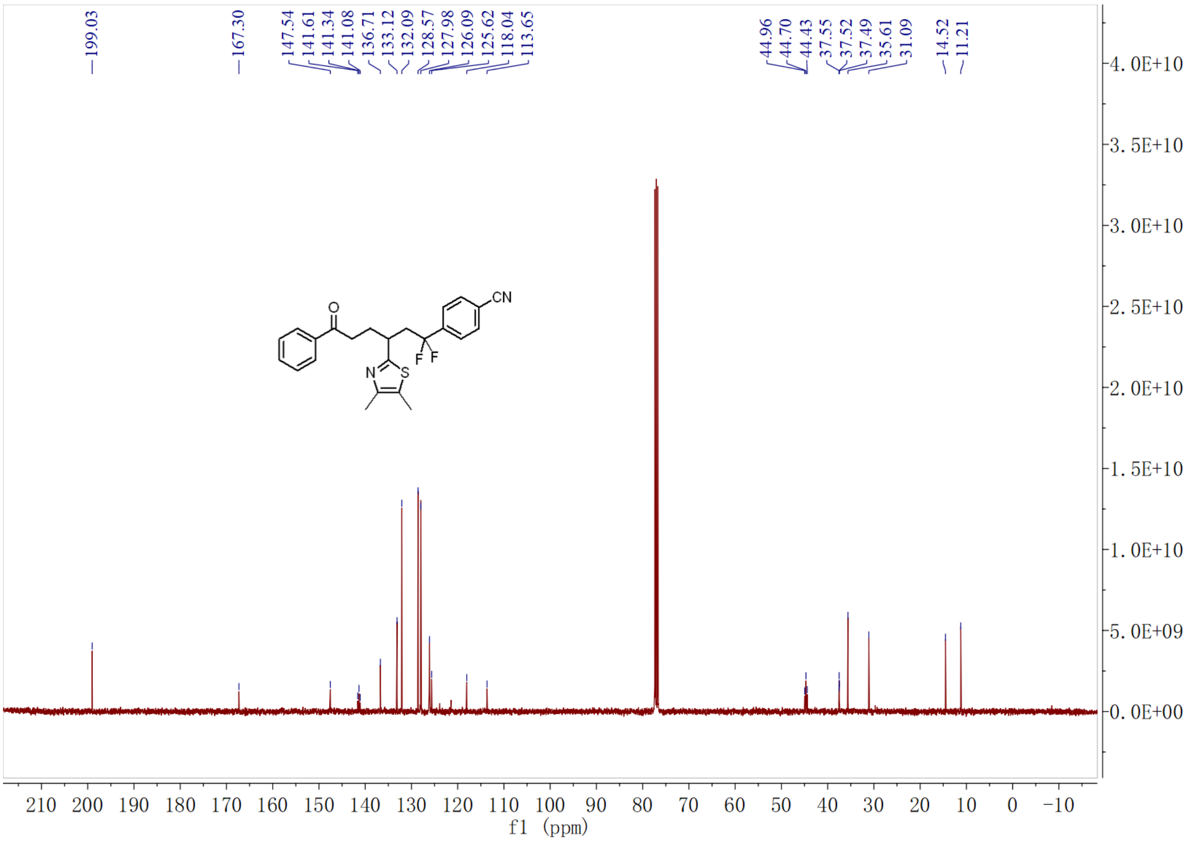

Supplementary Figure 68. 3r ^{1}H NMR (400 MHz, $CDCl_3$)

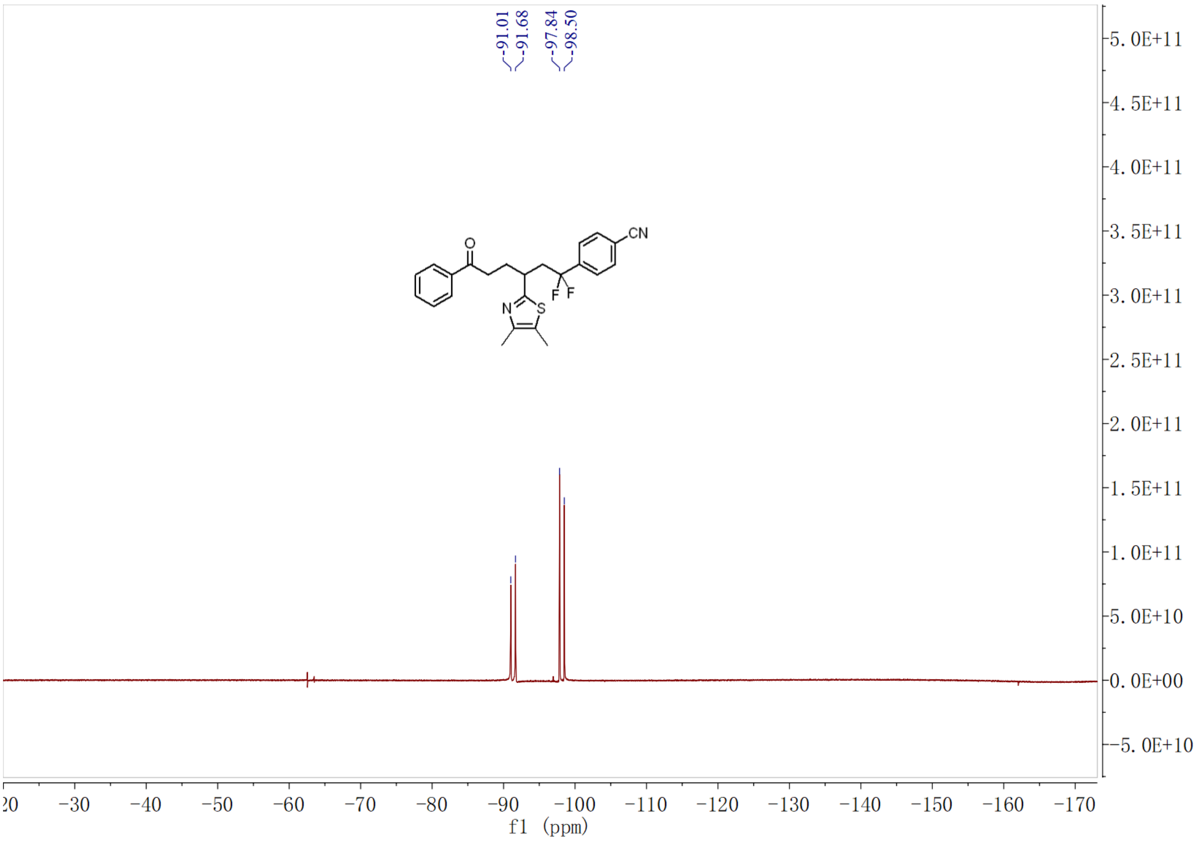

Supplementary Figure 69. 3r ^{13}C NMR (100 MHz, $CDCl_3$)

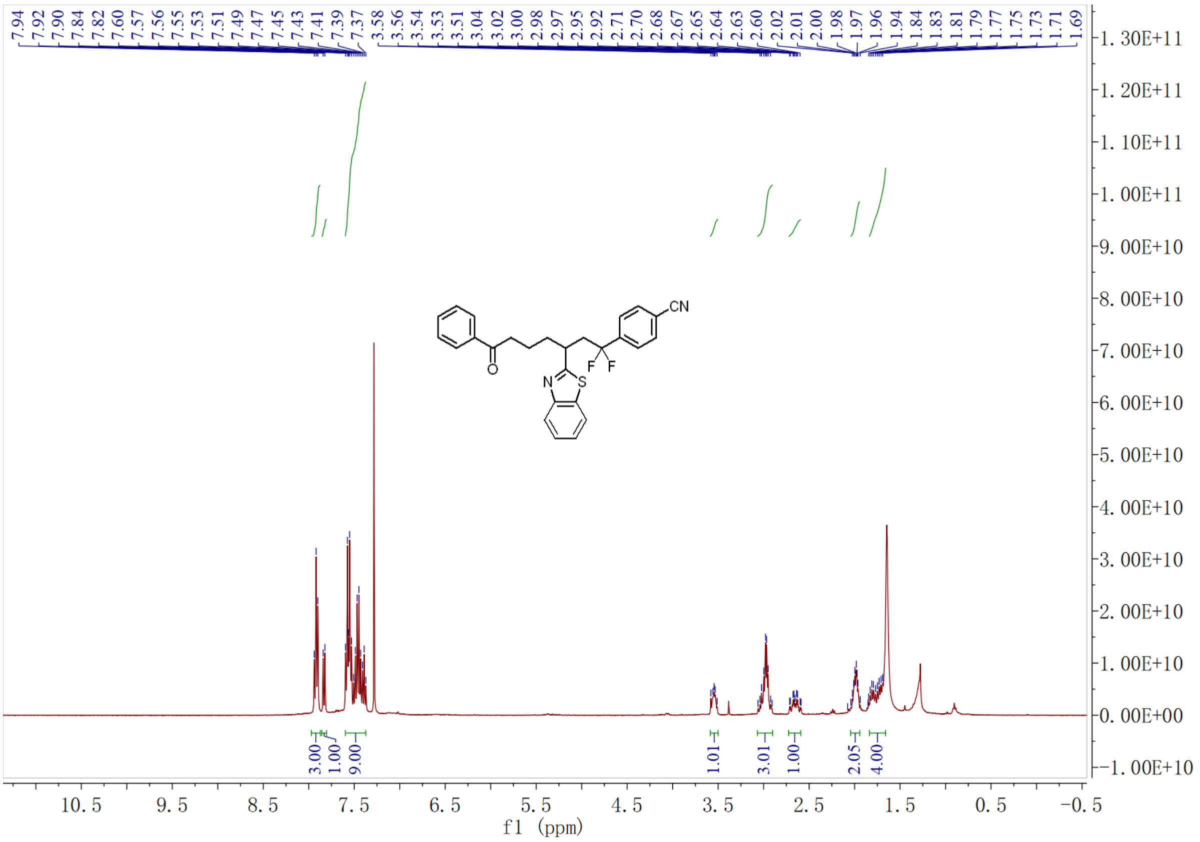

Supplementary Figure 70. 3r ^{19}F NMR (376 MHz, $CDCl_3$)

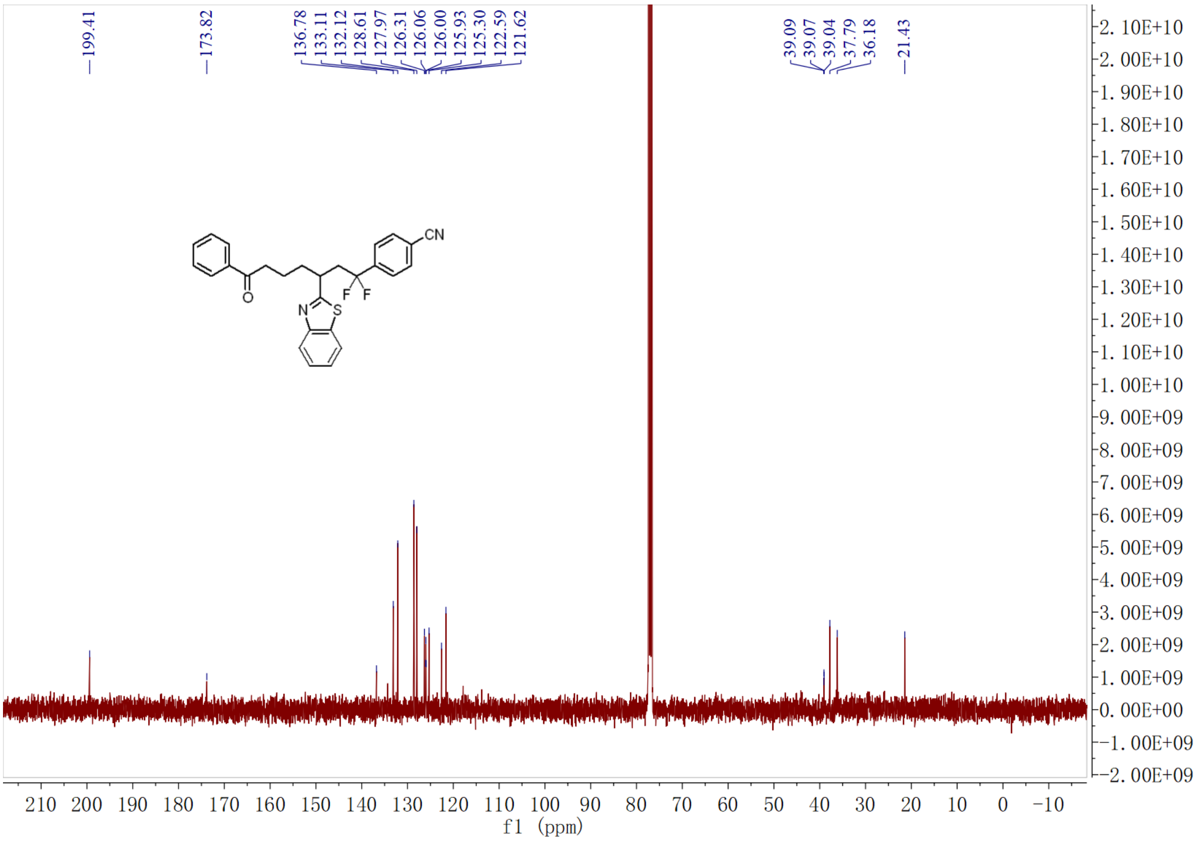

Supplementary Figure 71. 3t ^{1}H NMR (400 MHz, $CDCl_3$)

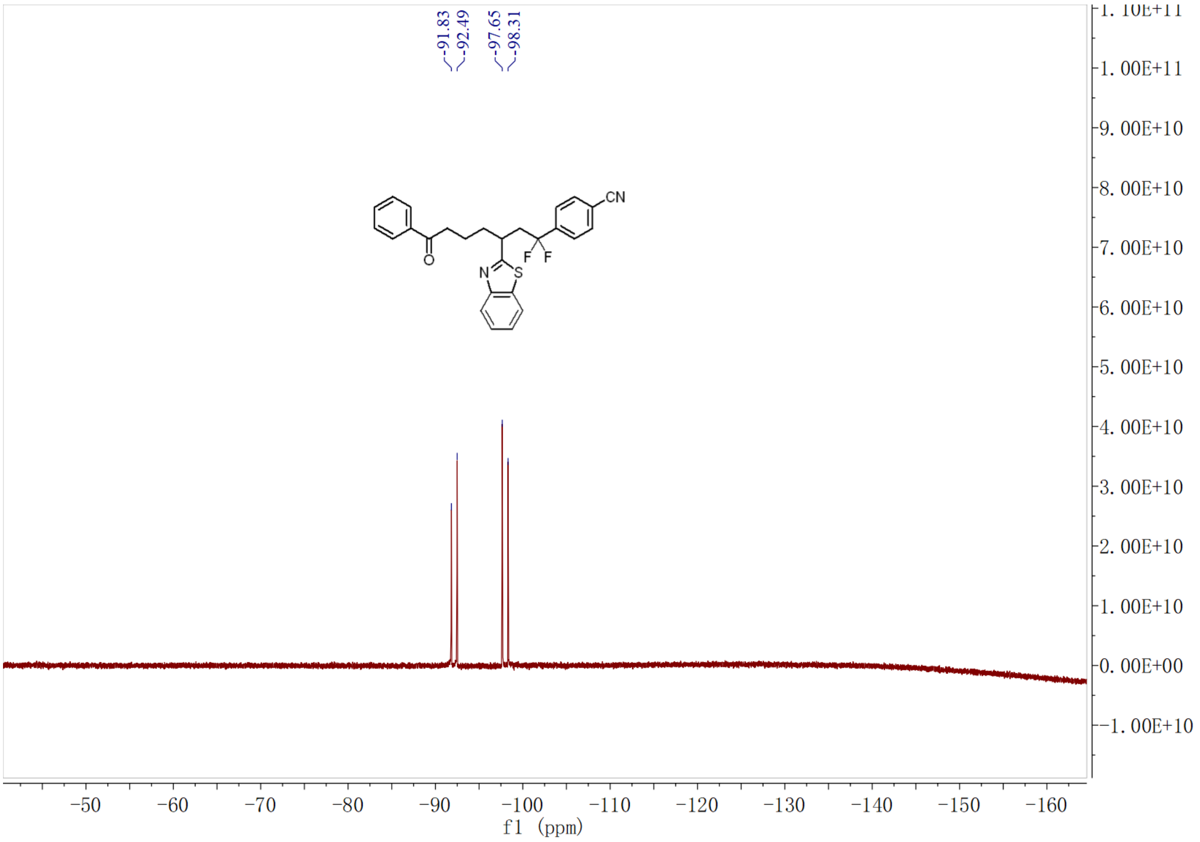

Supplementary Figure 72. 3t ^{13}C NMR (100 MHz, $CDCl_3$)

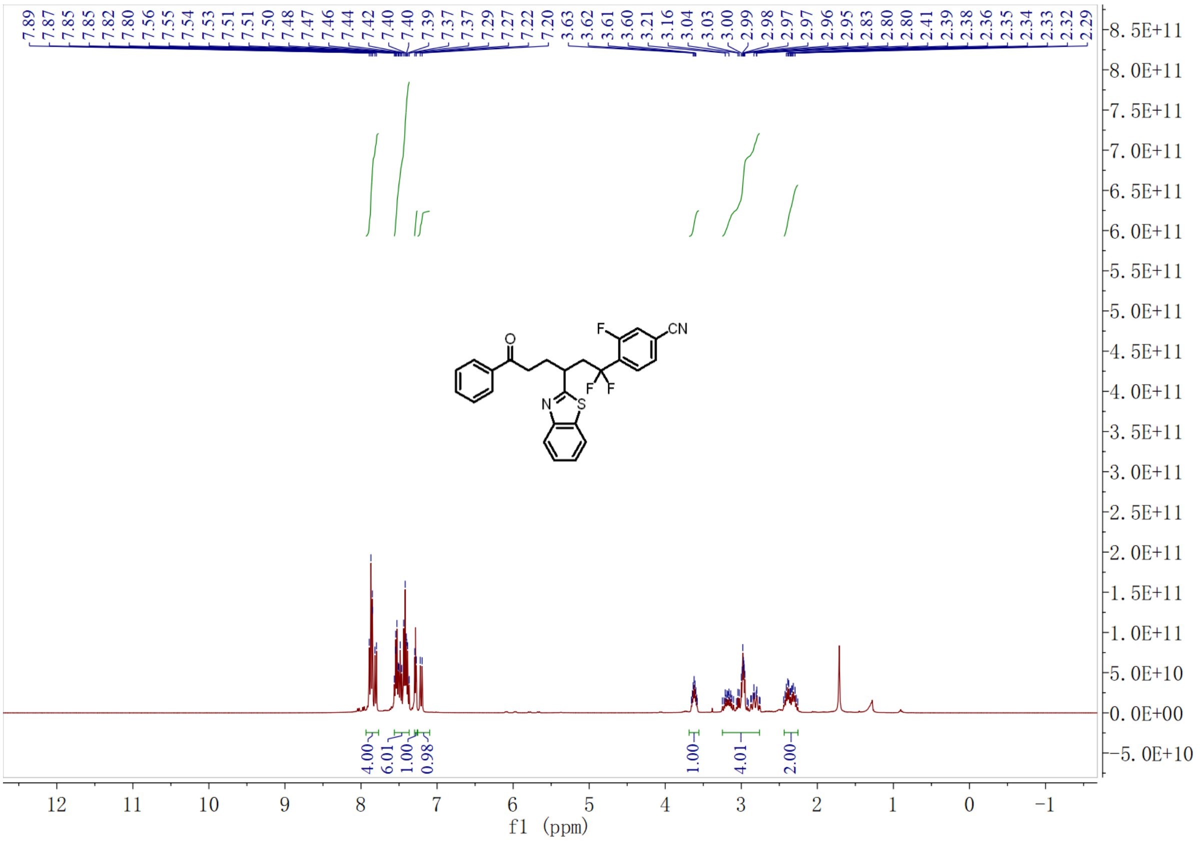

Supplementary Figure 73. 3t ^{19}F NMR (376 MHz, $CDCl_3$)

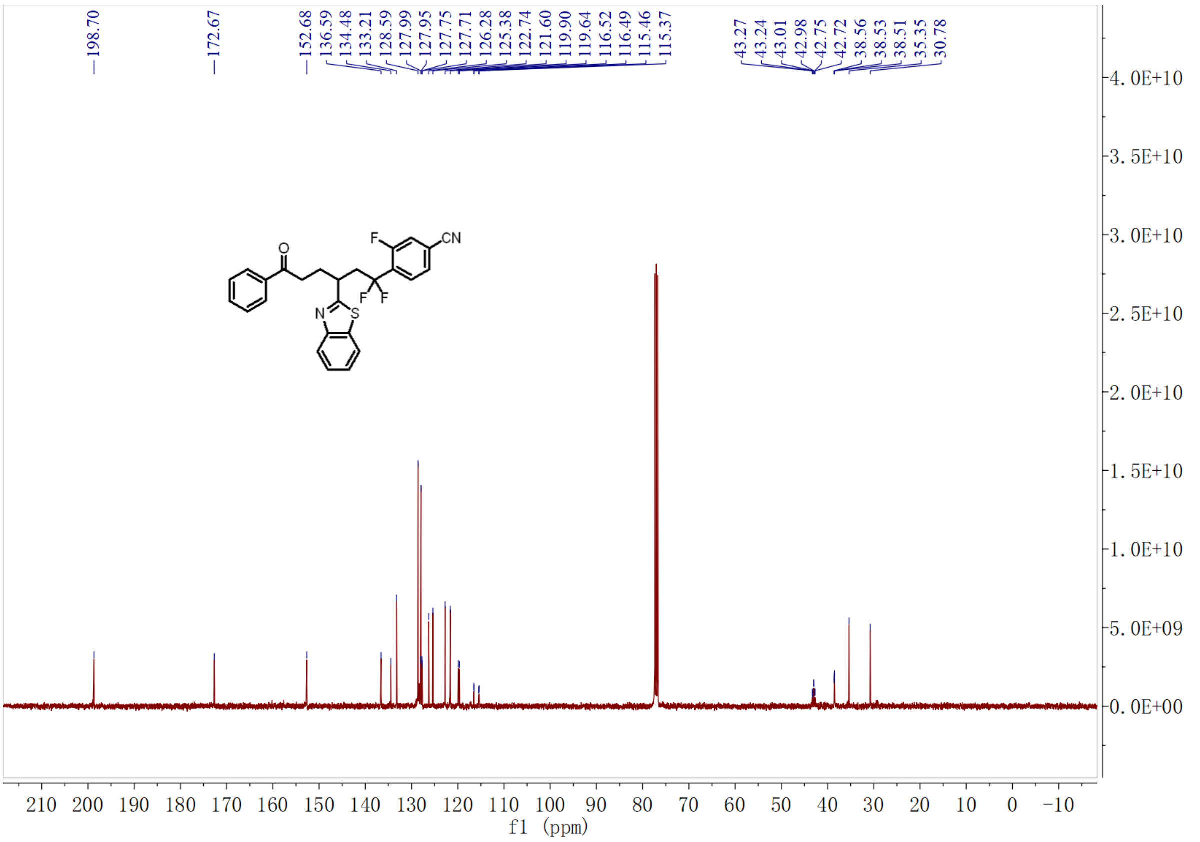

Supplementary Figure 74. 4a ^{1}H NMR (400 MHz, $CDCl_3$)

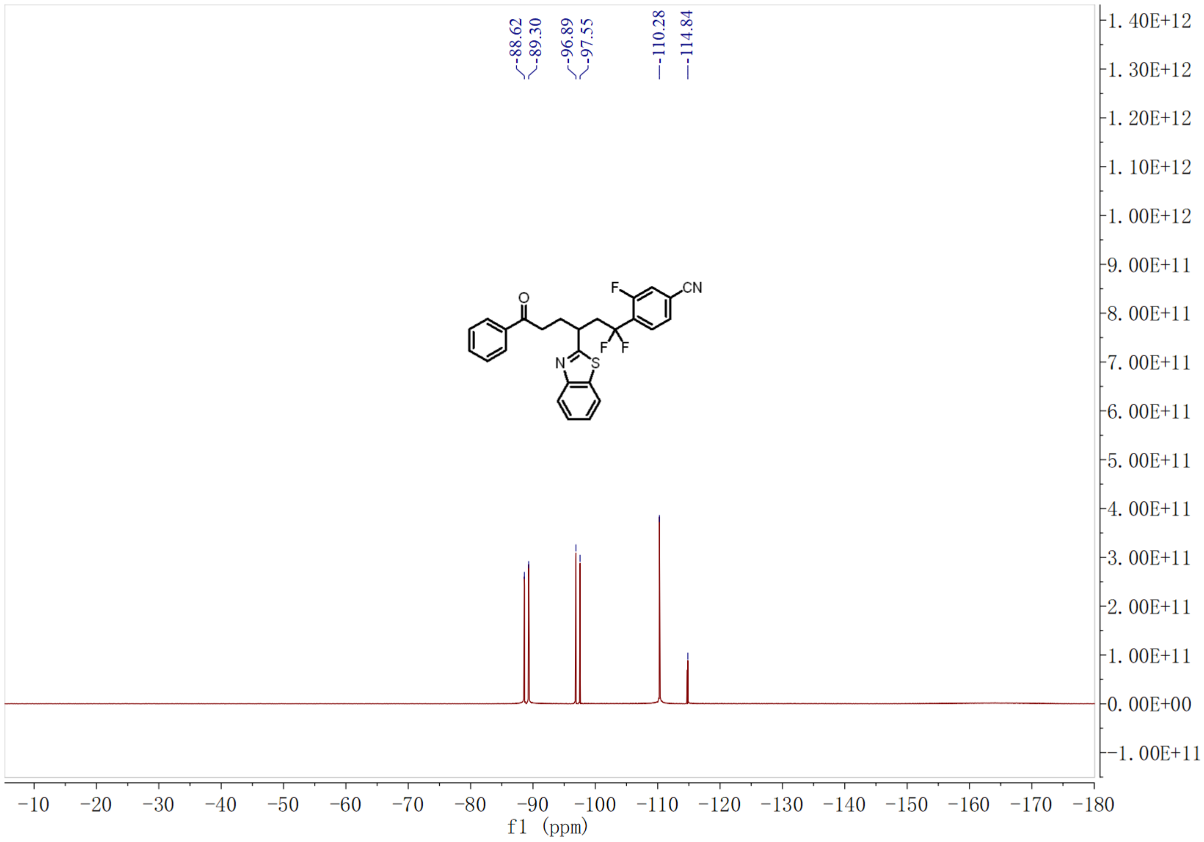

Supplementary Figure 75. 4a ^{13}C NMR (100 MHz, $CDCl_3$)

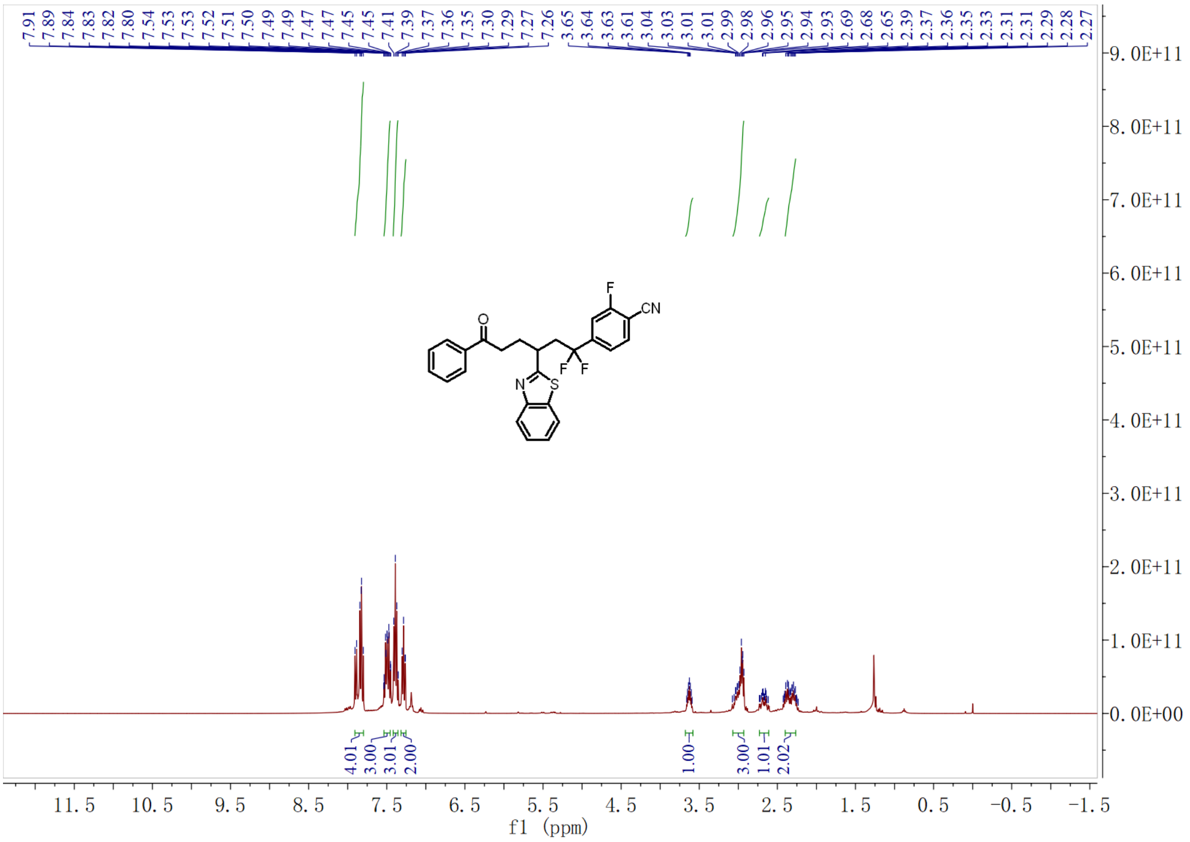

Supplementary Figure 76. 4a ^{19}F NMR (376 MHz, $CDCl_3$)

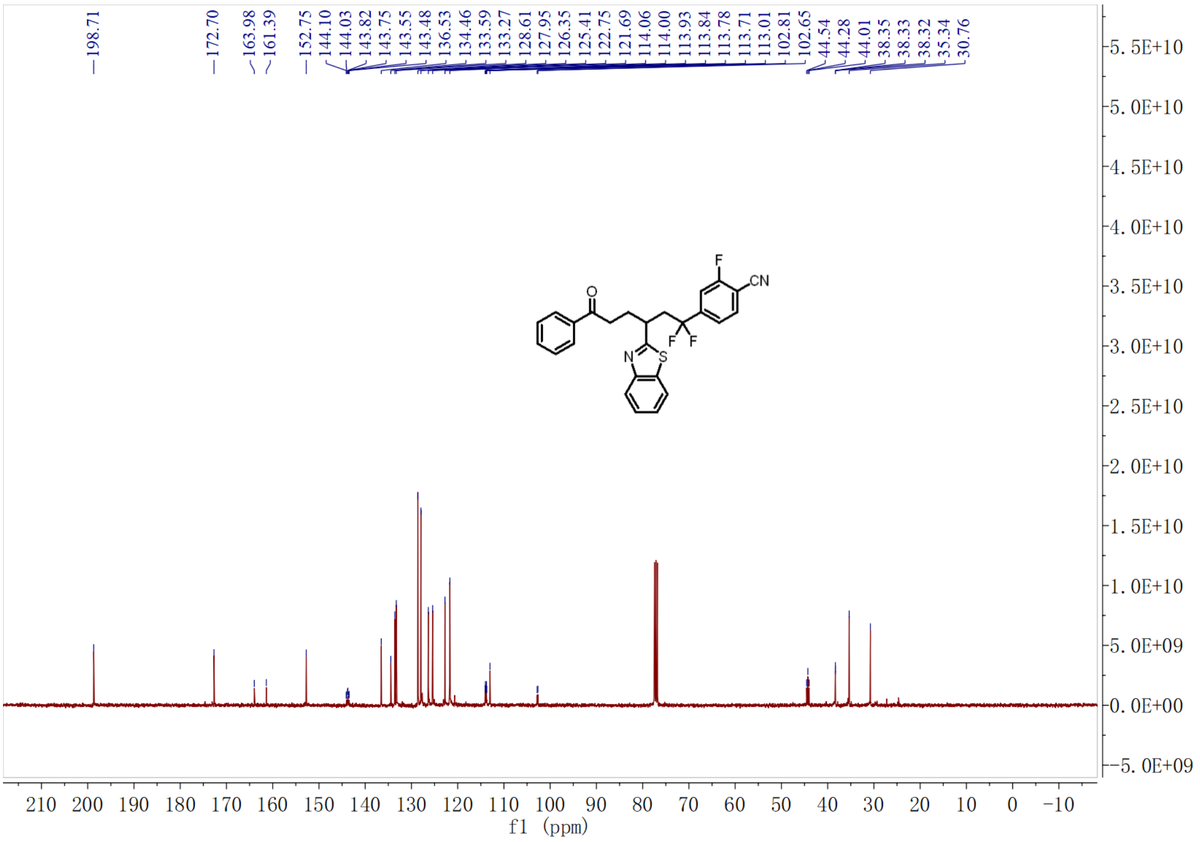

Supplementary Figure 77. 4b ^{1}H NMR (400 MHz, $CDCl_3$)

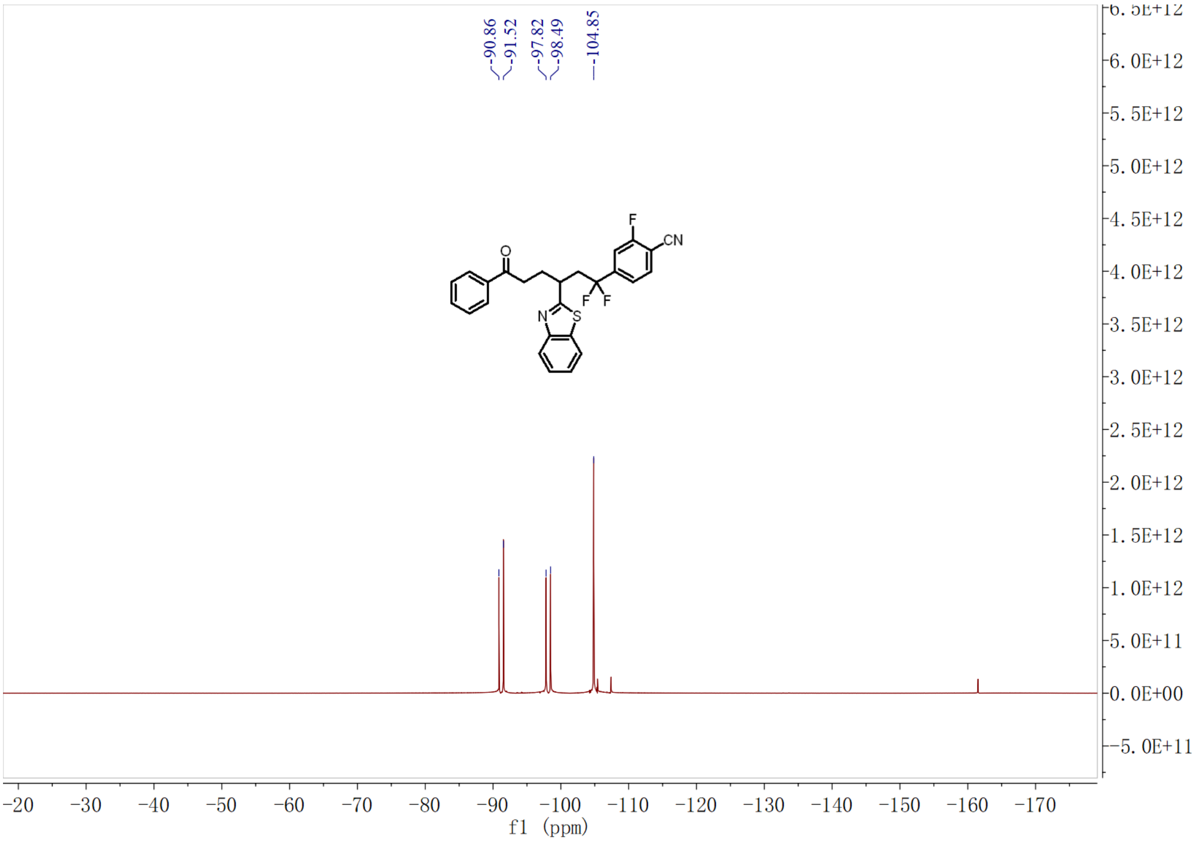

Supplementary Figure 78. 4b ^{13}C NMR (100 MHz, $CDCl_3$)

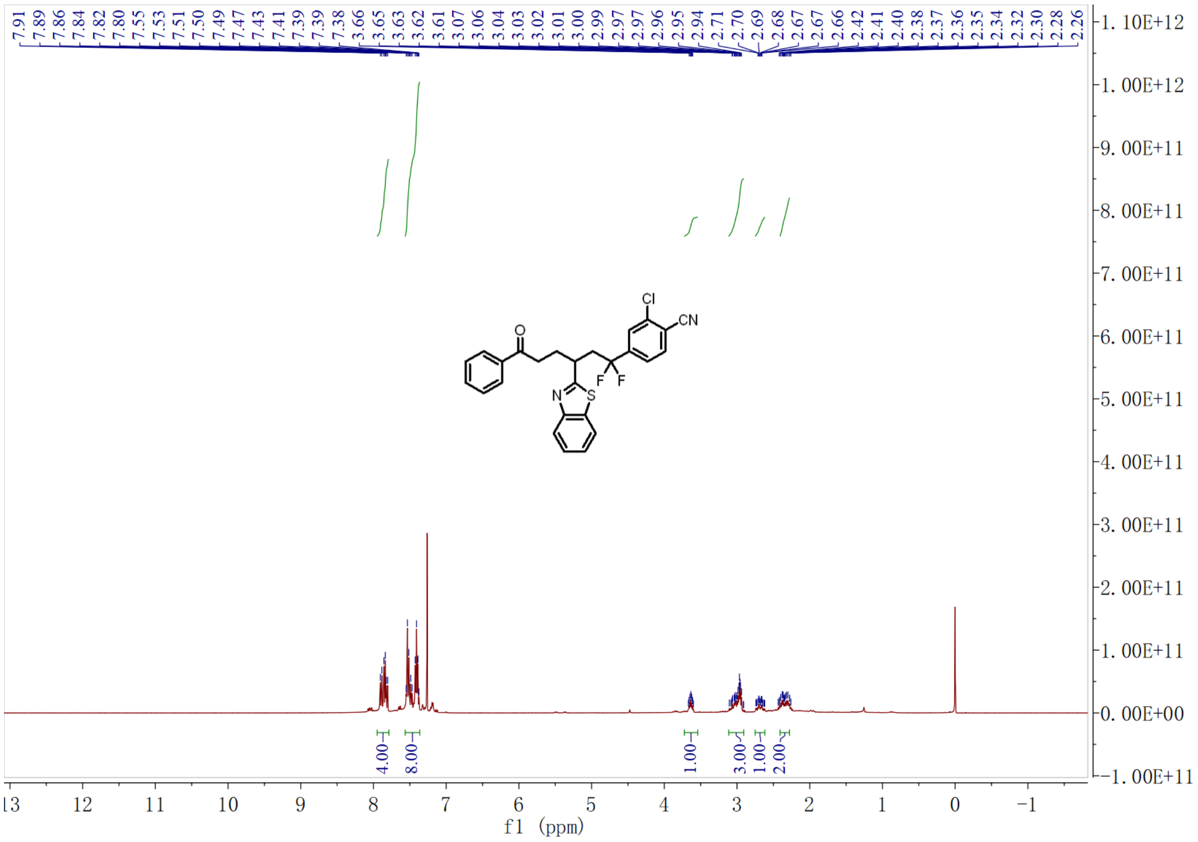

Supplementary Figure 79. 4b ^{19}F NMR (376 MHz, $CDCl_3$)

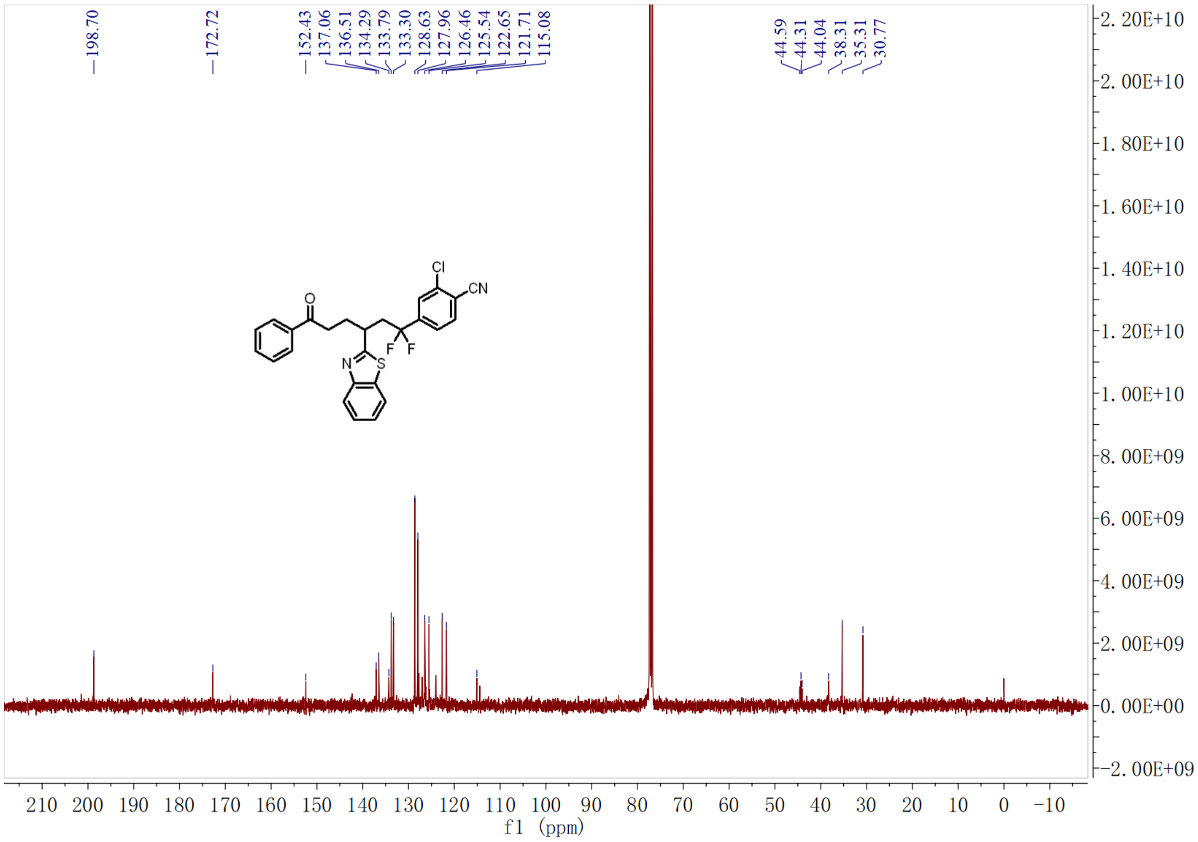

Supplementary Figure 80. 4c ^{1}H NMR (400 MHz, $CDCl_3$)

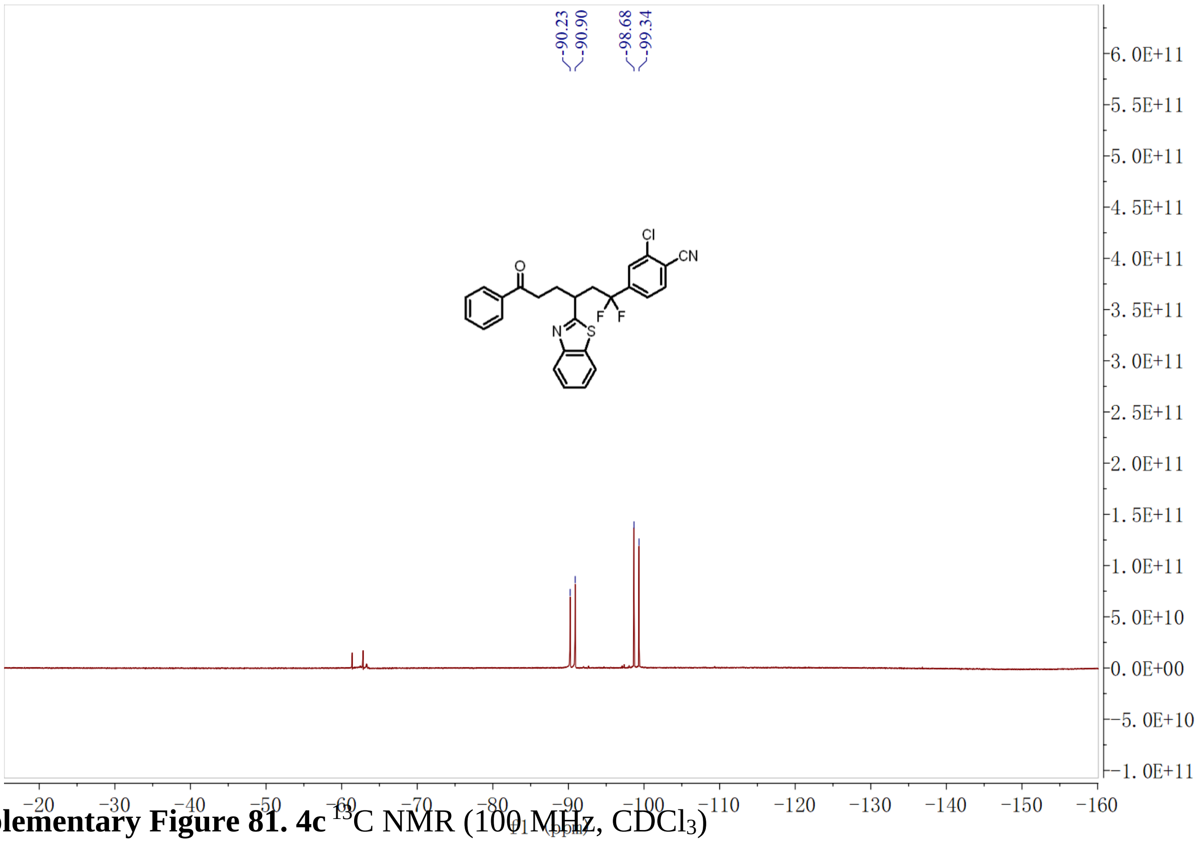

Supplementary Figure 81. 4c ^{13}C NMR (100 MHz, $CDCl_3$)

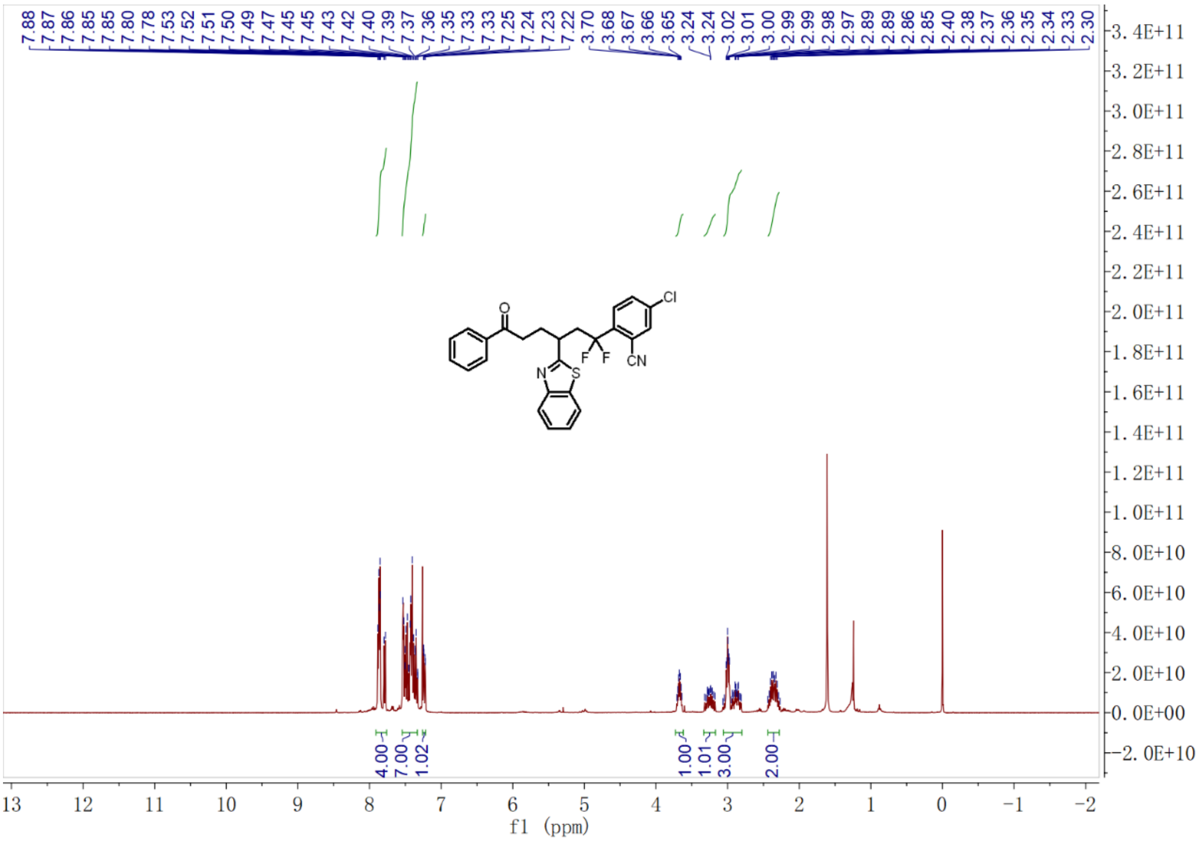

Supplementary Figure 82. 4c ^{19}F NMR (376 MHz, $CDCl_3$)

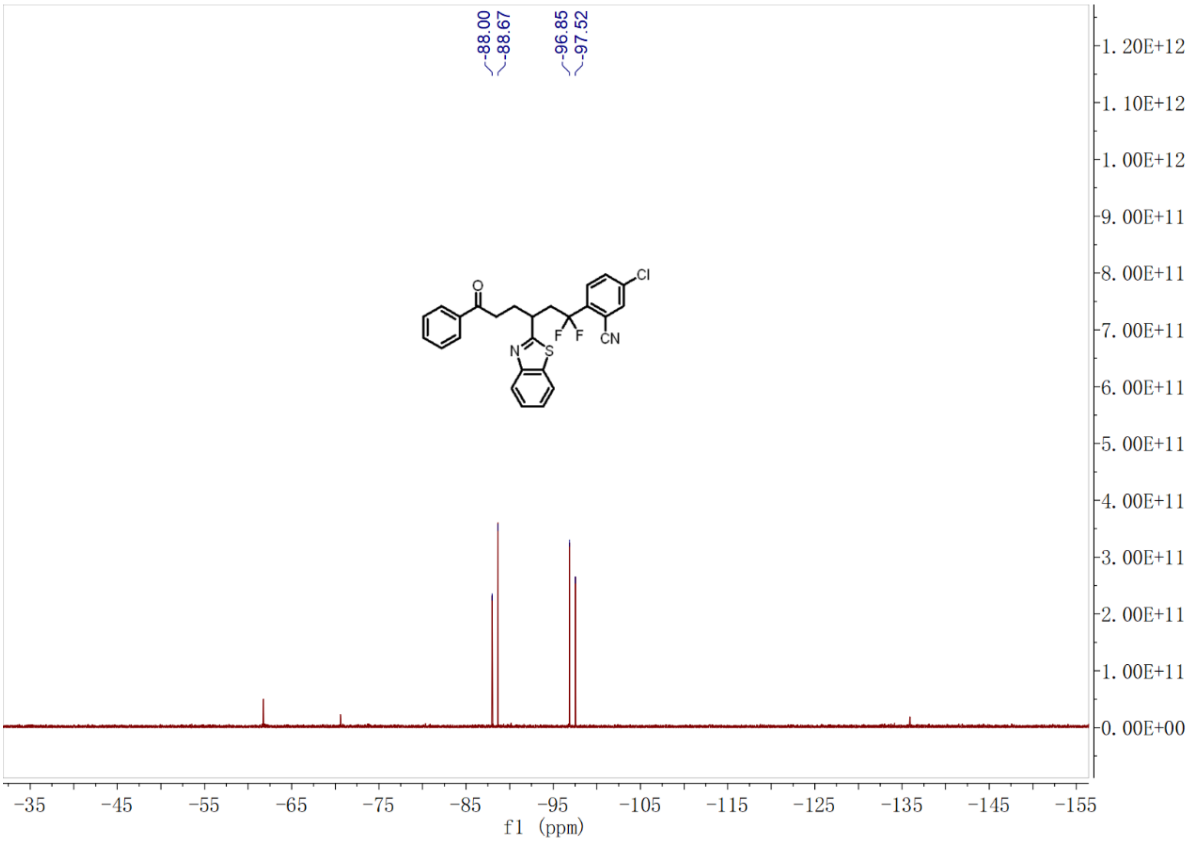

Supplementary Figure 83. 4g ^{1}H NMR (400 MHz, $CDCl_3$)

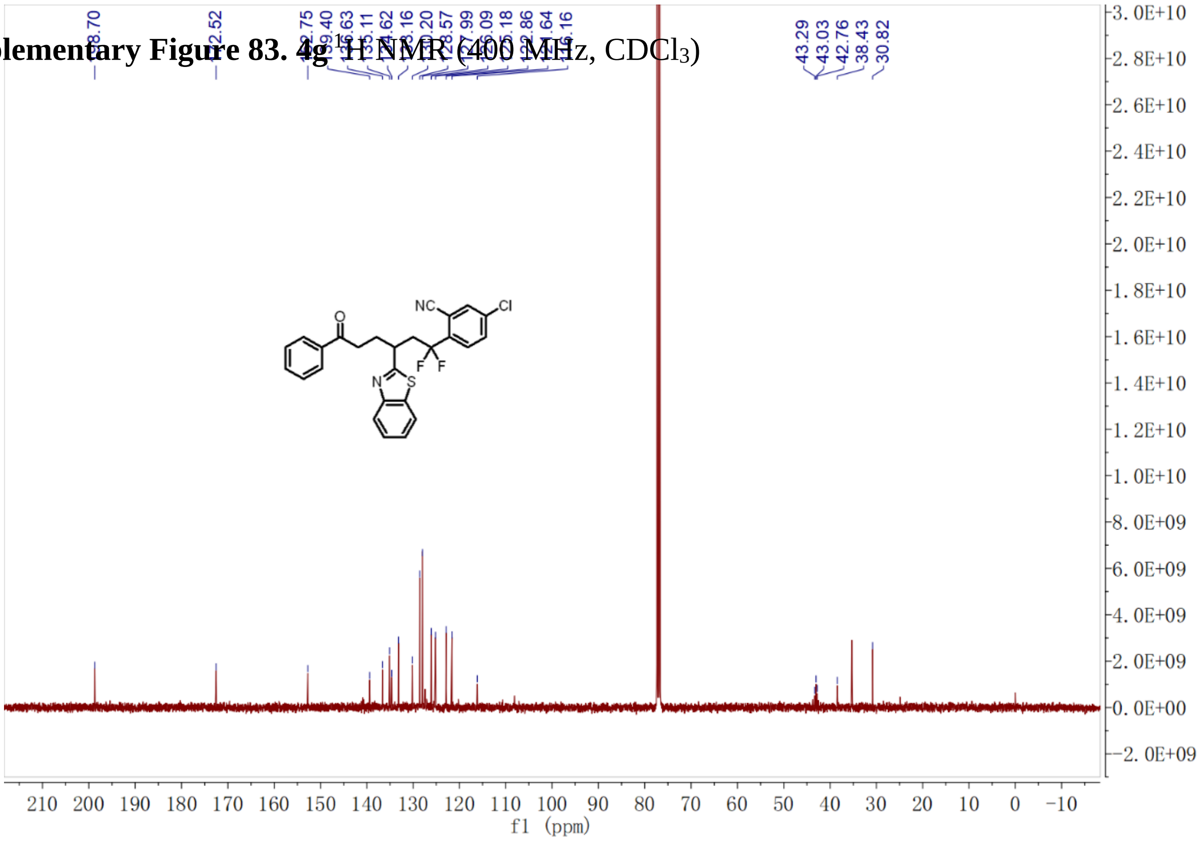

Supplementary Figure 84. 4g ^{19}F NMR (376 MHz, $CDCl_3$)

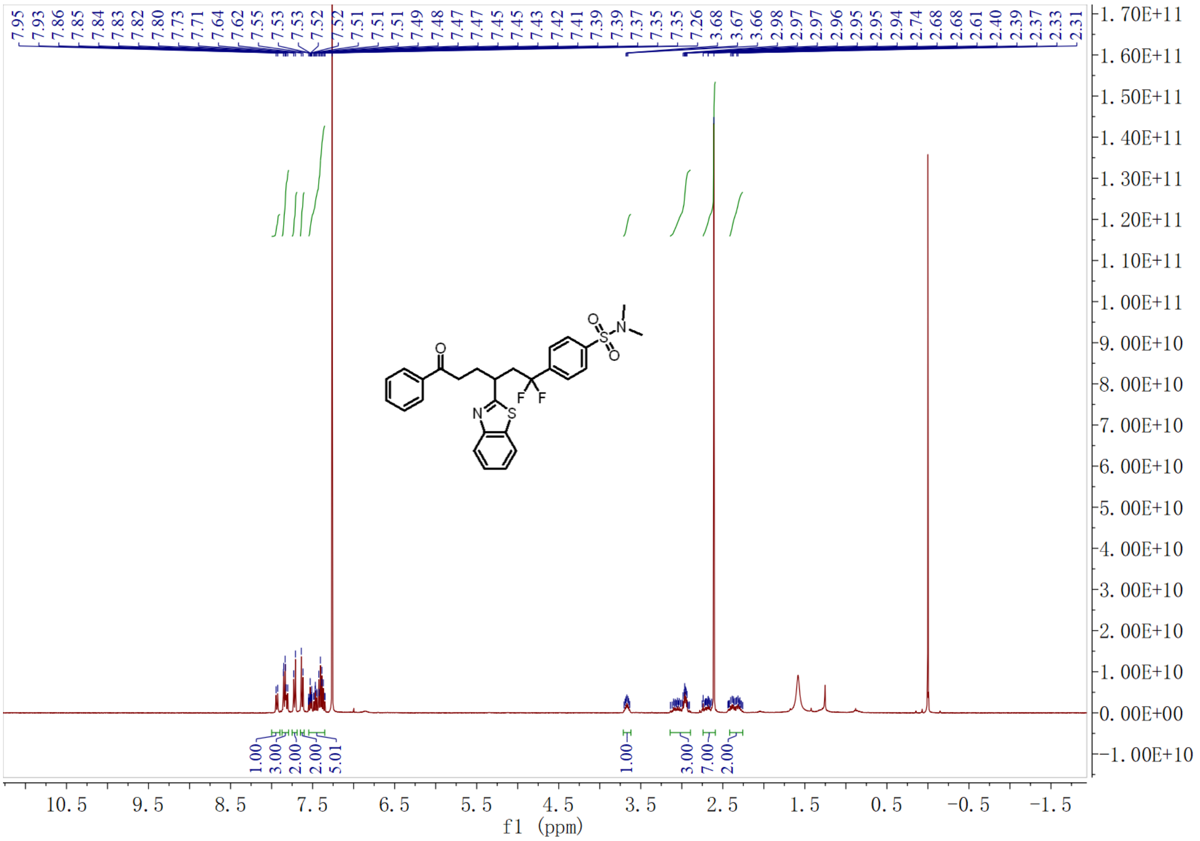

Supplementary Figure 85. 4g ^{13}C NMR (100 MHz, $CDCl_3$)

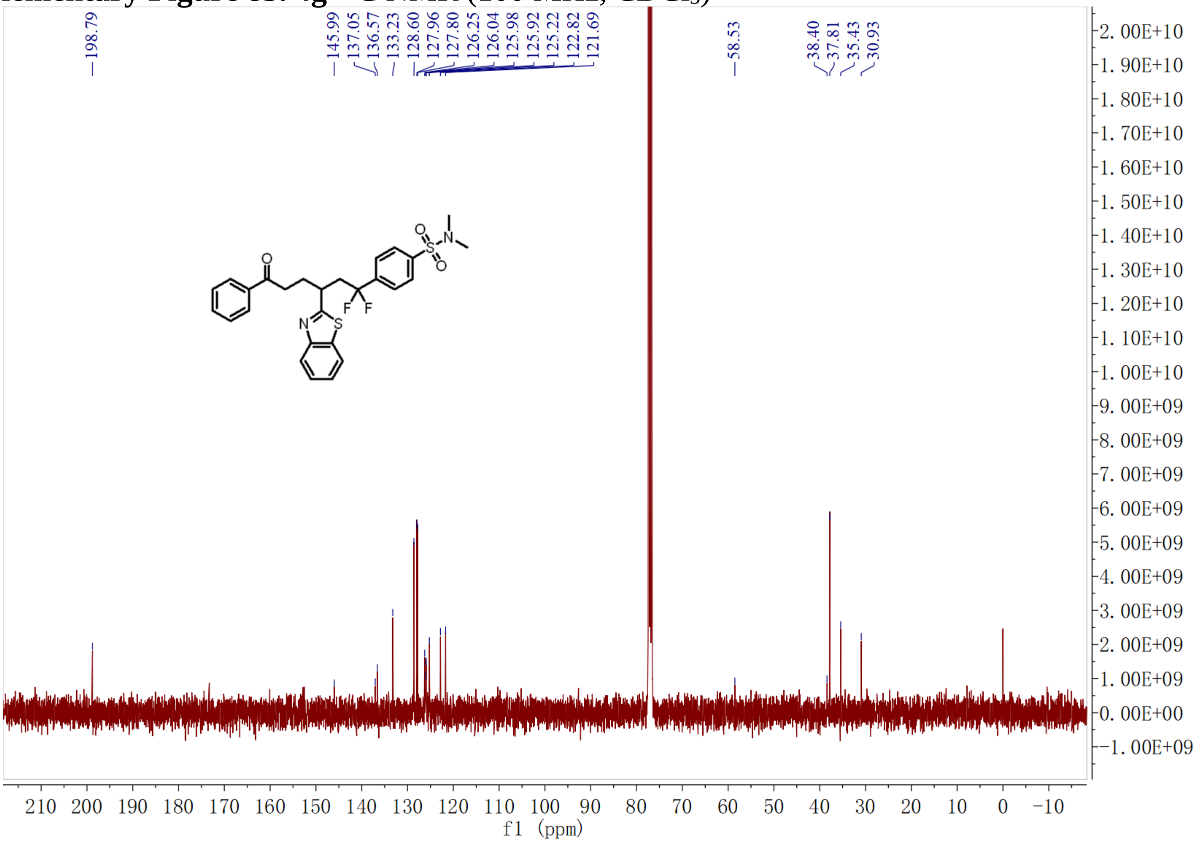

Supplementary Figure 86. 4h ^{1}H NMR (400 MHz, $CDCl_3$)

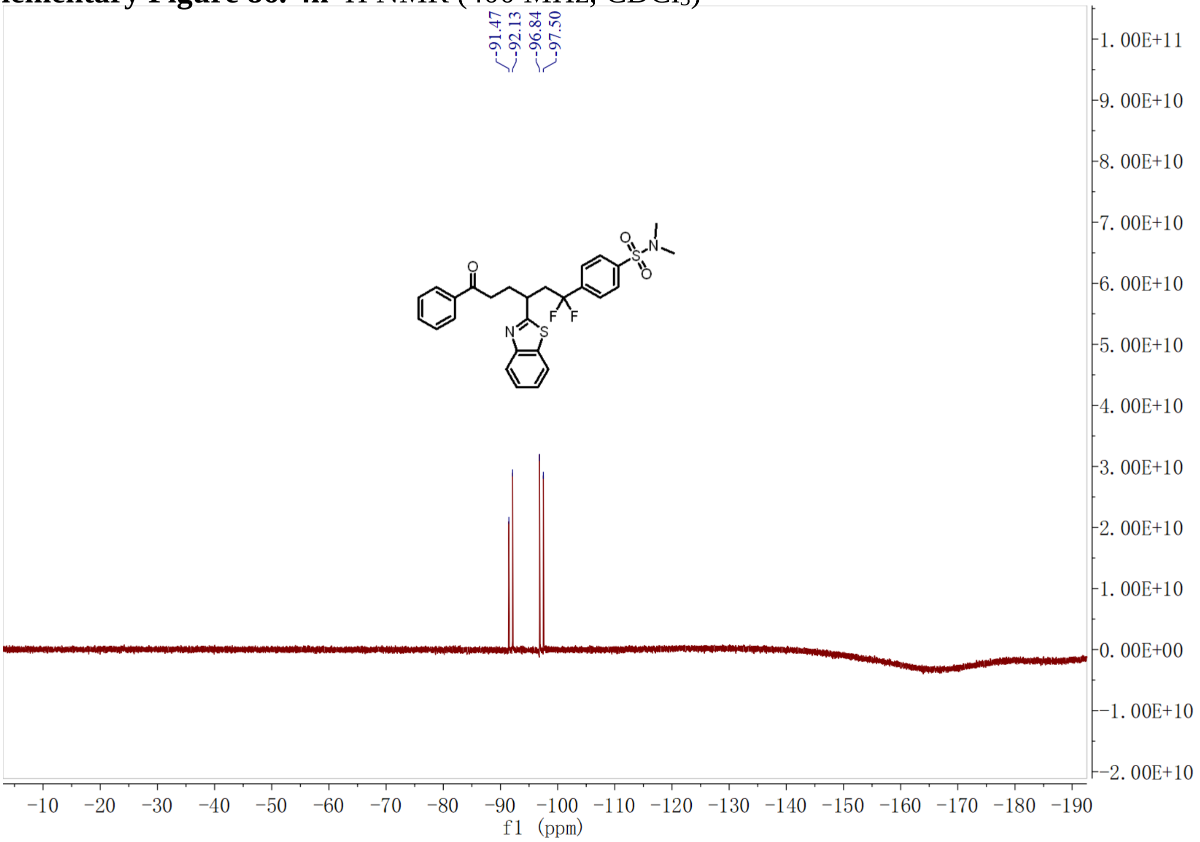

Supplementary Figure 87. 4h ^{13}C NMR (100 MHz, $CDCl_3$)

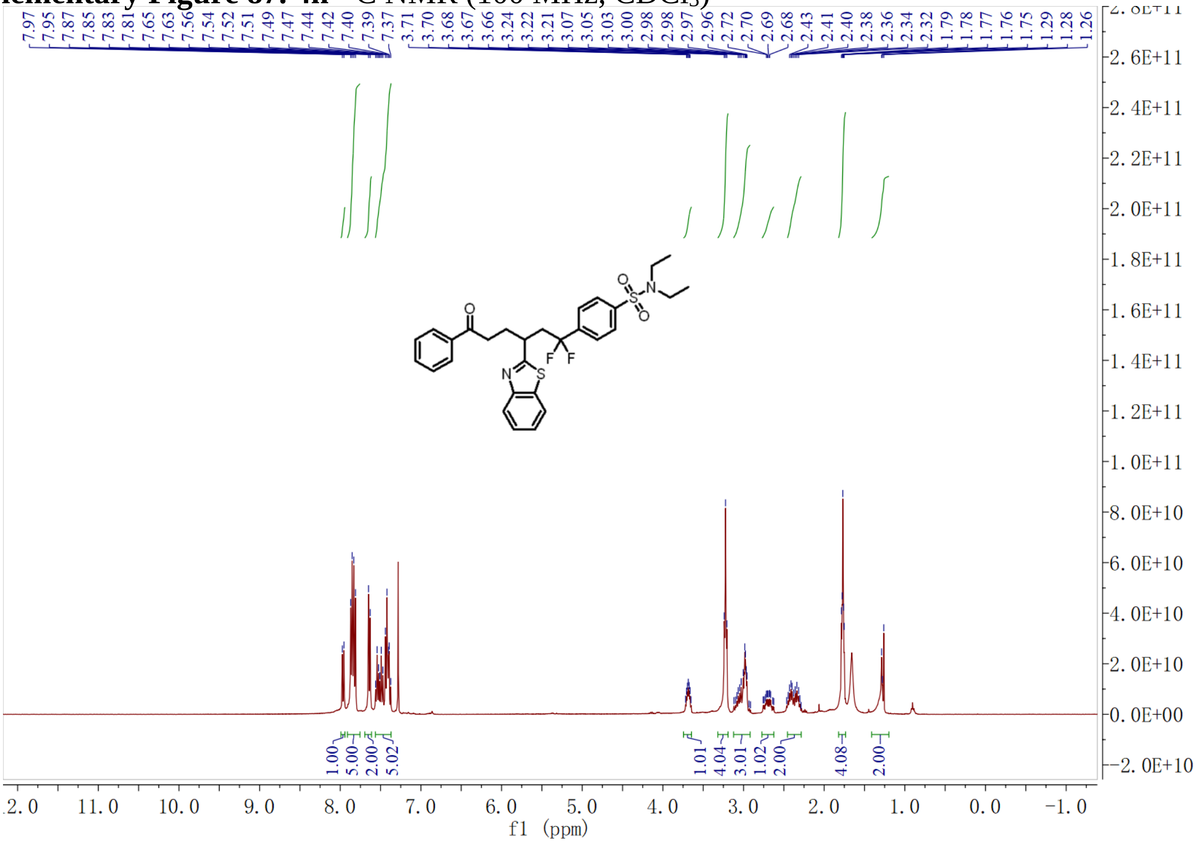

Supplementary Figure 88. **4h** ^{19}F NMR (376 MHz, $CDCl_3$)

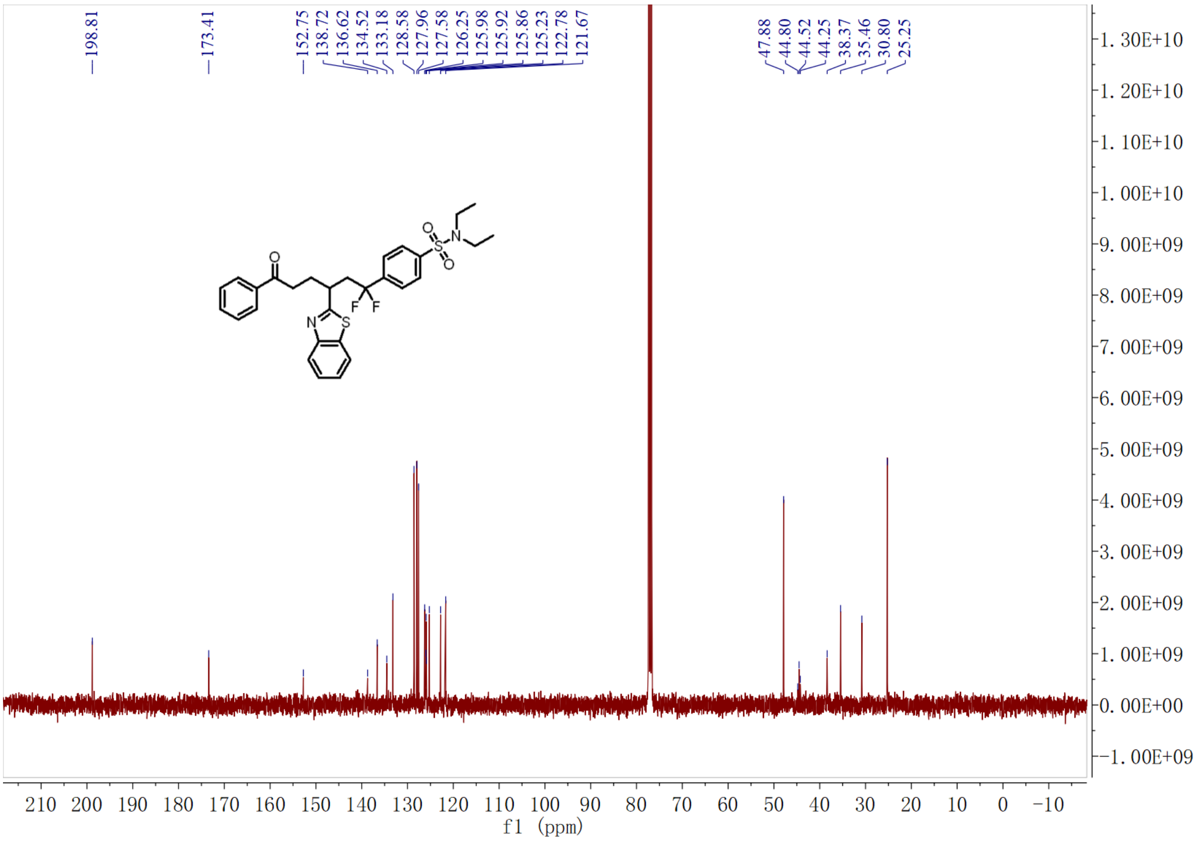

Supplementary Figure 89. **4i** ^{1}H NMR (400 MHz, $CDCl_3$)

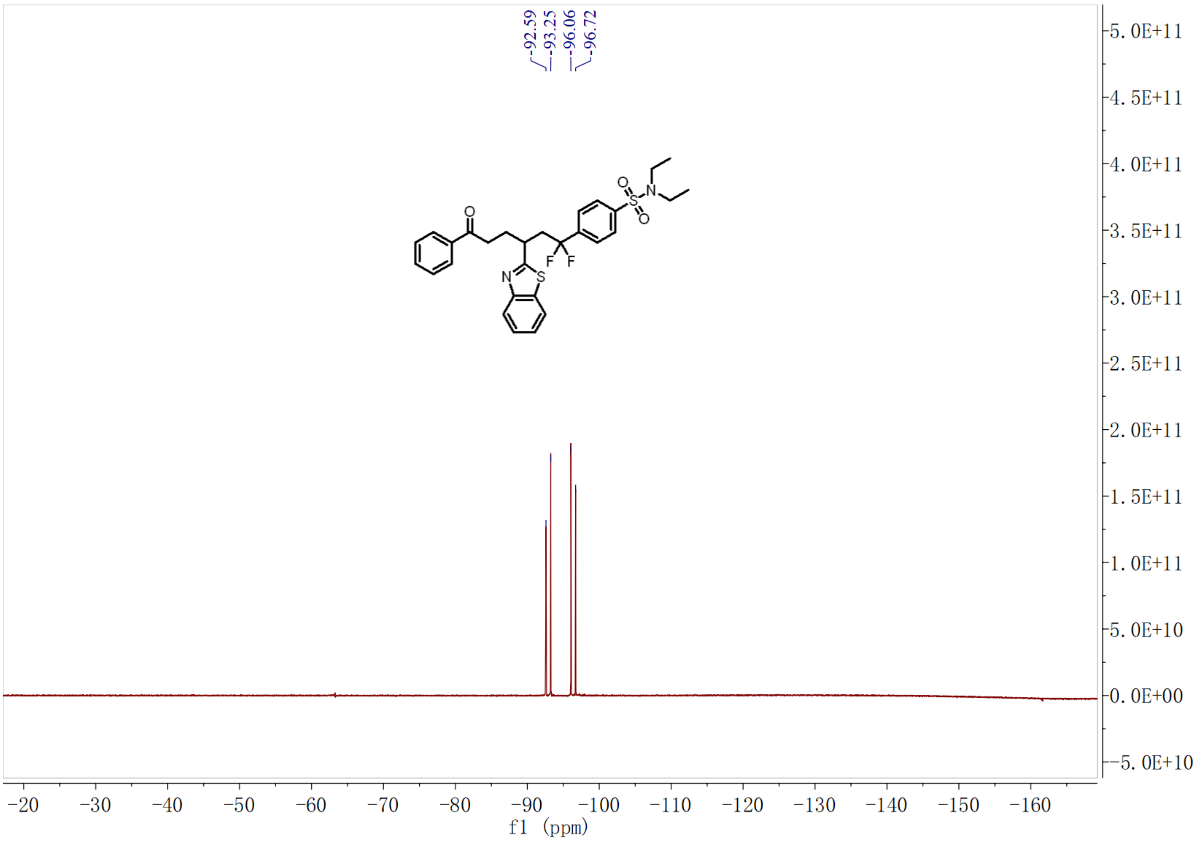

Supplementary Figure 90. 4i ^{13}C NMR (100 MHz, $CDCl_3$)

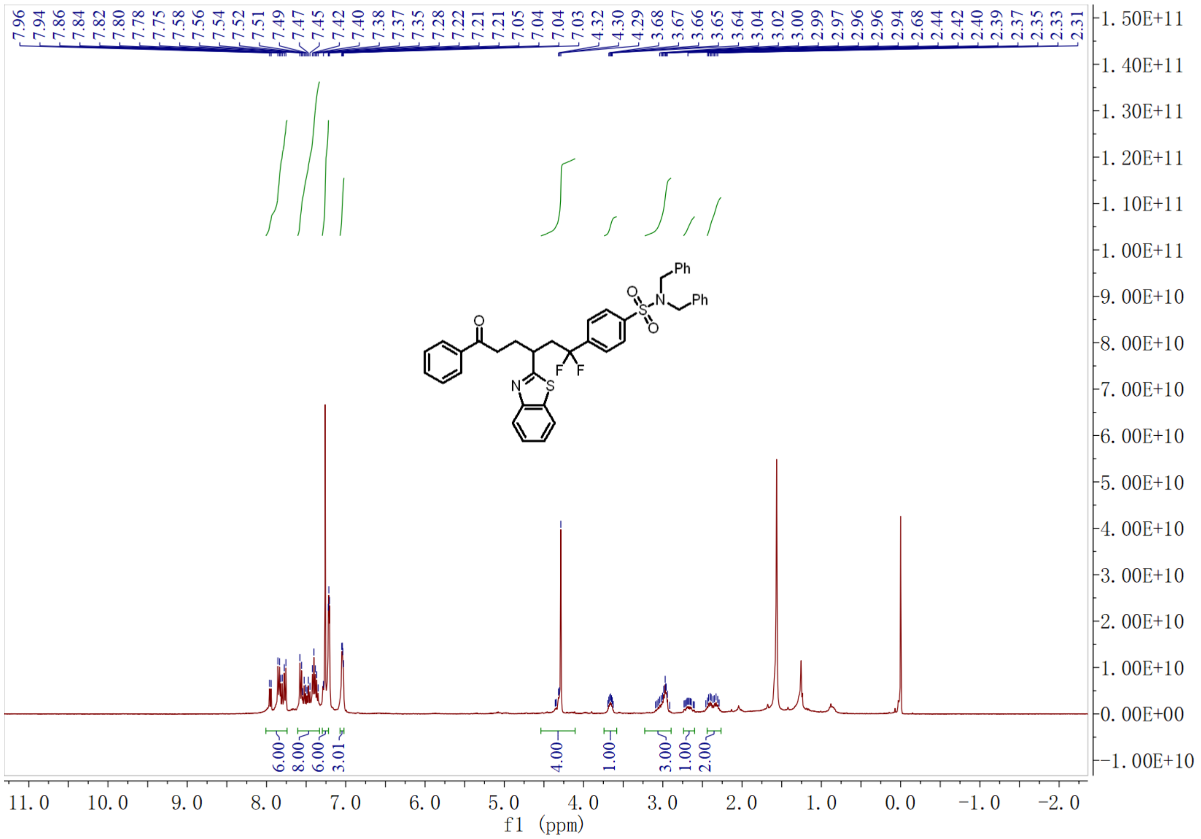

Supplementary Figure 91. 4i ^{19}F NMR (376 MHz, $CDCl_3$)

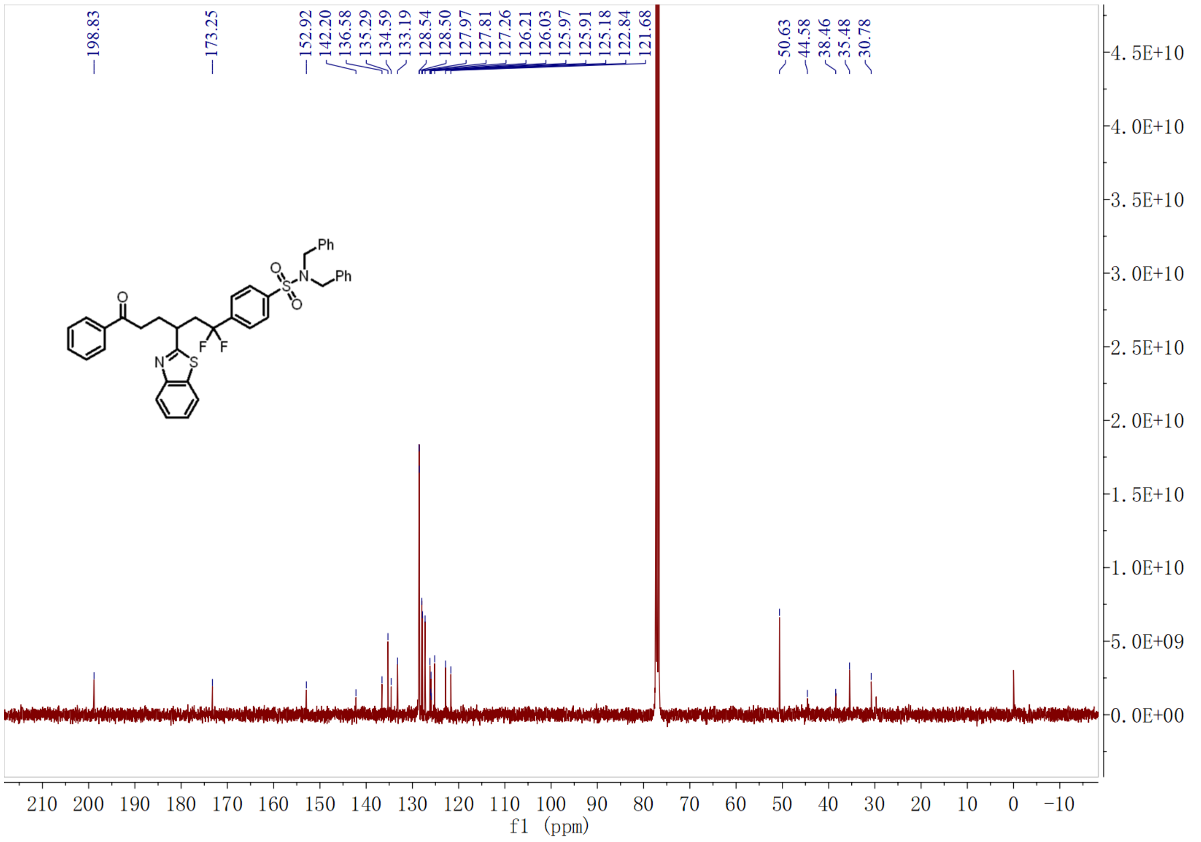

Supplementary Figure 92. 4j ^{1}H NMR (400 MHz, $CDCl_3$)

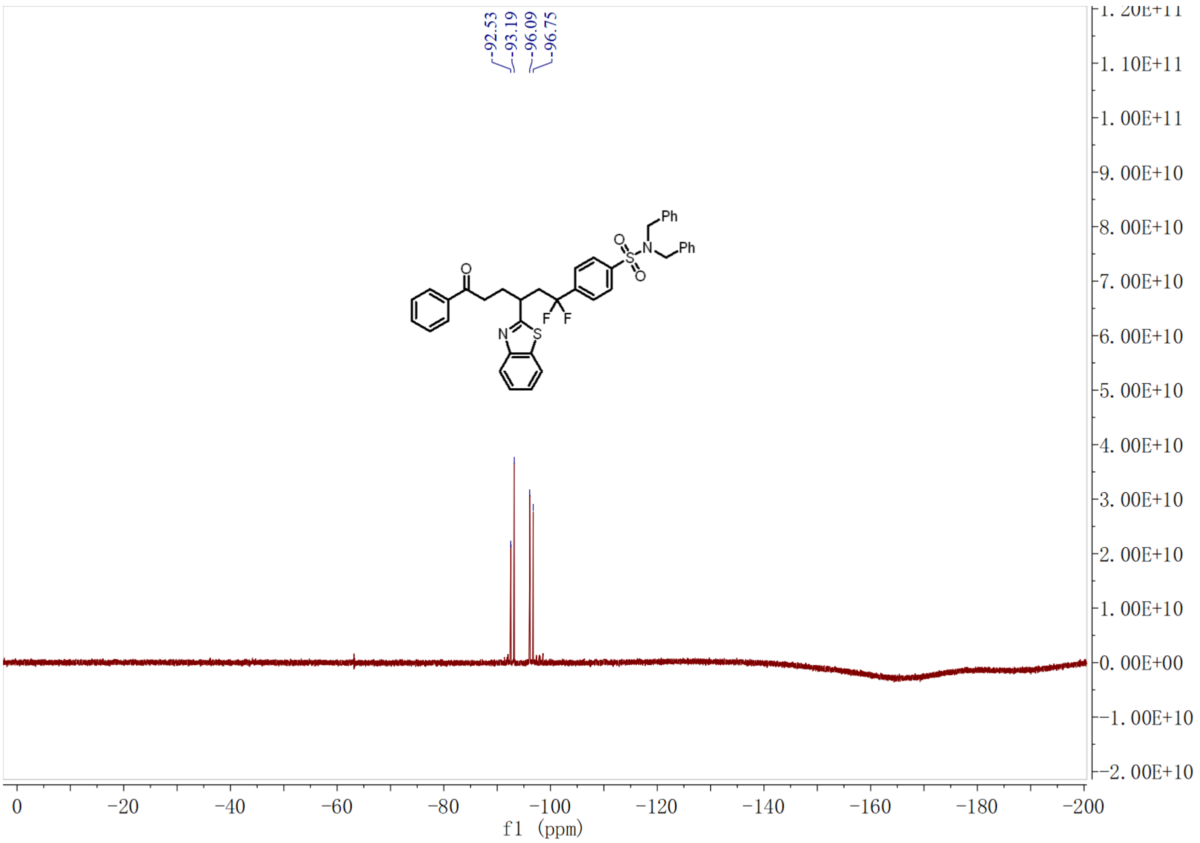

Supplementary Figure 93. 4j ^{13}C NMR (100 MHz, $CDCl_3$)

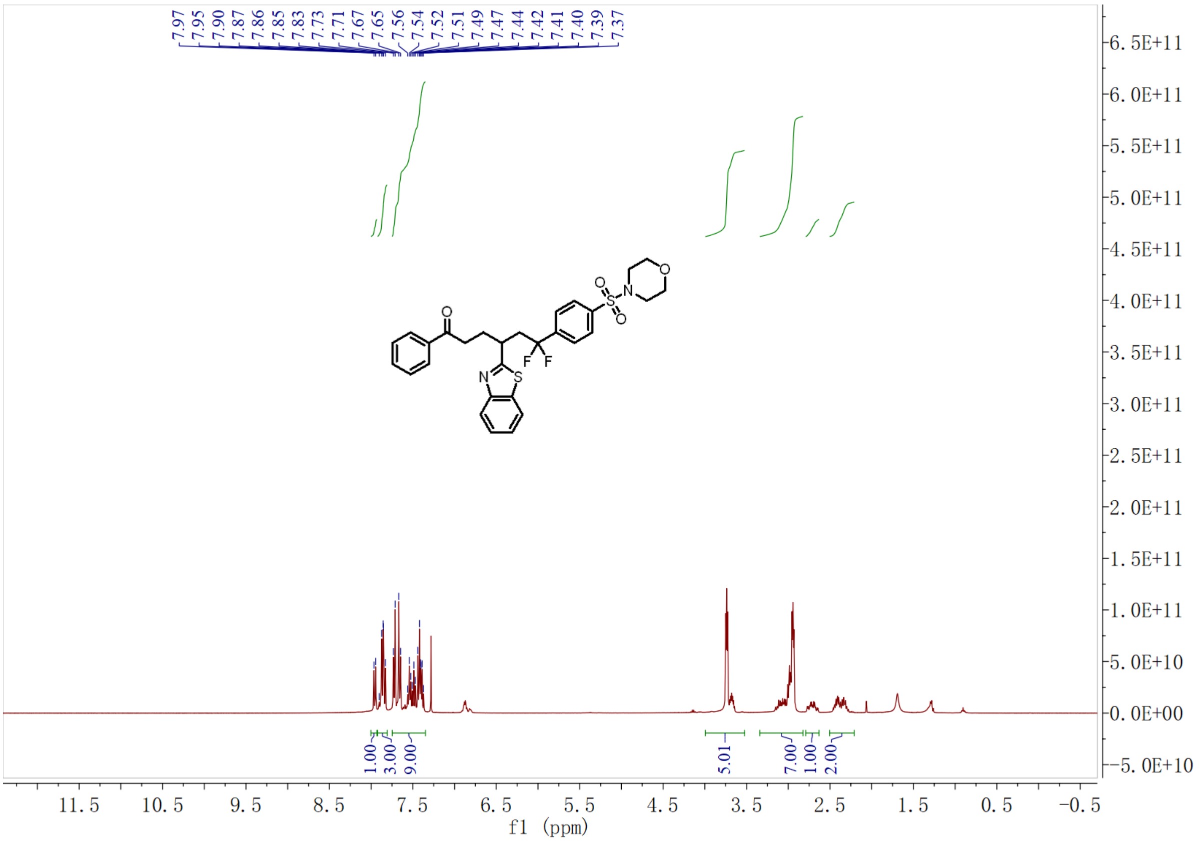

Supplementary Figure 94. 4j^{19}F NMR (376 MHz, $CDCl_3$)

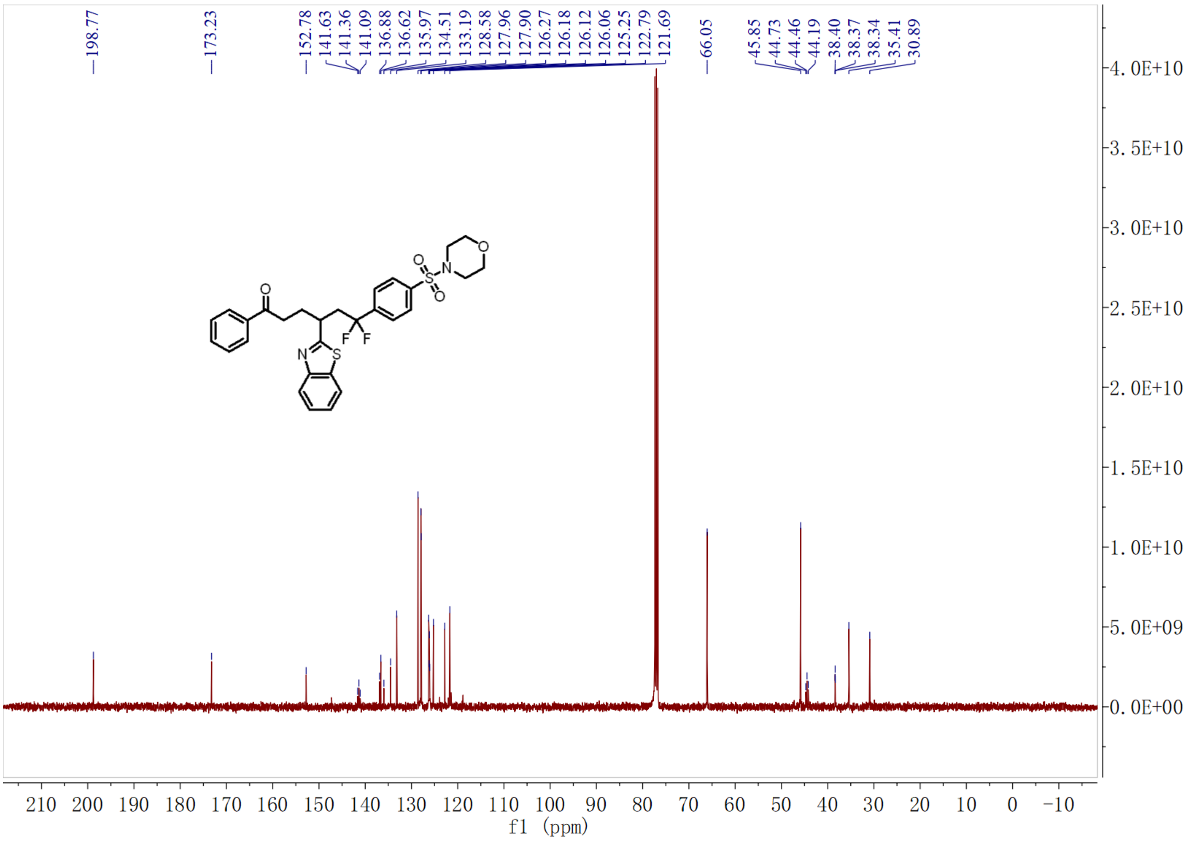

Supplementary Figure 95. 4k ^{1}H NMR (400 MHz, $CDCl_3$)

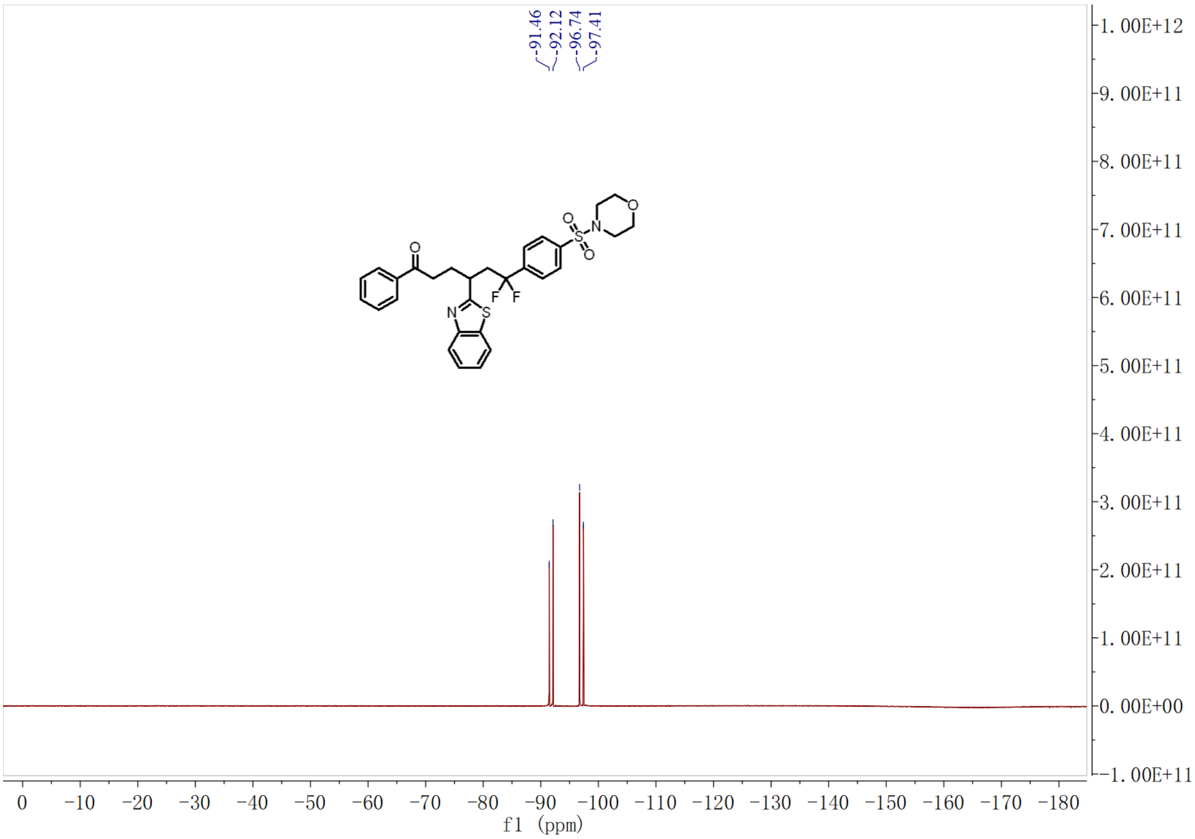

Supplementary Figure 96. 4k ^{13}C NMR (100 MHz, $CDCl_3$)

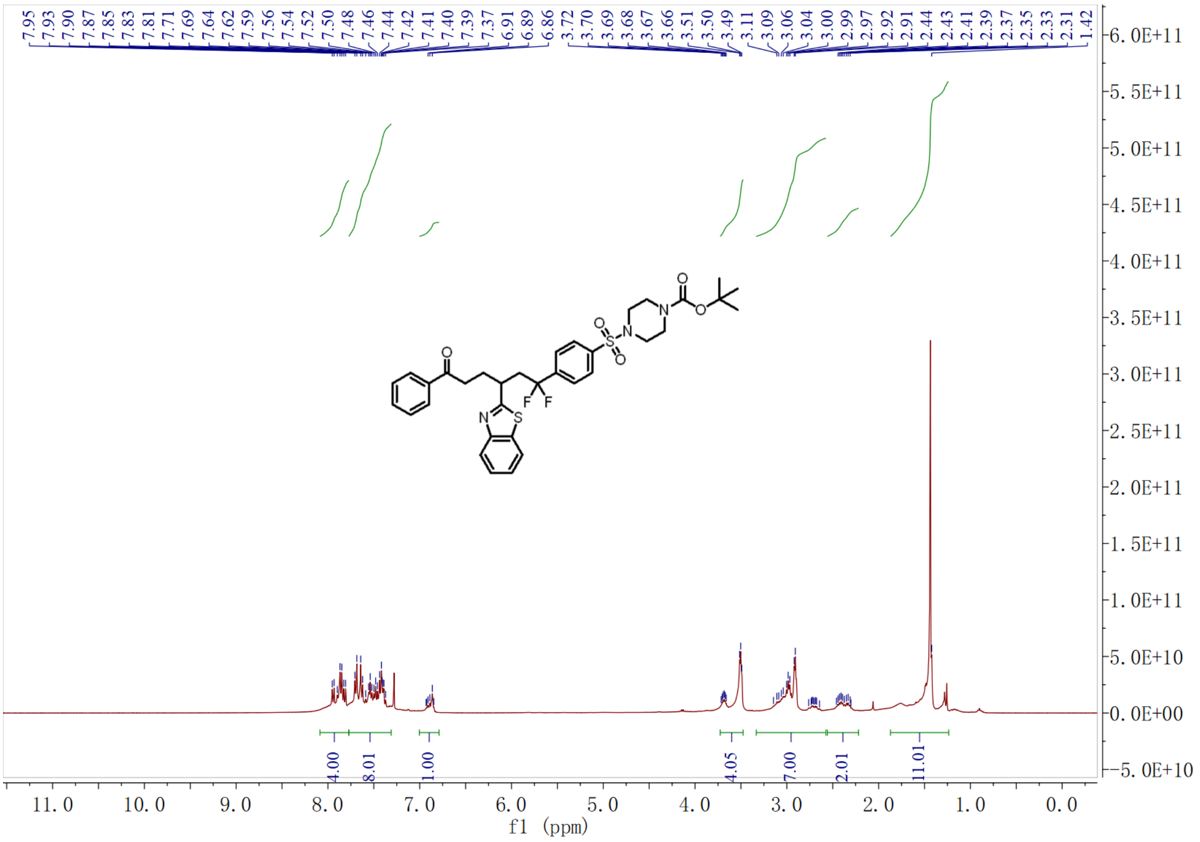

Supplementary Figure 97. 4k ^{19}F NMR (376 MHz, $CDCl_3$)

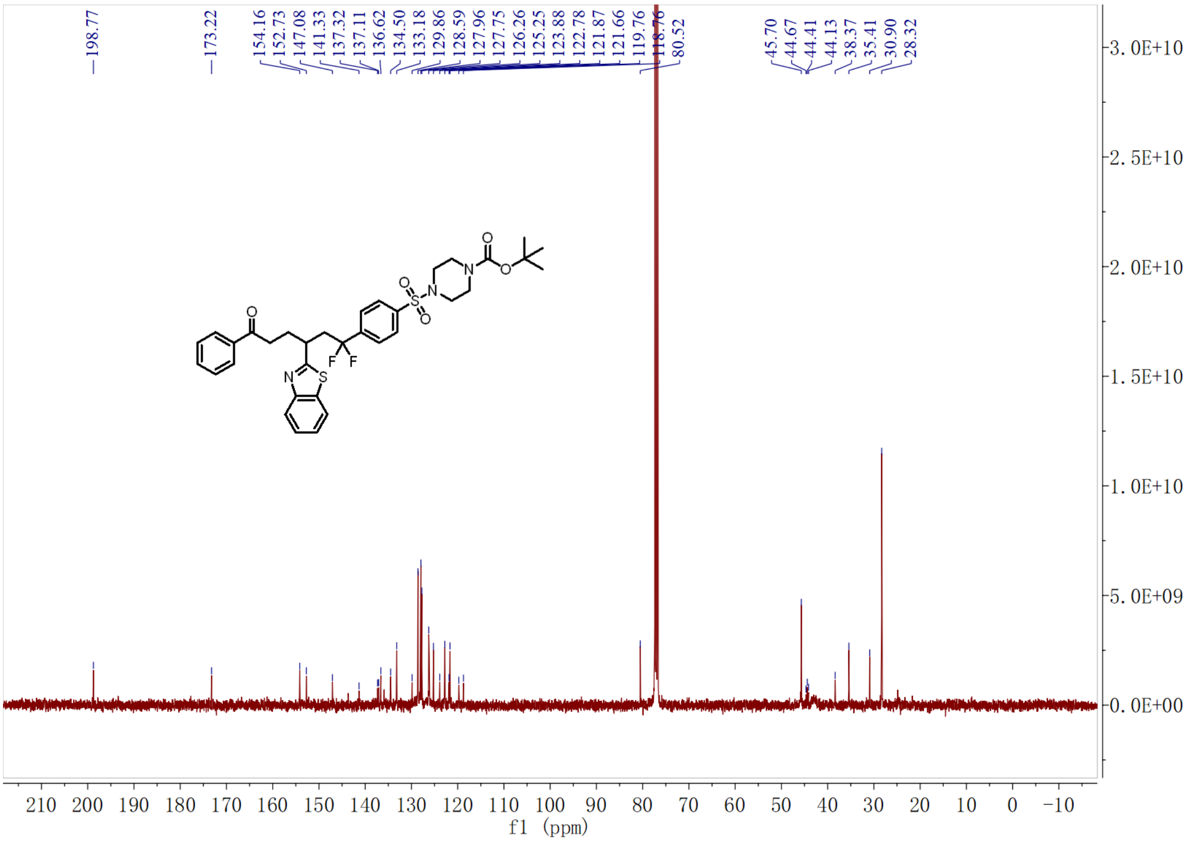

Supplementary Figure 98. 4l ^{1}H NMR (400 MHz, $CDCl_3$)

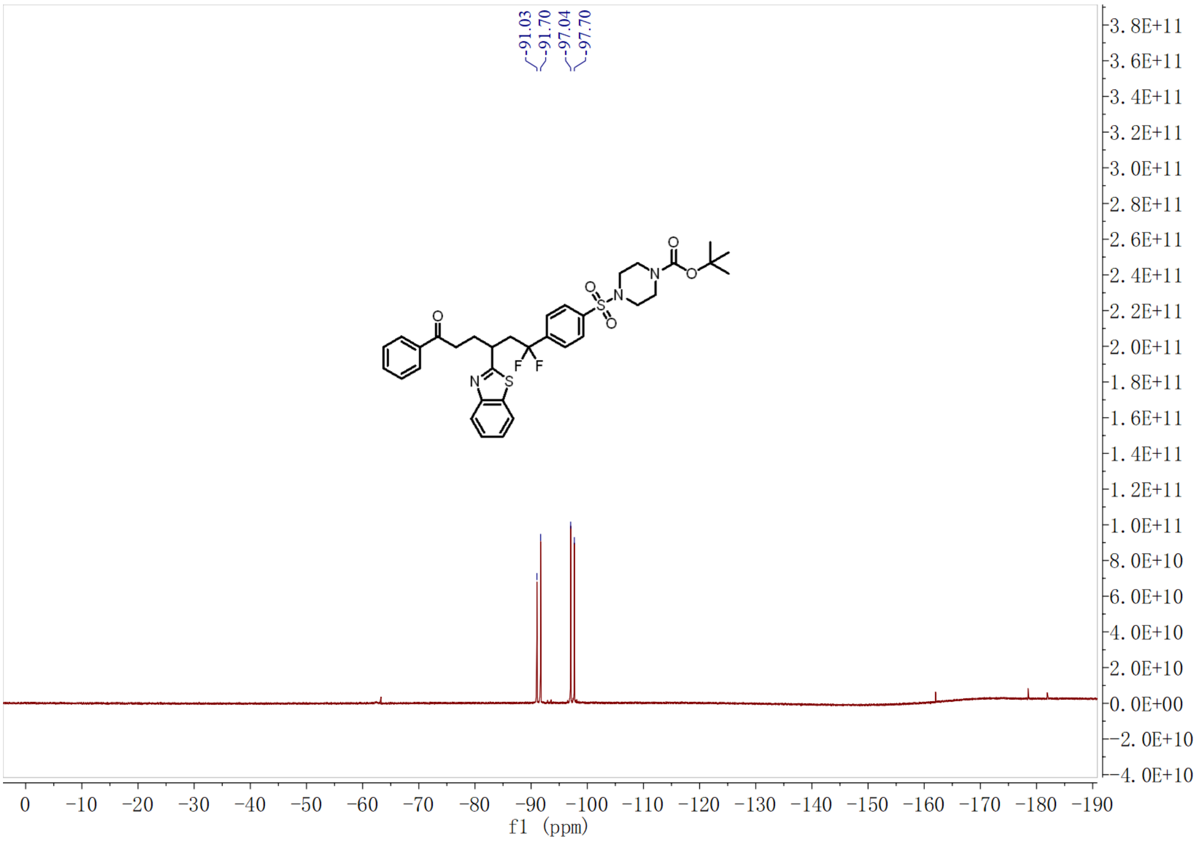

Supplementary Figure 99. 4l ^{13}C NMR (100 MHz, $CDCl_3$)

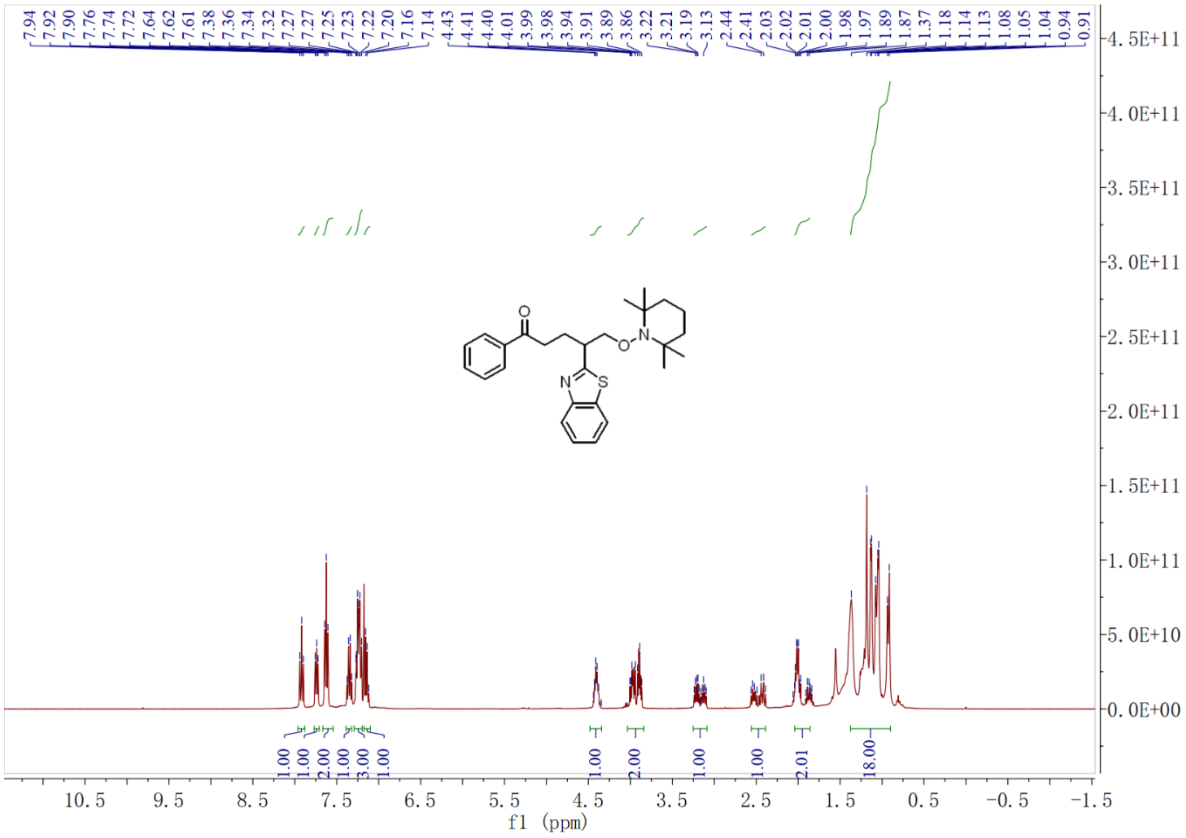

Supplementary Figure 100. 4l ^{19}F NMR (376 MHz, $CDCl_3$)

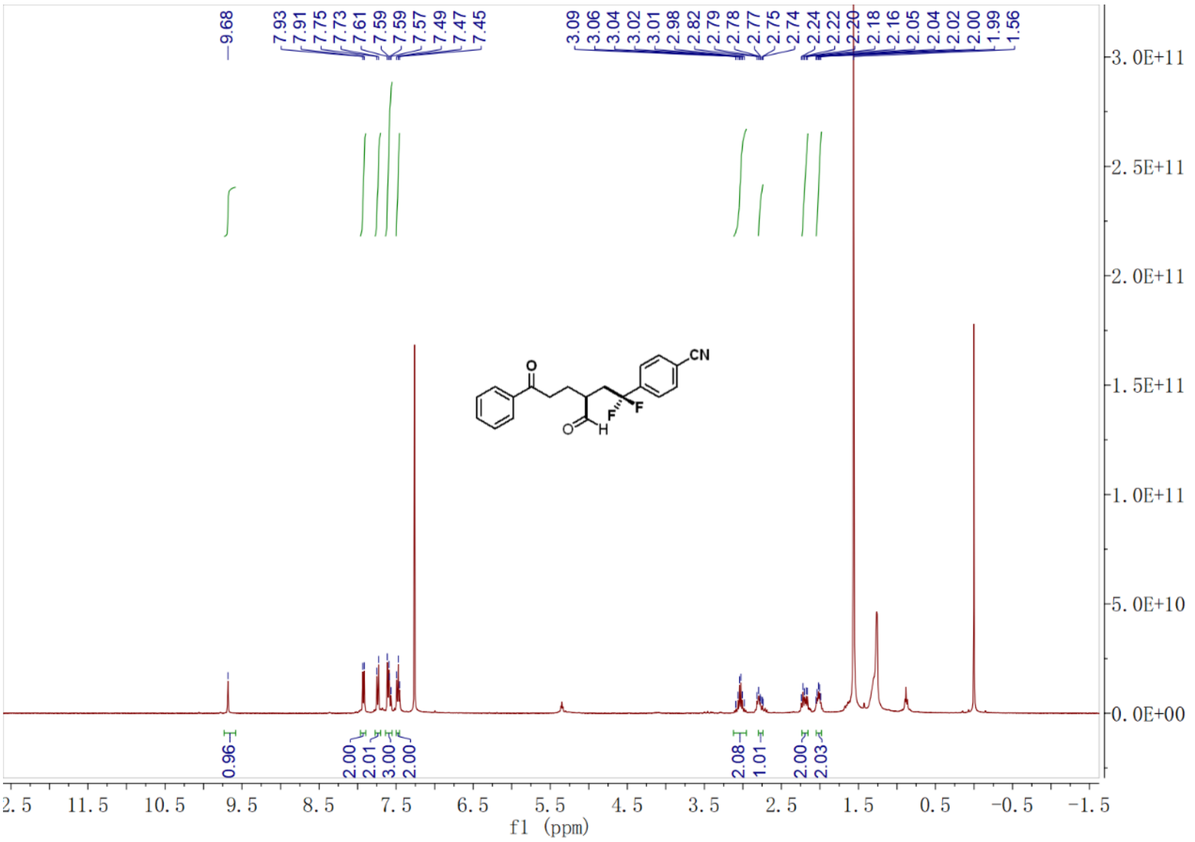

Supplementary Figure 101. 5a ^{1}H NMR (400 MHz, $CDCl_3$)

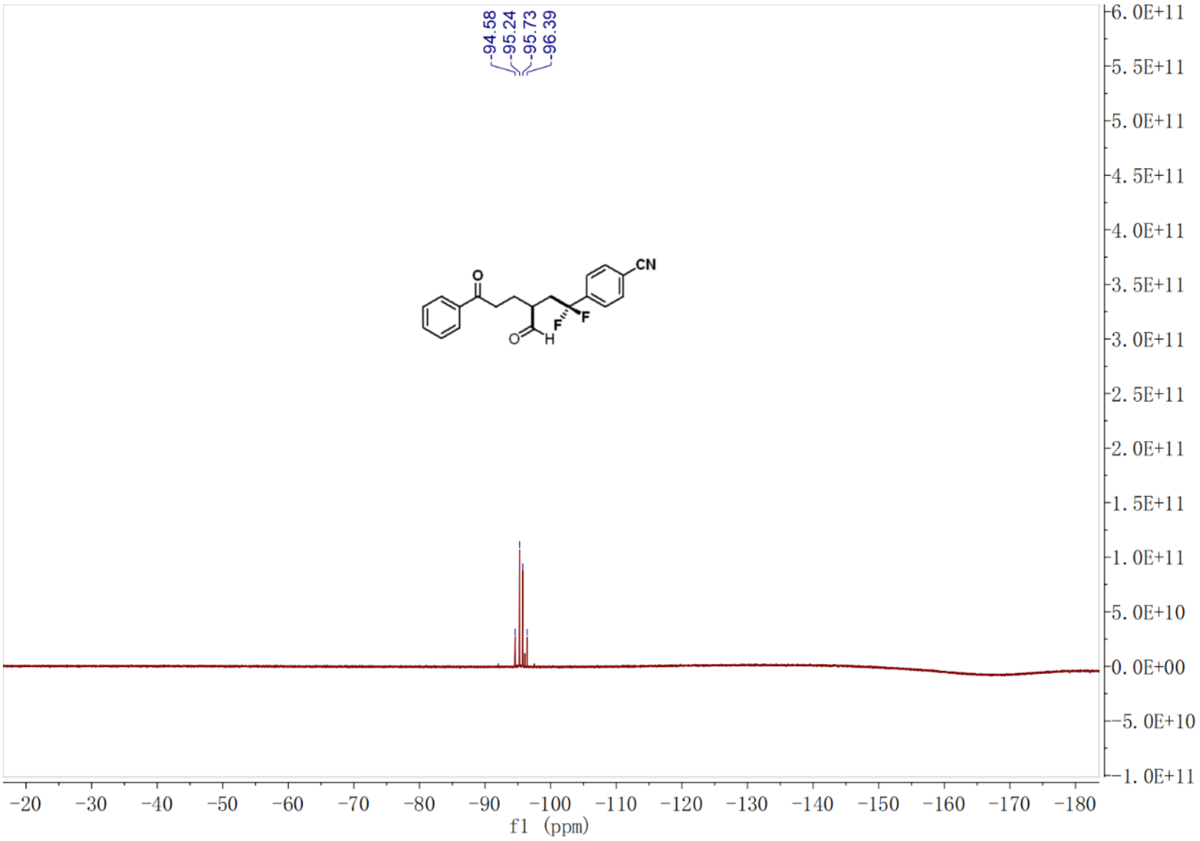

Supplementary Figure 102. 9 ^{1}H NMR (400 MHz, $CDCl_3$)

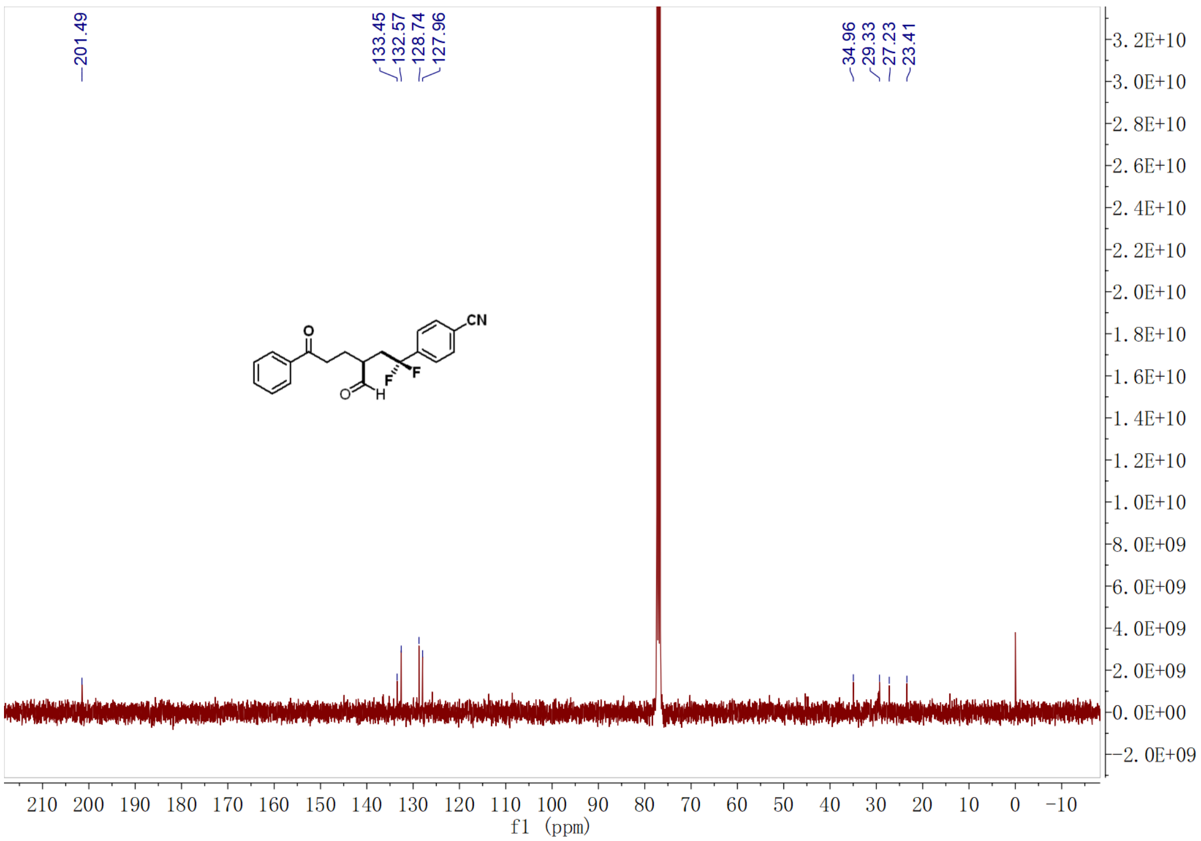

Supplementary Figure 103. 9 ^{19}F NMR (376 MHz, $CDCl_3$)

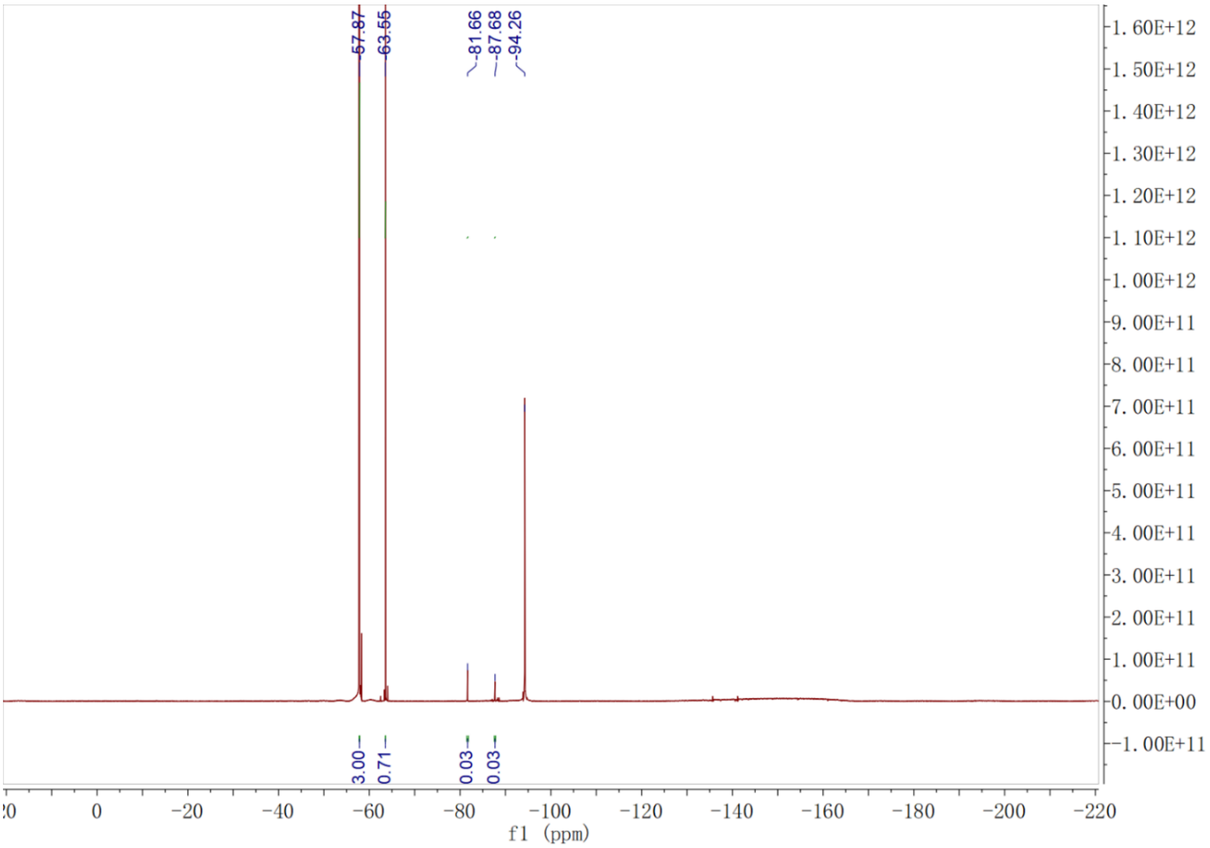

Supplementary Figure 104. 9 ^{13}C NMR (100 MHz, $CDCl_3$)

Supplementary Figure 105. Radical Trapping by 1,1-Diphenylethylene, ^{19}F NMR of cruder mixture (376 MHz, $CDCl_3$)

10. Supplementary References

1. Zheng, M., Yuan, X., Cui, Y.-S., Qiu, J.-K., Li, G.-g. & Guo. K. Electrochemical sulfonylation/ heteroarylation of alkenes via distal heteroaryl *ipso*-migration. *Org. Lett.* **20**, 7784-7789 (2018).
