# Supplementary material for: Photocatalytic radical defluoroalkylation of unactivated alkenes via distal heteroaryl ipso-migration
Source: Commun Chem. 2020 Aug 4;3:98. doi: 10.1038/s42004-020-00354-5 (PMC9814454; doi:10.1038/s42004-020-00354-5)
Supplement: Supplementary file 2 — Description of Additional Supplementary Files [file 42004_2020_354_MOESM2_ESM.pdf]

Description of Additional Supplementary Files

File Name: Supplementary Data 1
Description: crystallographic information file for compound **3a**.
